# A molecularly anchored spatial transcriptomic framework for precise CA1–subiculum parcellation and region-resolved analysis in Alzheimer’s disease

**DOI:** 10.1093/gigascience/giag094

**Published:** 2026-09-17

**Authors:** Yuyang Liu, Youzhe He, Yanrong Wei, Pan Wang, Chunyu Huang, Quyuan Tao, Langjian Zhu, Xun Xu, Longqi Liu, Shiping Liu, Lei Han, Jing Zhang, Lifang Wang

**Affiliations:** College of Life Sciences, University of Chinese Academy of Sciences, No.1 Yanqihu East Rd, Huairou District, Beijing 100049, China; State Key Laboratory of Genome and Multi-omics Technologies, BGI Research, 203 Zhenzhong Road, Xihu District, Hangzhou 310030, China; College of Life Sciences, University of Chinese Academy of Sciences, No.1 Yanqihu East Rd, Huairou District, Beijing 100049, China; State Key Laboratory of Genome and Multi-omics Technologies, BGI Research, 203 Zhenzhong Road, Xihu District, Hangzhou 310030, China; College of Life Sciences, University of Chinese Academy of Sciences, No.1 Yanqihu East Rd, Huairou District, Beijing 100049, China; State Key Laboratory of Genome and Multi-omics Technologies, BGI Research, 203 Zhenzhong Road, Xihu District, Hangzhou 310030, China; Department of Pathology, The First Affiliated Hospital, Zhejiang University School of Medicine, Hangzhou, Zhejiang 310002, China; College of Life Sciences, University of Chinese Academy of Sciences, No.1 Yanqihu East Rd, Huairou District, Beijing 100049, China; State Key Laboratory of Genome and Multi-omics Technologies, BGI Research, 203 Zhenzhong Road, Xihu District, Hangzhou 310030, China; College of Life Sciences, University of Chinese Academy of Sciences, No.1 Yanqihu East Rd, Huairou District, Beijing 100049, China; State Key Laboratory of Genome and Multi-omics Technologies, BGI Research, 203 Zhenzhong Road, Xihu District, Hangzhou 310030, China; State Key Laboratory of Genome and Multi-omics Technologies, BGI Research, 203 Zhenzhong Road, Xihu District, Hangzhou 310030, China; College of Life Sciences, University of Chinese Academy of Sciences, No.1 Yanqihu East Rd, Huairou District, Beijing 100049, China; BGI Research, Building 11, Beishan Industrial Zone, 146 Beishan Road, Yantian District, Shenzhen 518083, China; College of Life Sciences, University of Chinese Academy of Sciences, No.1 Yanqihu East Rd, Huairou District, Beijing 100049, China; Key Laboratory of Spatial Omics of Zhejiang Province, BGI Research, 203 Zhenzhong Road, Xihu District, Hangzhou 310030, China; College of Life Sciences, University of Chinese Academy of Sciences, No.1 Yanqihu East Rd, Huairou District, Beijing 100049, China; Key Laboratory of Spatial Omics of Zhejiang Province, BGI Research, 203 Zhenzhong Road, Xihu District, Hangzhou 310030, China; BGI Research, Building 11, Beishan Industrial Zone, 146 Beishan Road, Yantian District, Shenzhen 518083, China; Key Laboratory of Brain Cell Mapping of Zhejiang Province, 203 Zhenzhong Road, Xihu District, Hangzhou 310030, China; Department of Pathology, The First Affiliated Hospital, Zhejiang University School of Medicine, Hangzhou, Zhejiang 310002, China; State Key Laboratory of Genome and Multi-omics Technologies, BGI Research, 203 Zhenzhong Road, Xihu District, Hangzhou 310030, China; Key Laboratory of Spatial Omics of Zhejiang Province, BGI Research, 203 Zhenzhong Road, Xihu District, Hangzhou 310030, China

**Keywords:** spatial transcriptomics, hippocampus, CA1, subiculum, Stereo-seq, molecular parcellation, Alzheimer’s disease

## Abstract

**Background:**

The precise molecular delineation of the interface between the Subiculum (Sub) and cornu ammonis 1 (CA1) is a challenge in hippocampal research, as conventional cytoarchitectural boundaries are often ambiguous and limit reproducible regional annotation. Here, we developed a molecularly anchored spatial transcriptomic framework to define CA1–Sub regional identities using high-definition spatial transcriptomics (Stereo-seq) and single-nucleus RNA sequencing (snRNA-seq) references.

**Findings:**

Using a human hippocampal Stereo-seq dataset from 12 donors, we established a data-driven parcellation framework that defines reproducible molecular features distinguishing CA1 and Sub while capturing the transition between these regions. *FN1* was identified as a Sub-enriched marker in a subset of EX_Sub and, together with *ETV1* and additional regional markers, enabled molecular assignment of CA1 and Sub identities across datasets. The Sub association of *FN1* and *ETV1* was further supported by human 10X Genomics spatial transcriptomics, mouse *in situ* hybridization data, and a mouse spatial transcriptomic dataset. Applying this framework to Alzheimer’s disease tissues revealed region-specific transcriptional alterations across CA1 and Sub, including enrichment of mitochondrial energy metabolism-related transcripts in the Sub, suggesting exploratory transcriptional associations of altered metabolic function.

**Conclusions:**

This study provides a molecularly anchored framework for human CA1–Sub parcellation that complements conventional annotation. By defining regional molecular states while preserving the biological continuum across CA1–Sub interface, this approach enables more consistent regional analysis of human hippocampus tissue across donors, datasets, and disease conditions.

## Introduction

The hippocampus is a highly conserved structure composed of distinct anatomical regions, including the dentate gyrus (DG), cornu ammonis (CA1–CA4), and the Sub [1]. These regions are highly conserved during evolution [2], and form critical neural circuits essential for cognitive functions such as memory consolidation, spatial navigation, and emotional regulation [3]. As the primary output gateway of the hippocampal formation, the Sub is positioned between the CA1 region and the entorhinal cortex (EC) [4, 5]. It serves as a vital relay station, receiving convergent inputs from CA1 and various cortical areas while transmitting processed information to downstream cortical and thalamic structures [6–11]. This anatomical positioning renders the Sub an indispensable hub for information flow within and beyond the hippocampal system, playing a pivotal role in hippocampal-dependent learning and memory.

Despite these functional differences, the precise anatomical and functional boundary between Sub and CA1 remains poorly defined. Traditional histological markers often fail to capture the molecular transitions at this interface, leaving the precise anatomical–functional boundary poorly defined and hindering our understanding of region-specific contributions to hippocampal circuitry [12].

Precise spatial delineation is paramount for deciphering how the hippocampal formation segregates complex cognitive processing. While traditional transcriptomic methods provide deep cellular insights, they often lack the spatial context and architectural resolution required to resolve these fine-scale anatomical boundaries in the human brain. This lack of a standardized molecular reference prevents researchers from accurately attributing pathological changes, such as those seen in Alzheimer’s disease (AD), to specific hippocampal subfields, potentially masking region-specific disease mechanisms [13, 14].

Spatial and spatiotemporal transcriptomic technologies have been increasingly applied to reconstruct tissue architecture and organ-level molecular organization across developmental and disease contexts [15]. In this study, we present a high-definition molecularly anchored framework for human CA1–Sub parcellation. By integrating high-definition spatial transcriptomics (Stereo-seq) with single-nucleus RNA sequencing (snRNA-seq), we established a definitive molecular reference at near-cellular resolution. We report a validated marker panel, headlined by *FN1* and *ETV1*, which provides a reproducible toolkit for subfield annotation. This framework enables the CA1–Sub interface to be examined as a molecularly continuous yet regionally organized structure, rather than as a boundary defined solely by conventional histological landmarks. It also provides a standardized reference for comparing regional transcriptional profiles across individuals, platforms, and disease states. By applying this framework to AD samples, we further demonstrate its utility for resolving region-associated transcriptional heterogeneity while preserving anatomical context. These analyses are intended to provide a spatially grounded basis for future studies of hippocampal subfield vulnerability, circuit organization, and disease-associated molecular remodeling.

## Results

### A data-driven spatial framework resolves the ambiguous CA1–subiculum boundary

For decades, the precise border between the Subiculum (Sub) and CA1 regions of the hippocampus has remained poorly defined by classical anatomy [16, 17]. We addressed this long-standing challenge by developing a high-resolution molecularly anchored parcellation framework. To establish a molecularly anchored framework for precise CA1–Sub parcellation, we performed a secondary analysis of a previously published human hippocampal Stereo-seq spatial transcriptomic dataset (Stereo-seq) [18]. From the original spatial transcriptomic resource, we selected 12 specimens (6 AD and 6 Control) comprising 24 tissue sections based on tissue integrity, anatomical coverage, and the availability of an identifiable CA1–Sub region for downstream parcellation analyses (Fig. 1a, Supplementary Tables 1 and 2). The selected samples included patients with moderate-to-advanced AD (Braak III–V, with abundant Aβ deposition) and age- and sex-matched controls from individuals without dementia and with absent to low levels of AD neuropathology. Two independent tissue sections were available per donor in the original dataset, providing a robust spatial transcriptomic resource for secondary analysis in the present study.

To define the putative CA1 territory, we first leveraged metallophosphoesterase domain containing 1 (*MPPED1*) as a known CA1 marker, observing its stable expression across all samples (Fig. 1b and c, Extended Data Fig. 1a) [19]. Based on *MPPED1* expression [18], we extracted the broad CA1 region from each section (Fig. 1a). While this initial demarcation aligned with traditional landmarks, unsupervised clustering (BayesSpace) within the *MPPED1*-defined CA1 unveiled 2 transcriptionally distinct domains (Fig. 1d, Extended Data Fig. 1b) [20]. This data-driven parcellation indicates that tissue annotated as a single anatomical field by conventional landmarks contains reproducible molecular heterogeneity, suggesting the presence of a latent boundary at the Sub–CA1 interface.

**Figure 1:**
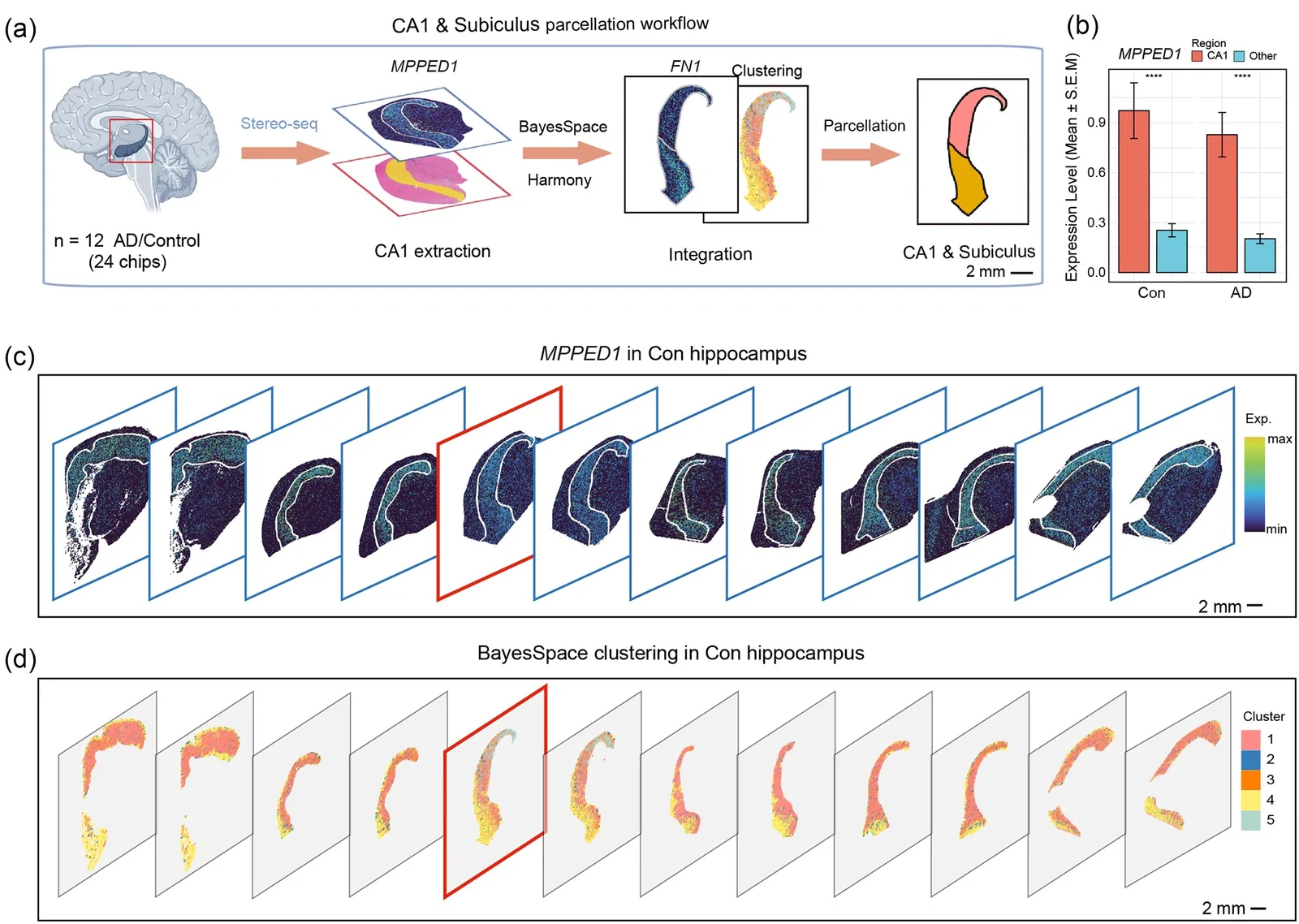
Region parcellation of human hippocampus by gene expression pattern. (a) The schematic diagram of the study design. A total of 12 samples (24 tissue sections) from AD and Con individuals are performed sequencing. Stereo-seq tissue sections were cut coronally through the hippocampal body. (b) Quantitative statistics of the expression level of *MPPED1* gene in CA1 and other brain regions. Data are presented as the mean ± standard error of the mean (SEM). Difference between CA1 and other regions was examined by *t*-test with Holm’s correction, ^****^  *P* < 0.0001. The “Other brain regions” refer to the non-CA1 hippocampal subfields captured on the same Stereo-seq chips, specifically DG, CA2–4, FAS (fimbria, alveus, and stratum oriens [SO]) and SLRM (stratum lucidum [SL], stratum radiatum [SR], stratum moleculare [SM]). (c) The expression of *MPPED1* gene in hippocampal CA1 brain region of Con individuals. All Con individuals are age- and sex-matched controls without dementia or hippocampal pathology. The chip marked with a red circle contained 2 brain regions of relatively moderate size, and this chip would serve as the primary subject for display in the main figures. (d) The gene expression of internal heterogeneity in the Con CA1 regions based on BayesSpace algorithm. Scale bar: 2 mm.

**Extended Data Figure 1:**
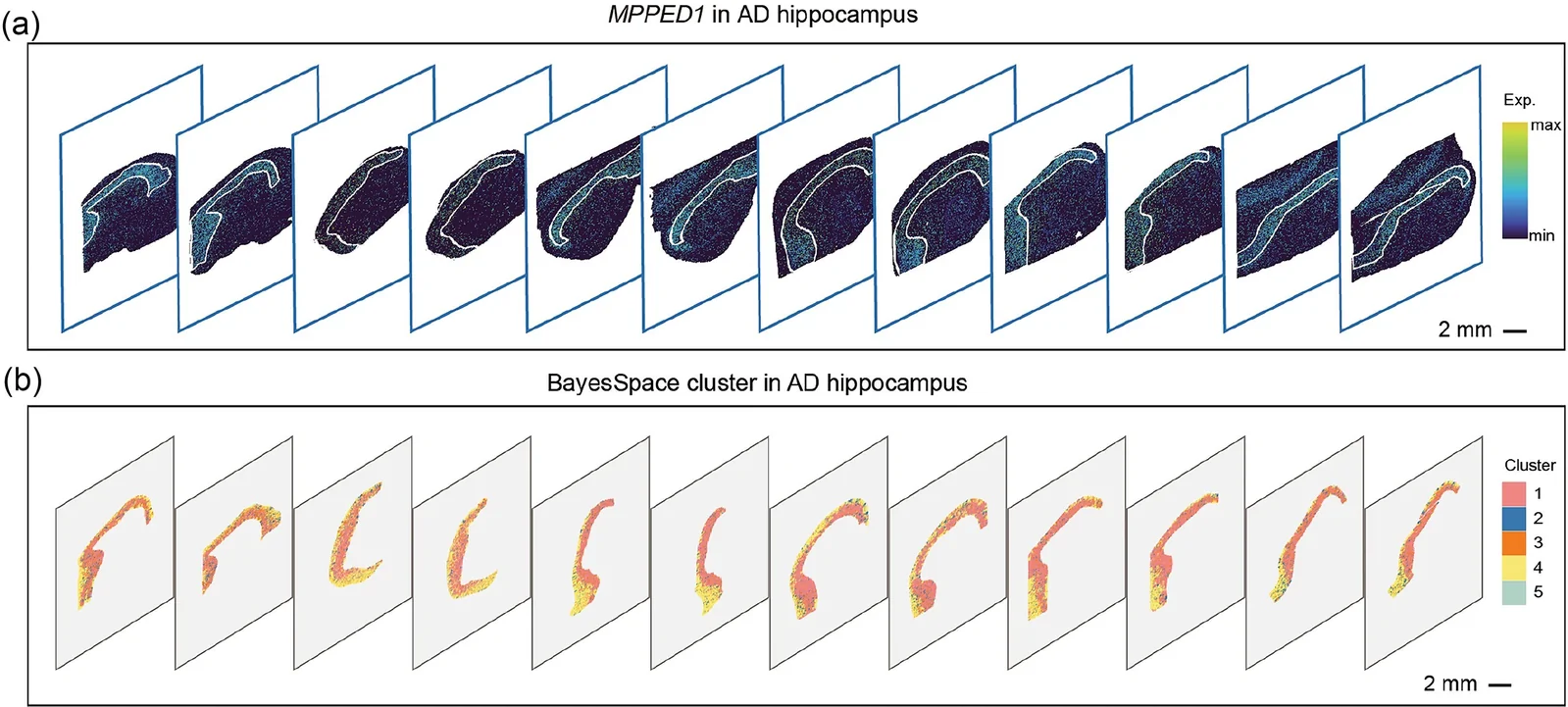
Region parcellation of human hippocampus of AD samples by gene expression pattern. (a) The expression of *MPPED1* gene in hippocampal CA1 brain region in AD patients. (b) The gene expression of internal heterogeneity in the AD CA1 regions based on BayesSpace algorithm. Scale bar: 2 mm.

### Molecularly anchoring the Sub–CA1 boundary

Given the ambiguous definition of the Sub–CA1 boundary in conventional maps [16, 17], we hypothesized that one of the 2 transcriptomic domains corresponded to the Sub. Based on its spatial position, BayesSpace Cluster 4 was the more likely candidate: it localized to the peripheral edge of the putative CA1 territory, consistent with the expected position of Sub. To validate this assignment, we integrated our spatial data with single-nucleus RNA-seq profiles from 6 human hippocampi [21]. Joint clustering of CA1 and Sub cells identified 3 distinct Sub neuron subtypes (distal Sub, proximal Sub marked by *COL5A2*, and proximal Sub marked by *SEMA3E*) (Fig. 2a and b, Extended Data Fig. 2a and b). We next screened established hippocampal cell-type markers to find those enriched in Sub. Strikingly, only *FN1* showed pronounced enrichment in a subset of Sub neurons [21, 22] (Fig. 2c and d, Extended Data Fig. 2c–f), whereas other candidates either marked different regions (e.g., *ROBO1* labeled CA2–4; Extended Data Fig. 2c) or lacked specificity (*COL5A2, NTNG1* and *SEMA3E* showed some Sub enrichment but also signal in CA1 or CA2–4; Extended Data Fig. 2c–e). Crucially, in our spatial atlas *FN1* expression was almost entirely confined to Cluster 4 and absent from the surrounding CA1 (Fig. 2e, Supplementary Table 3). This one-to-one correspondence unequivocally identified Cluster 4 as the Sub, allowing us to use *FN1* as a molecular boundary marker and draw a transcriptionally clear, previously unresolved boundary between CA1 and Sub for the first time (Fig. 2f, g; Extended Data Fig. 2g, h).

**Figure 2:**
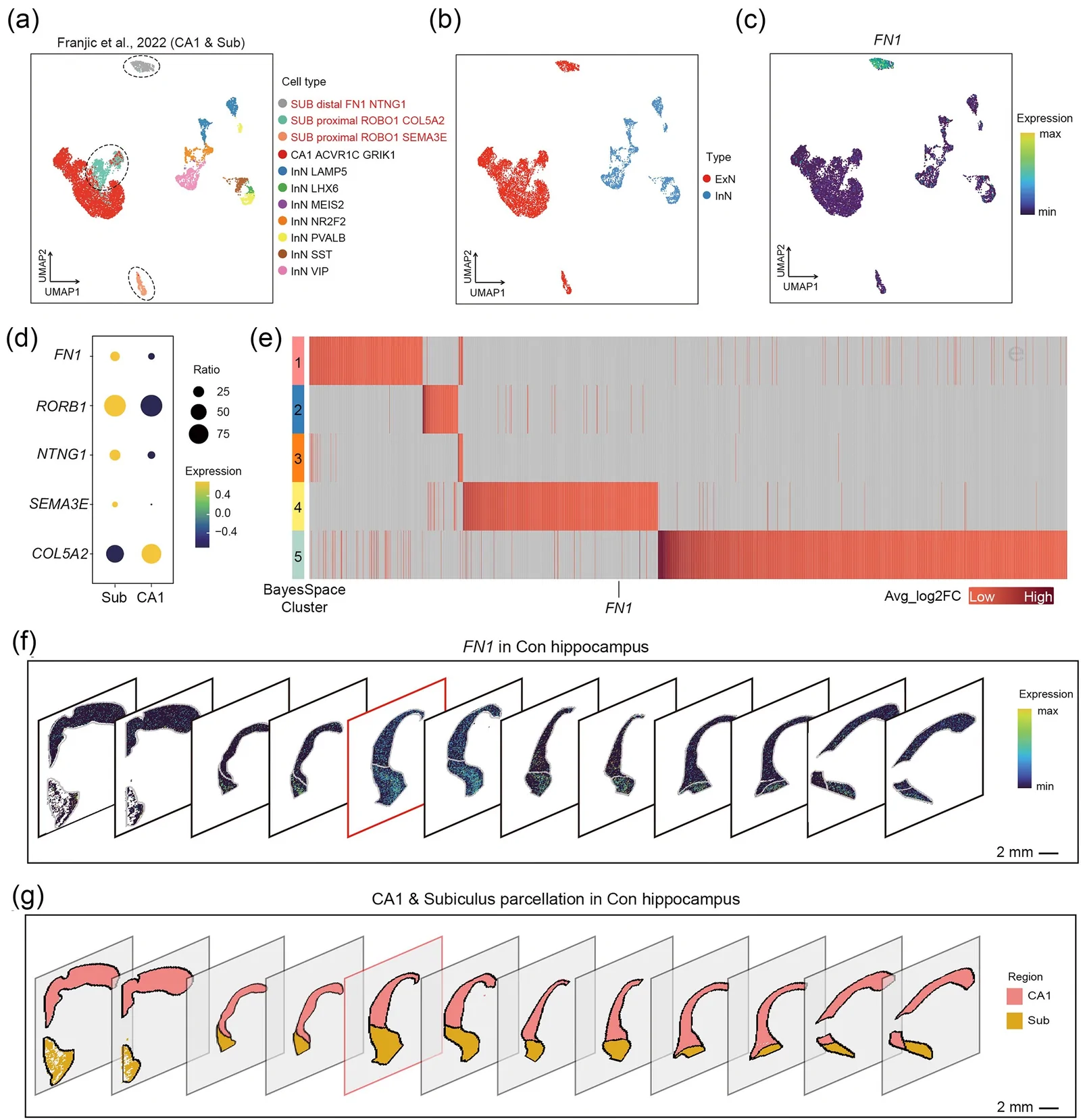
Identification and screening of marker genes in Sub region using public data. (a–b) UMAP visualization of snRNA dataset from the human hippocampus colored by cell types. (a) and neuron types (b). The UMAP was obtained by extracting cells from the CA1 and Sub brain regions. (c) Expression pattern of *FN1* in the UMAP of CA1 and Sub regions. (d) Dot plot showing the expression of reported region-specific genes cross brain regions. (e) The heatmap showing the differential expression genes of each cluster in BayesSpace. (f) Spatial expression of *FN1* in the Con hippocampal regions. (g) Parcellation of the Sub and CA1 regions in the Con hippocampal regions. Scale bar: 2 mm.

**Extended Data Figure 2:**
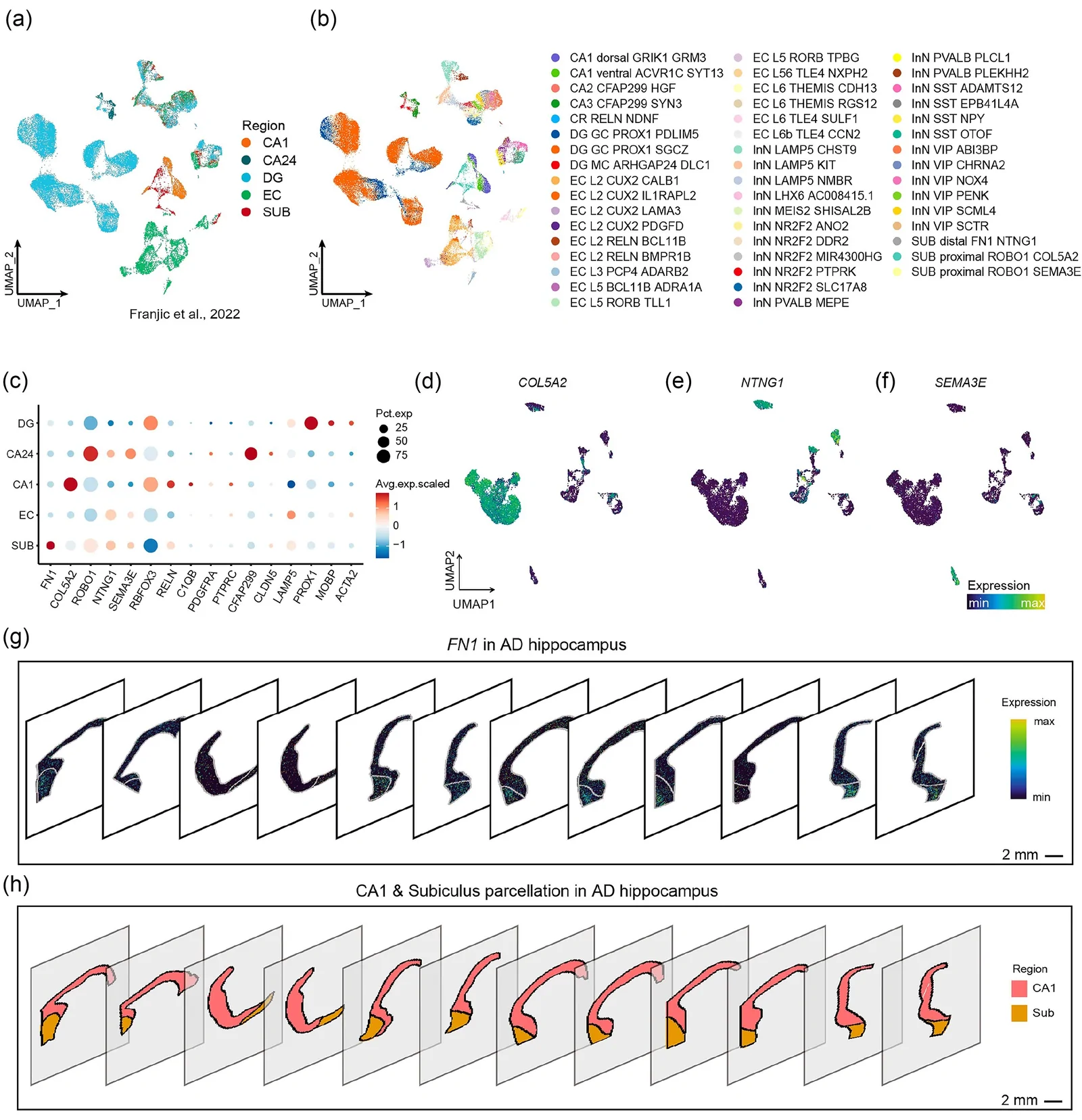
Expression of non-signature genes in public data and confirmation of hippocampal regions. (a–b) UMAP visualization of snRNA dataset from the human hippocampus colored by regions (a) and cell types (b) [21]. (c) Dot plot showing the expression of reported region-specific genes cross brain regions. (d–f) Expression of *COL5A2* (d), *NTNG1* (e), and *SEMA3E* (f) genes in the UMAP of CA1 and Sub regions. (g) Spatial expression of *FN1* gene in the AD hippocampal regions. (h) Parcellation of the Sub and CA1 regions in the AD hippocampal regions.

### Characterization and cross-platform benchmarking of the sub-specific molecular signature

After establishing the spatial demarcation of the Sub, we sought to define the definitive molecular signature that distinguishes this architecture from the adjacent CA1. Differential expression analysis revealed a suite of genes with highly enriched expression in Sub versus CA1 (Fig. 3a; Supplementary Table 4). Among these, we focused on 3 top markers: ETS variant transcription factor 1 (*ETV1*), proprotein convertase subtilisin/kexin type 1 (*PCSK1*), and calcium/calmodulin-dependent protein kinase II gamma (*CAMK2G*), which were robustly upregulated in the Sub.

**Figure 3:**
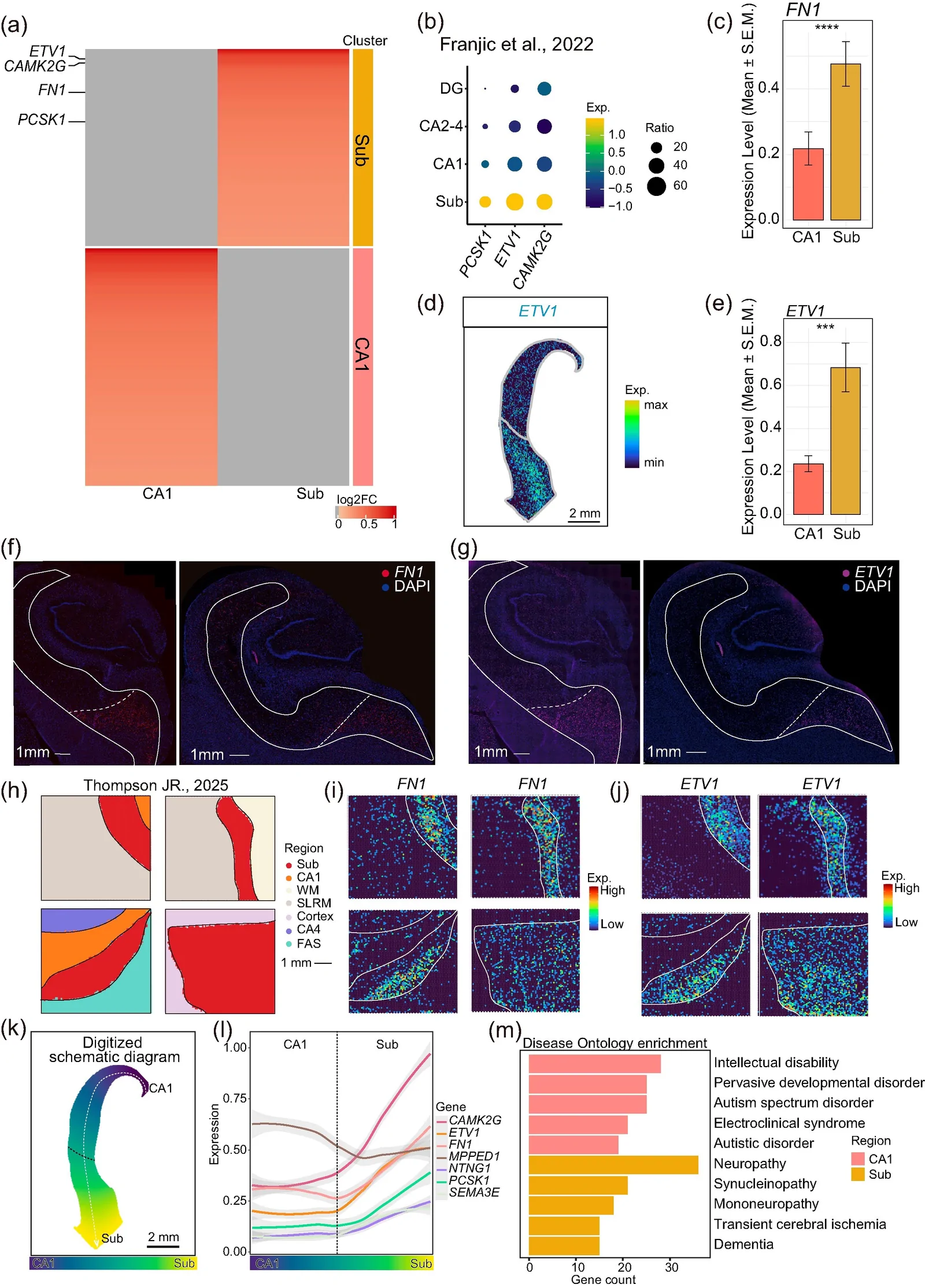
Analysis of differences between Sub and CA1 regions. (a) Heatmap showing average log_2_ (fold change [FC]) of region-specific expressing genes for each area. (b) Dot plot showing the marker genes of Sub region in public snRNA dataset. (c) Quantitative statistics of *FN1* gene in hippocampal regions. Difference between CA1 and Sub was examined by *t*-test with Holm’s correction, *** *P* < 0.001. (d) Spatial expression of *ETV1* gene in hippocampal regions of Con individual. (e) Quantitative statistics of *ETV1* gene in hippocampal regions of Con individuals. Difference between CA1 and Sub was examined by *t*-test with Holm’s correction, *** *P* < 0.001. (f) RNAscope validation of FN1 transcript signals and nuclear counterstaining in Con (left) and AD (right) chips. (g) RNAscope validation of ETV1 transcript signals and nuclear counterstaining in Con (left) and AD (right) chips. (h) Area annotation of public spatial data from Thompson et al. [26]. (i) The expression of *FN1* in public spatial data. (j) The expression of *ETV1* in public spatial data. (k) The digitized schematic diagram of CA1 and Sub brain regions. (l) The expression of marker genes statistically analyzed after digital stratification. (m) Disease ontology (DO) terms analysis was enriched by Sub/CA1-specific genes.

We verified their regional specificity in the public snRNA-seq dataset [21], confirming that each is predominantly expressed by Sub neurons (Fig. 3b). *ETV1*, a transcription factor linked to synaptic plasticity, showed a patchy distribution corresponding to subicular subdomains [23]; *PCSK1*, involved in neuropeptide processing [24], and *CAMK2G*, a kinase regulating calcium signaling [25], were likewise concentrated in Sub. We further quantified *FN1* expression in spatial data, which confirmed *FN1*’s preferential enrichment in Sub compared to CA1 (Fig. 3c). Similarly, spatial expression maps and spot-wise quantification for *ETV1, PCSK1*, and *CAMK2G* showed graded enrichment across the CA1–Sub transition zone and within Sub, reinforcing their specificity (Fig. 3d, e; Extended Data Fig. 3a–e).

**Extended Data Figure 3:**
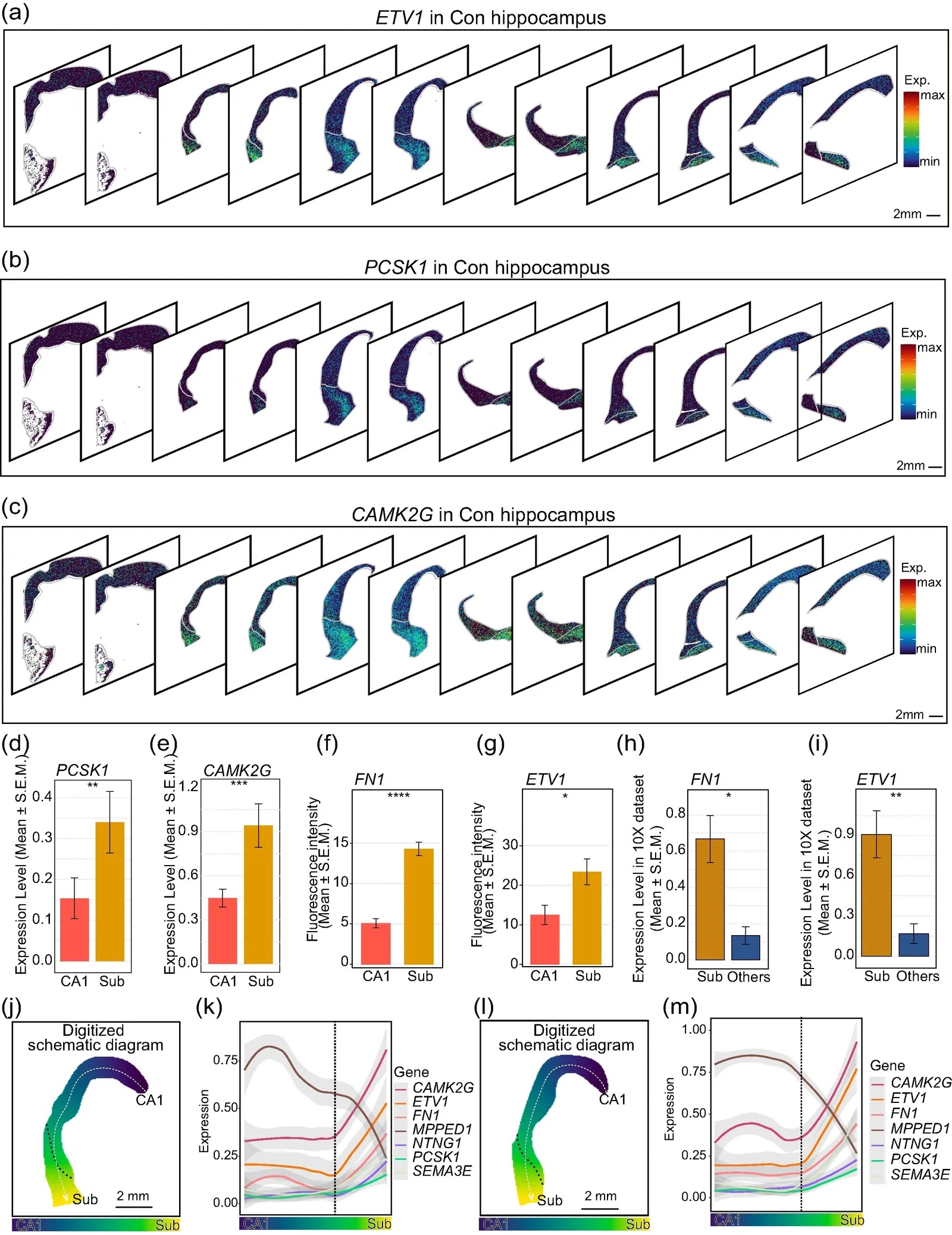
Spatial visualization of *ETV1, PCSK1* and *CAMK2G* genes in all samples (a–c) Spatial expression of *ETV1* (a), *PCSK1* (b), and *CAMK2G* (c) genes in the Con hippocampal regions. Scale bar: 2 mm. (d–e) Quantitative statistics of *PCSK1* (d) and *CAMK2G* (e) in hippocampal regions of Con individuals. Difference between CA1 and Sub was examined by *t*-test with Holm’s correction, ^****^  *P* < 0.0001, ** *P* < 0.01. (f–g) Background-corrected RNAscope fluorescence intensity was quantified for *FN1* (f) and *ETV1* (g) in CA1 and Sub regions by randomly sampling 10 subareas per region in each section. (h–i) Quantitative statistics of *FN1* (h) and *ETV1* (i) expression across hippocampal regions in the 10X dataset [26]. (j and l) The digitized schematic diagram of CA1 and Sub regions. (k and m) The expression of specific genes in the CA1 and Sub brain regions after digitalization (j and l).

To provide orthogonal validation, we performed RNAscope *in situ* hybridization for *FN1* and *ETV1* using in human hippocampal sections. Both transcripts showed enriched localization in the Sub region, consistent with the spatial patterns identified by Stereo-seq (Fig. 3f, g; Extended Data Fig. 3f, g). As an additional cross platform assessment, we analyzed a recently published 10X Genomics spatial transcriptomics dataset of human hippocampus [26]. Although the 10X chips had smaller capture areas and did not fully encompass the CA1–Sub continuum, we identified sections containing both regions (Fig. 3h). In these sections, *FN1* and *ETV1* showed preferential expression in the Sub, with limited expression in adjacent regions including the CA1 (Fig. 3i and j; Extended Data Fig. 3h and i), which provides cross-platform support for the regional enrichment patterns of these representative Sub markers. To further assess whether these regional expression were conserved across species, we examined publicly available *in situ* hybridization data from the Allen Mouse Brain Atlas [27] together with a recently published mouse brain spatial transcriptomic dataset [28]. In the Allen Mouse Brain Atlas, *Fn1* and *Etv1* showed prominent enrichment in the anatomically defined Sub region across multiple mouse brain sections and independent image series (Extended Data Fig. 4a–b). Consistently, analysis of the hippocampal regions extracted from 3 mouse spatial transcriptomic sections from the published dataset also revealed higher expression of both genes in the Sub (Extended Data Fig. 4c–e). The concordant patterns observed across 2 independent anatomical and spatial transcriptomic resources provide cross-species support for the preferential expression of *Fn1* and *Etv1* with the Sub region and further support the Sub-enriched expression patterns identified in the human spatial transcriptomic dataset.

Moreover, by digitally partitioning multiple control hippocampi into CA1 versus Sub regions, we examined expression gradients of key genes across the boundary (Fig. 3k, Extended Data Fig. 3j and l). We found that Sub-specific genes, especially *FN1* and *ETV1*, showed low expression in CA1, increased across the CA1–Sub transition zone, with levels increasing deeper into Sub. In contrast, *MPPED1*, a CA1-enriched but not CA1-exclusive marker, showed the opposite trend: high in proximal CA1 and tapering off across the boundary into Sub. Interestingly, *NTNG1* and *SEMA3E* also increased gradually along the Sub, suggesting they may serve as markers for proximal-distal subicular sublayers (Fig. 3l, Extended Data Fig. 3k and m). Finally, to explore functional distinctions, we performed disease-relevance enrichment using genes enriched in each operationally defined region (focusing on control samples to avoid AD-related bias). The CA1 signature showed overlap with pathways associated with intellectual disability, whereas the Sub signature was associated with synucleinopathies and dementia (Fig. 3m). These descriptive associations indicate that CA1 and the Sub possess regionally distinct molecular profiles. While further study is required to determine if these signatures relate to specific disease vulnerabilities, this underscores our framework’s utility in enabling molecularly informed, region-resolved interrogation of brain disease.

### Application case study: high-resolution parcellation reveals region-specific transcriptional remodeling in AD

To illustrate the utility of our molecularly anchored framework for resolving region-associated transcriptional changes (Fig. 3), we examined AD-associated expression patterns across the defined CA1–Sub interface. Applying the molecular parcellation framework, we identified 467 AD-associated differentially expressed genes (DEGs), comprising 278 upregulated and 189 downregulated genes (Supplementary Table 5). These DEGs showed distinct region-dependent transcriptional enrichment patterns, illustrating the spatial heterogeneity of AD-associated transcriptional changes across the CA1–Sub interface.

Among upregulated DEGs, 27 genes were shared between Sub and CA1 and were enriched for processes related to metal ion homeostasis and regulation of apoptosis. Region-biased signals were also evident: CA1-upregulated DEGs (*n* = 122) were enriched for hypoxia response and gliogenesis-related terms, compatible with glial- and stress-associated transcriptional changes. Sub-upregulated DEGs (*n* = 129) were enriched for gene sets annotated to ATP metabolic processes and electron transport-coupled pathways [29] (Fig. 4a and b; Extended Data Fig. 5a and b). For example, *NDUFA4*, a mitochondria-associated gene involved in respiratory chain function, showed altered expression in Sub in AD, suggesting an AD-associated transcriptional change in oxidative phosphorylation-related pathways in this region [30] (Fig. 4a and b). Because neuronal loss, cellular stress, and other AD-associated changes may influence region-level and cellular-level expression patterns, transcriptional enrichment in metabolic or stress-response pathways does not necessarily indicate corresponding changes in metabolic activity or cellular function.

**Extended Data Figure 4:**
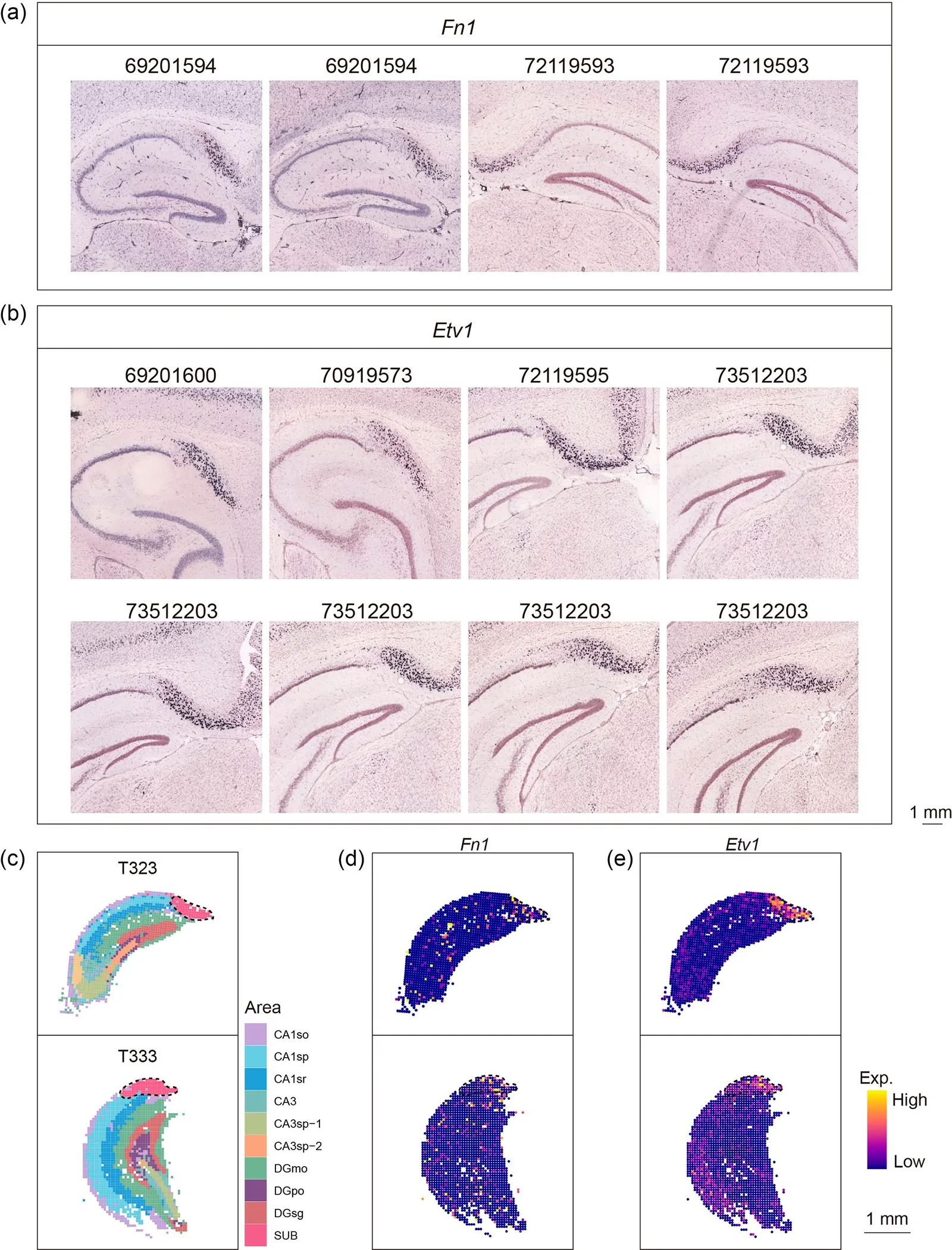
Sub-enriched *Fn1* and *Etv1* expression in the mouse brain. (a) *In situ* hybridization images from the Allen Mouse Brain Atlas showing *Fn1* expression in 4 mouse brain sections, including 2 sections from image series 69201594 and 2 from image series 72119593 [27]. (b) *Etv1* expression in 5 mouse brain sections from image series 69201600, 70919573, 72119595, and 73512203. (c) Anatomical annotations of hippocampal regions in 2 publicly available mouse brain spatial transcriptomic sections generated by Han et al. [28]. (d–e) Spatial expression patterns of *Fn1* and *Etv1*, respectively, in the corresponding hippocampal sections. Both genes showed consistently higher expression in the Sub across the analyzed sections.

**Figure 4:**
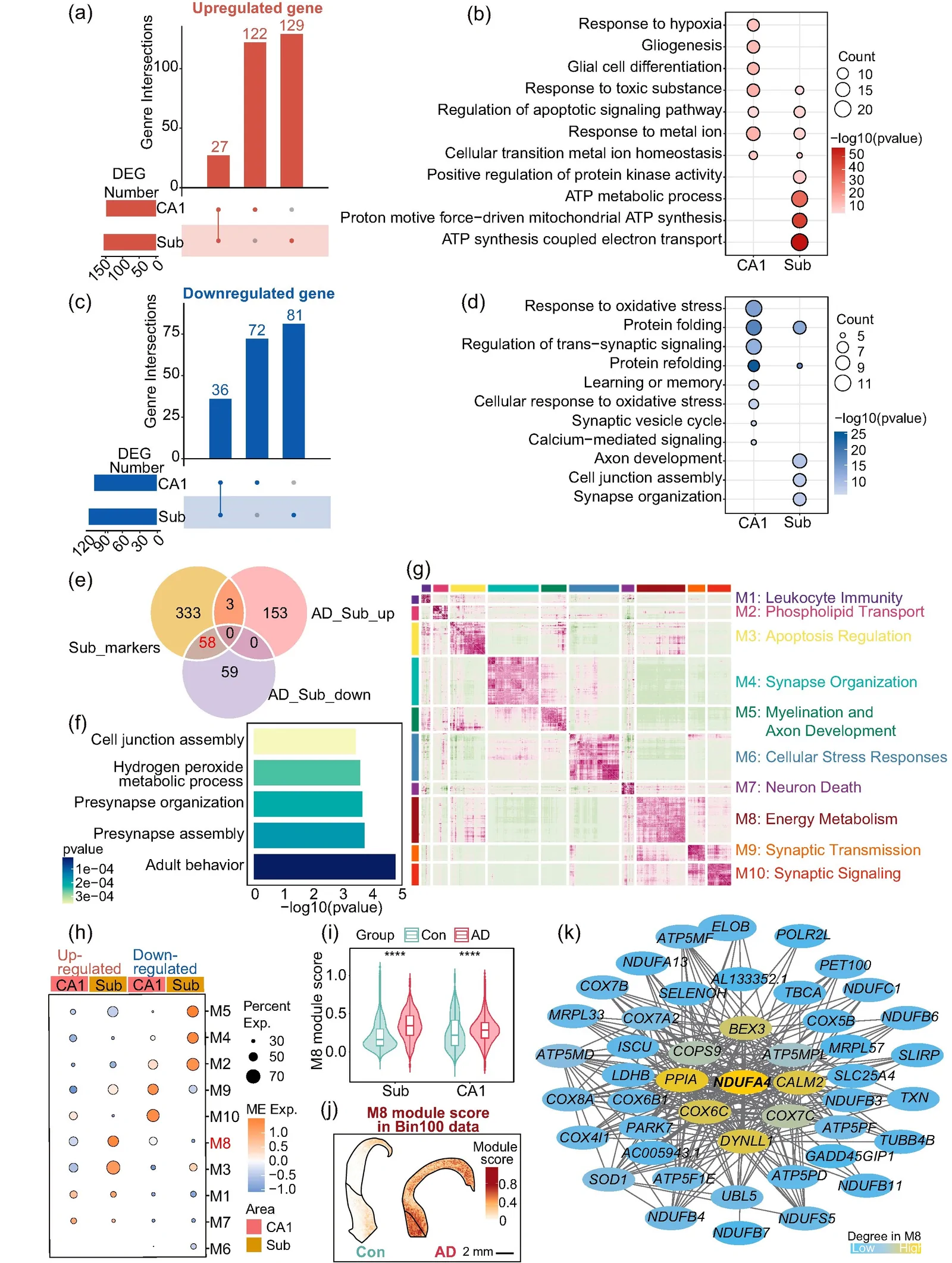
Differential gene expression and co-expression network analysis in Sub and CA1 regions of AD using bin100 data (a and c). The number of upregulated (a) and downregulated (c) DEGs across the Sub and CA1 regions in AD. (b and d) Dot plots showing the enriched GO terms for upregulated (b) and downregulated (d) DEGs in the Sub and CA1 regions, respectively. (e) The intersection of Sub-specific genes and the AD DEGs in the Sub region. (f) Pathway enrichment analysis Sub-specific genes downregulated in AD. (g) Heatmap showing 10 modules of DEGs classified by hdWGCNA, with each correlated to distinct biological processes. (h) Dot plot showing the Module eigengenes (ME) for DEGs in Sub and CA1 regions within AD and Con. (i) Violin plot illustrating the M8 module gene scores between AD and Con within Sub and CA1 regions in all chips. Difference between AD and Con was examined by *t*-test with Holm’s correction, ^****^  *P* < 0.0001. (j) Spatial expression of M8 module gene scores in hippocampal regions of AD (right) and Con (left). Scale bar: 2 mm. (k) Co-expression network of intersection of M8 module genes and upregulated genes in AD Sub region visualized using Cytoscape software. Edges represent interactions, and node color indicates connectivity degree.

Downregulated DEGs in both regions converged on proteostasis-related pathways, while Sub-biased downregulated DEGs showed additional enrichment for synapse organization (Fig. 4c and d). To assess whether AD preferentially perturbs regional identity programs, we intersected region-specific marker sets with AD-related DEGs. In Sub, 58 Sub markers were downregulated in AD, whereas only 3 Sub markers (*SNCG, TUBB2B, RPS27*) were upregulated (Fig. 4e; Supplementary Table 6); the downregulated Sub markers were enriched for synaptic functional terms (Fig. 4f). Notably, *NDUFA4* was not a Sub identity marker, suggesting that AD-related transcriptional remodeling in Sub involves not only attenuation of region-specific programs but also induction of additional stress-associated processes. In CA1, 43 CA1-specific markers were all downregulated in AD (Extended Data Fig. 5c; Supplementary Table 7), with enrichment pointing to protein polymerization-related processes (Extended Data Fig. 5d).

To capture coordinated network-level changes, we performed high-dimensional weighted gene co-expression network analysis (hdWGCNA) using AD-associated DEGs and identified 10 co-expression modules [31] (Fig. 7g; Extended Data Fig. 5e; Supplementary Table 8). Module eigengenes showed region-dependent expression patterns and functional annotations (Fig. 4h; Extended Data Fig. 5f–i). Sub-biased downregulated genes were enriched in modules associated with lipid handling/high-density lipoprotein particle clearance [32], neuronal support function, and regulation of synaptic plasticity [18, 33] (M2, M5, and M4; Fig. 4g; Supplementary Table 9). In contrast, Sub-biased upregulated genes were concentrated in an energy metabolism module (M8), enriched for ATP metabolic processes and electron transport-coupled pathways (Fig. 4g–j; Supplementary Table 9). Network topology further highlighted mitochondria-related hub genes, including *NDUFA4* and *CALM2* [30, 34] (Fig. 4k).

Collectively, these analyses illustrate the utility of our spatial framework for resolving region-associated transcriptional heterogeneity in AD, providing a region-resolved view of AD-associated molecular heterogeneity across the CA1–Sub interface.

### Multi-scale mapping identifies neuronal drivers of sub-specific bioenergetic programs

To dissect the cellular contributors to the regional metabolic signature observed in the Sub, we performed brain region-specific alignment by mapping bin100 matrices to cellbin coordinates, yielding a spatially resolved single-cell transcriptomic atlas (Fig. 5a; Extended Data Fig. 6a). The accuracy of our cell-type annotations, performed via the Spatial-ID method [35], was corroborated by high similarity with public snRNA-seq benchmarks (Fig. 5b) and further supported by canonical marker expression and rigorous chip-level quality control across both control and AD cohorts (Extended Data Fig. 6).

**Extended Data Figure 5:**
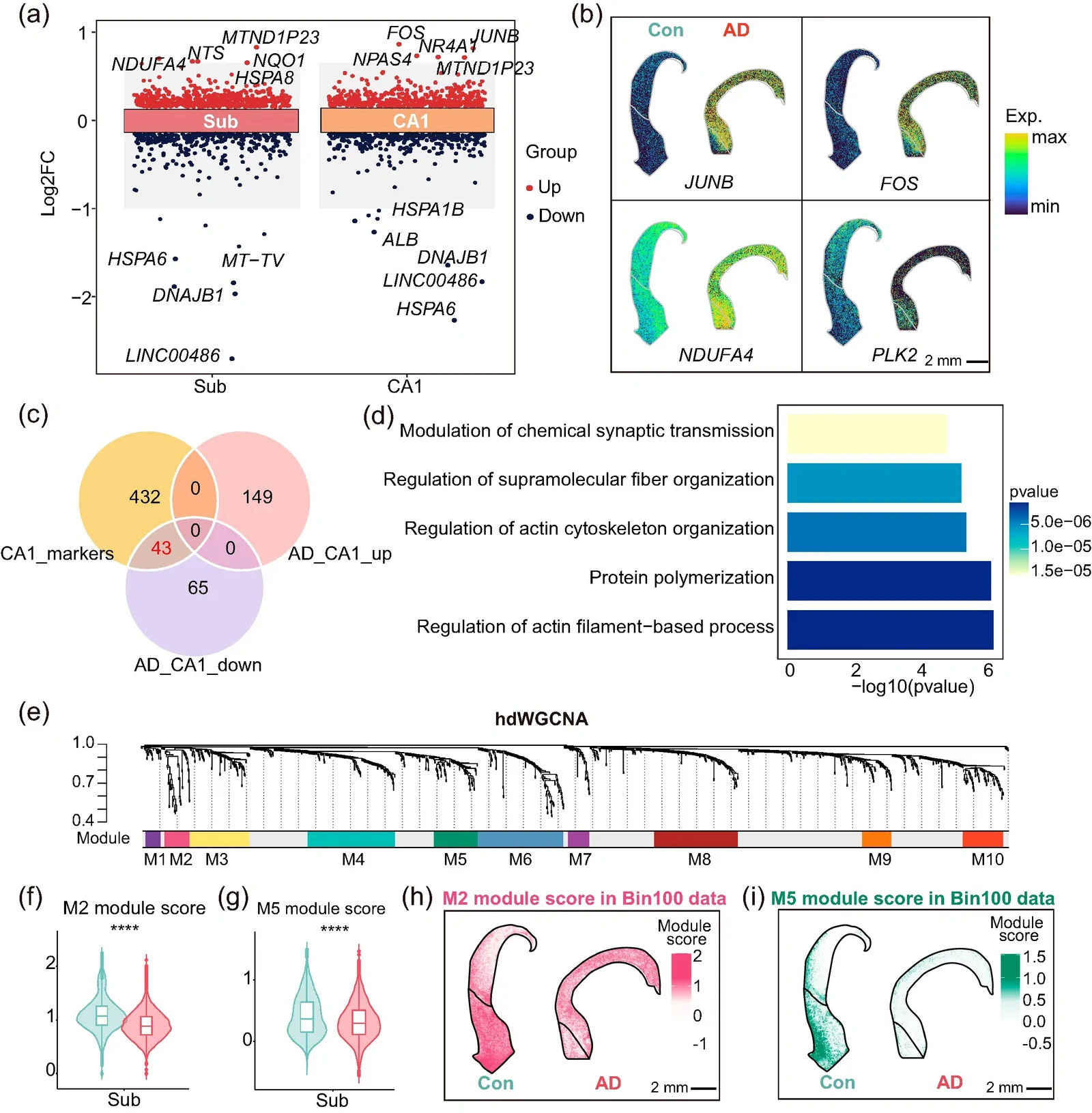
Spatial analysis of AD-linked DEGs and modules in Sub and CA1 regions (a). The volcano plots showing the AD-associated DEGs in the Sub and CA1 regions. (b) Spatial expression of *JUNB, PLK2, NDUFA4, CALM2* genes between AD and Con groups. (c) The intersection of Sub-specific genes and the AD DEGs in the Sub region. (d) Pathway enrichment analysis CA1-specific genes downregulated in AD. (e) Ten co-expression modules of DEGs clustered by hdWGCNA. (f–g) Violin plots illustrating the M2 and M5 module gene scores between AD and Con within Sub and CA1 regions. Difference between AD and Con was examined by *t*-test with Holm’s correction, ^****^  *P* < 0.0001. (h–i) Spatial distribution of M2 and M5 module gene scores of Con (left) and AD (right). Scale bar: 2 mm.

**Figure 5:**
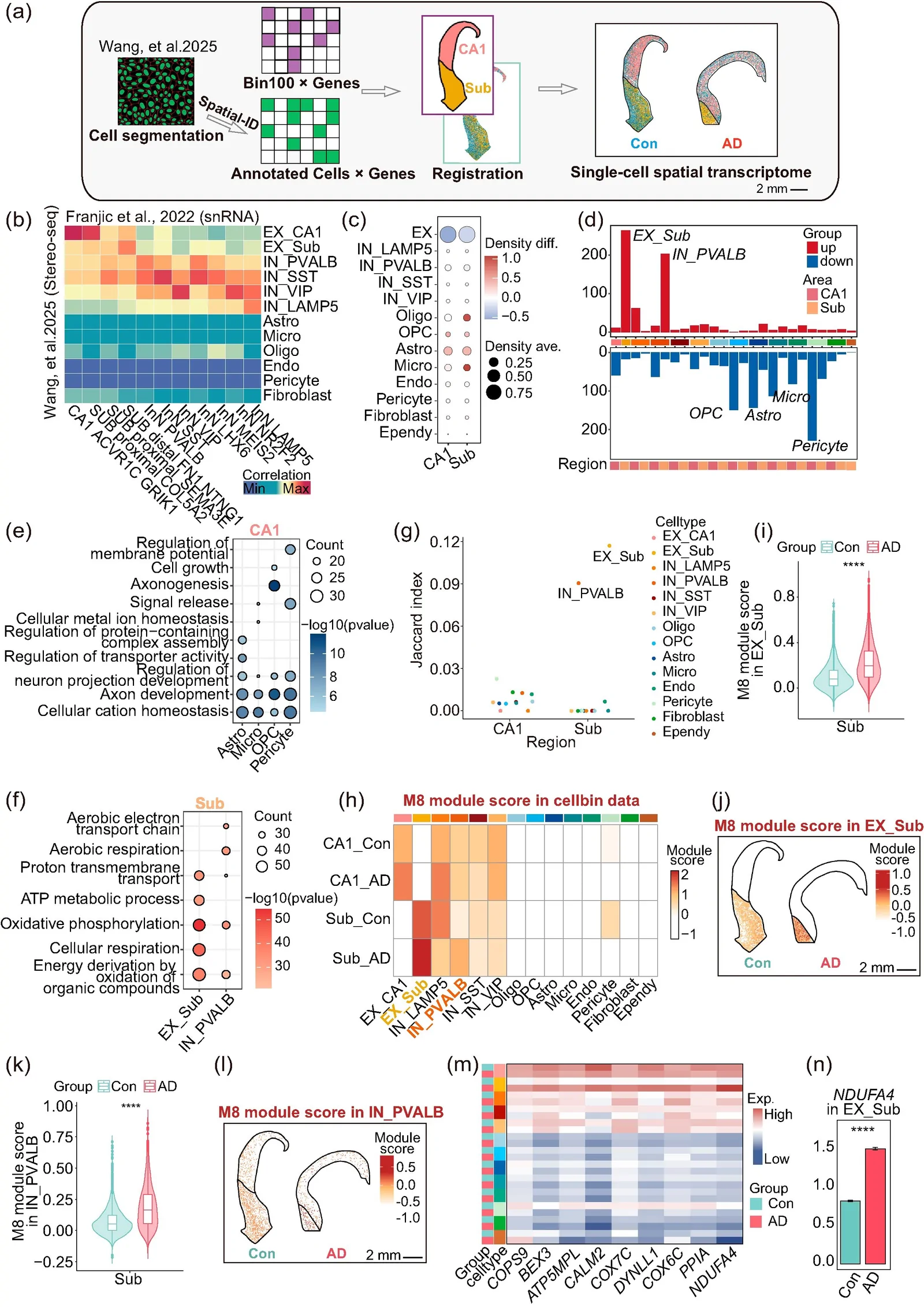
Single-cell spatial transcriptome analysis revealed cell type-specific gene expression changes in the Sub and CA1 regions of AD (a). The workflow illustrating the integration of bin100 and cellbin matrices to generate spatially resolved single-cell transcriptomic dataset with brain region annotations. (b) Pearson correlation of cell types between snRNA dataset from Franjic et al. [21] and our stereo-cell data. (c) Dot plot depicting cell type densities in the Sub and CA1 regions. The spot size represents the average density of each cell type for AD and Con. The spot fill indicates the direction of cell-density change in AD relative to Con. Density diff., density difference. (d) Bar plot quantifying upregulated and downregulated DEGs across cell types in the AD group. (e) Dot plot showing the enriched GO terms for downregulated DEGs of Astro, Micro, OPC, and Pericyte in the CA1 region. (f) Dot plot showing the enriched GO terms for upregulated DEGs of EX_Sub and IN_PVALB neurons in the Sub region. (g) Dot plot showing the Jaccard similarity index of M8 module genes and DEGs of different cell types. (h) Heatmap of M8 module score across Sub and CA1 regions within the different cell types in AD and Con groups. (i and k) Violin plots showed the M8 module gene scores between AD and Con for EX_Sub (i) and IN_PVALB (k) cell types within the Sub region across all chips. Difference between AD and Con was examined by *t*-test with Holm’s correction, ^****^  *P* < 0.0001. (j and l) Spatial expression of M8 module gene scores in EX_Sub (j) and IN_PVALB neurons (l) of AD (right) and Con (left). Scale bar: 2 mm. (m) Heatmap of expression for hub genes (*COPS9, BEX3, ATP5MPL, CALM2, COX7C, DYNLL1, COX6C, PPIA, NDUFA4* genes) in the Sub region of different cell types. (n) Bar plot showing mean expression of *NDUFA4* in EX_Sub neurons of AD and Con groups. Difference between AD and Con was examined by *t*-test with Holm’s correction, ^****^  *P* < 0.0001.

**Extended Data Figure 6:**
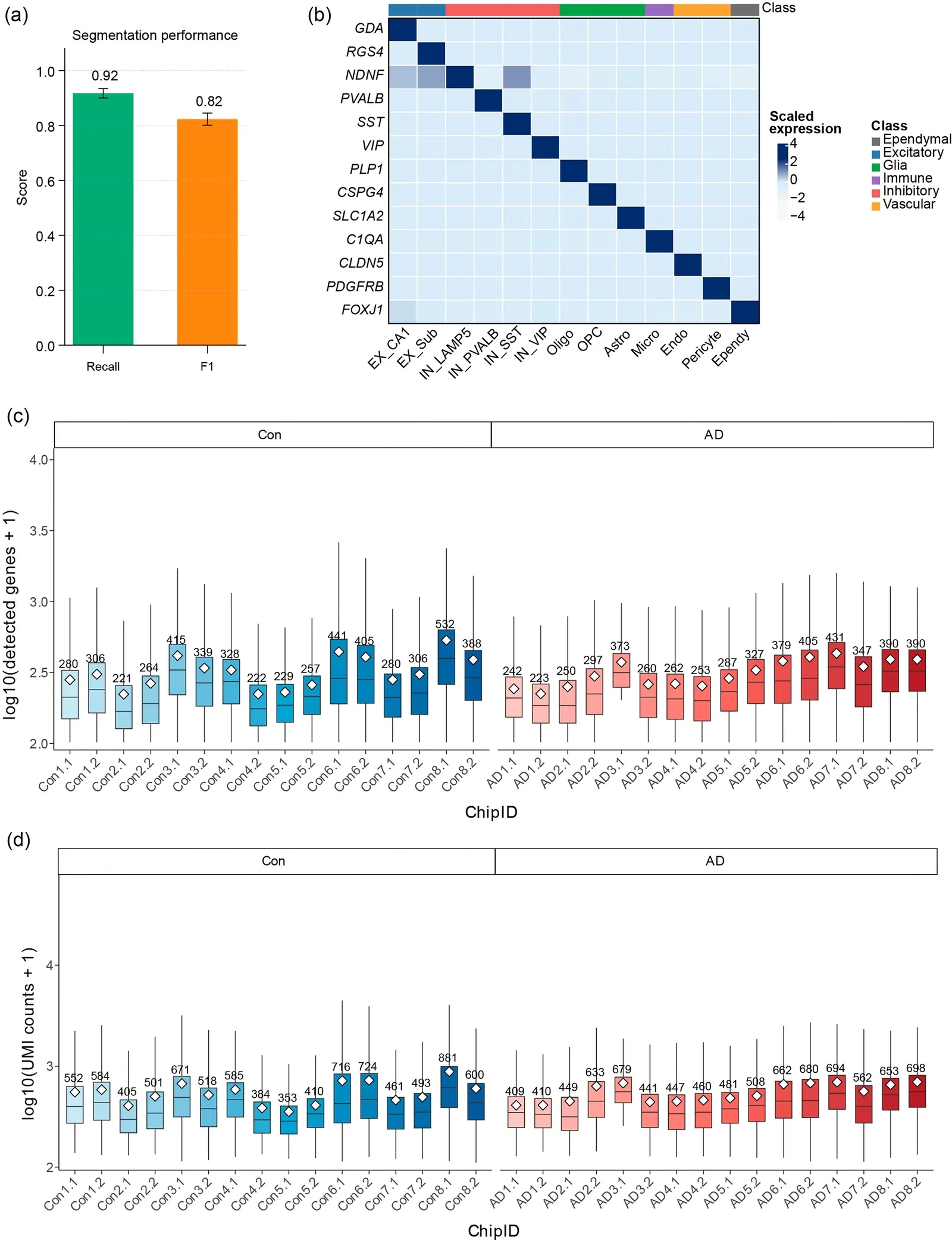
Technical quality control and validation of cell segmentation, cell-type annotation, and spatial transcriptomics. (a) Benchmark of cell segmentation accuracy via manual inspection across 3 representative, high-magnification image fields. (b) Heatmap displaying the relative expression levels of canonical marker genes across the annotated major cell classes, confirming the specificity and high fidelity of cell-type assignments. (c–d) Chip-level quality-control assessments across control and AD post-mortem human hippocampal samples, showing the spatial distribution and density of detected gene counts (c) and UMI counts (d) per spatial bin.

Quantitative density analysis revealed a significant reduction in neuronal populations, particularly excitatory neurons, alongside an increased microglial density in both the Sub and CA1 regions of AD patients (Fig. 5c). In addition, cell-type-specific DEGs analysis showed different expression patterns between Sub and CA1 regions. In the CA1 region, the DEGs across multiple cell types, such as Astro, Micro, OPC, and pericyte, were enriched for pathways related to cellular cation homeostasis and axonal development (Fig. 5d, f). In contrast, in the Sub region, DEGs were predominantly upregulated in specific neuronal populations, most notably EX_Sub and IN_PVALB neurons, and were enriched for mitochondrial energy metabolism pathways, including ATP metabolic processes, aerobic respiration, and oxidative phosphorylation (Fig. 5d, f).

To connect these cell-type signatures to our region-level co-expression network, we compared cell-type-specific DEGs with the hdWGCNA modules using Jaccard similarity. This analysis highlighted a strong overlap between the mitochondrial/energy module M8 and DEGs from EX_Sub and IN_PVALB neurons (Fig. 5g). Consistently, gene set scoring showed significant elevation of M8-associated expression programs in EX_Sub and IN_PVALB neurons in the AD Sub (Fig. 5h–l), with increased M8 scores in AD versus controls for both neuronal populations (Fig. 5i, k) and spatial visualization recapitulating these shifts at the tissue level (Fig. 5j, l). Finally, analysis of M8 hub genes identified *NDUFA4* as one of the most strongly upregulated electron transport chain-associated genes in AD EX_Sub neurons (Fig. 5m, n).

Collectively, these results demonstrate that the Sub-specific metabolic signature in AD is not a global tissue response but is preferentially engaged within specialized neuronal populations, a discovery enabled by our high-resolution parcellation framework.

## Discussion

Accurate identification of CA1–Sub boundary is essential for human brain studies using limited surgical or post-mortem tissue. Our study establishes spatially resolved molecular framework specifically designed to delineate the human CA1–Sub interface. By performing a secondary analysis of a previously published human hippocampal Stereo-seq dataset and integrating the spatial profiles with snRNA-seq reference data, we identified coordinated molecular signatures that consistently distinguished CA1 and Sub across the analyzed donors. *FN1* enrichment in a subset of EX_Sub, together with convergent patterns of *ETV1, PCSK1, CAMK2G*, and other regional markers, supported reproducible assignment of CA1 and Sub identities. The regional association of *FN1* and *ETV1* with the Sub was further supported by 3 independent brain resources [26–28]. To our knowledge, this represents the first molecularly anchored, cross-platform validated coordinate system for the human CA1–Sub interface, reframing a problem historically treated as a matter of anatomical judgment into one that can be resolved computationally and reproducibly from gene expression data.

It is important to clarify the nature of the CA1–Sub boundary identified in this study. Traditionally, the CA1–Sub interface has been delineated primarily according to cytoarchitectural features, including changes in pyramidal-cell density, layer thickness, cellular morphology, and laminar organization [36, 37]. However, the precise placement of this boundary remains challenging because these features can vary gradually across the interface and may differ across individuals and along the rostrocaudal axis [38]. More recent single-cell and spatial transcriptomic studies have further shown that the Sub is molecularly heterogeneous, comprising spatially organized neuronal populations distributed along its proximal–distal, superficial–deep, and longitudinal axes rather than forming a molecularly uniform compartment [21, 39, 40].

Our findings reconcile these apparently discrete and continuous features of subicular organization. At the multigene level, Bayesian spatial inference identified a reproducible and visually distinct interface between these domains across tissue sections [20]. Thus, although the CA1–Sub boundary has traditionally been difficult to define consistently using cytoarchitectural criteria alone, the combined expression of multiple regional markers provided a clearer and more reproducible distinction [38]. In contrast, examination of individual genes revealed a more gradual biological transition. CA1-enriched *MPPED1* decreased toward the Sub, whereas *FN1, ETV1, PCSK1*, and *CAMK2G* increased across the CA1–Sub interface. *NTNG1* and *SEMA3E* showed additional progressive variation along the proximal–distal axis of the Sub. Even *FN1* and *ETV1*, despite their strong regional enrichment, did not exhibit an abrupt step-function change at a single anatomical line. We therefore interpret the CA1–Sub boundary not as an absolute discontinuity in the expression of individual genes, but as a probabilistically defined transition between 2 distinguishable multivariate molecular states. This interpretation is consistent with previous transcriptomic studies demonstrating both discrete, spatially segregated EX_Sub subclasses and continuous molecular organization within the subicular complex. The principal advance of our framework is that it moves beyond subjective cytoarchitectural assessment or reliance on a single marker by integrating spatial information, multigene expression patterns, unsupervised clustering, and Bayesian boundary inference to produce a reproducible, biologically informed operational parcellation in the human hippocampus. In this framework, the boundary is evident at the level of the integrated multigene molecular pattern, even though individual marker genes may vary gradually across the interface. This approach therefore enables more consistent CA1 and Sub annotation and region-resolved comparison across donors and datasets, while preserving the underlying biological continuum of the CA1–Sub transition.

This advance has immediate practical consequences for the field. A long-standing limitation in human hippocampal subfield research is the “boundary-spanning” dilemma: because the CA1–Sub transition cannot be reliably assigned using conventional landmarks, tissue spanning this interface is frequently excluded from analysis to avoid misclassification, resulting in substantial underuse of scarce post-mortem specimens [41, 42]. Our molecular reference resolves this dilemma by enabling principled, gene expression-based assignment of transition-zone tissue, thereby increasing the analyzable yield from each donor and improving cross-laboratory reproducibility [43]. More broadly, this framework offers a template that could, in principle, be extended to other architecturally ambiguous, continuum-like boundaries within the hippocampal formation and adjacent cortical regions, where gradual transitions, rather than sharp anatomical borders, are the rule rather than the exception.

Beyond this practical gain in tissue utilization, we believe the more fundamental impact of this work lies in a methodological shift it enables for the field. Region definition in human hippocampal subfield research has traditionally depended on expert-driven, qualitative interpretation of cytoarchitecture, a process that is inherently difficult to standardize across laboratories, donors, and even between sections from the same specimen. By demonstrating that a small set of molecular markers can reproducibly recover a biologically meaningful transition zone across independent platforms and cohorts, our framework moves CA1–Sub regional assignment from a matter of anatomical expertise toward a quantitative, data-driven, and externally verifiable procedure. This shift has direct downstream consequences: it allows region-resolved comparisons (such as the AD analyses presented here) to be benchmarked against an explicit molecular criterion rather than an implicit and potentially inconsistent anatomical judgment, thereby improving the comparability of findings not only within a single study but, in principle, across future studies that adopt the same molecular reference.

To contextualize this contribution within the existing literature, prior attempts to characterize the CA1–Sub continuum in human tissue have largely relied on either single-marker immunohistochemistry or bulk-tissue dissection guided by visual anatomical estimation [41, 44], both of which carry inherent risk of regional cross-contamination and limited reproducibility across laboratories and donors. Rodent studies have provided higher-resolution insights into Sub molecular identity, but cross-species differences in hippocampal organization limit the direct translation of these findings to human tissue. We therefore used the mouse data as independent anatomical support rather than as the basis for defining the human CA1–Sub boundary. Specifically, both *in situ* hybridization data from the Allen Mouse Brain Atlas and 3 independent mouse brain spatial transcriptomic sections from our previous study [28] showed preferential expression of *Fn1* and *Etv1* in the Sub, indicating that the regional enrichment of these markers is detectable across species and technical platforms. Nevertheless, this cross-species concordance does not imply that the precise cellular distribution or geometry of the CA1–Sub transition is identical between mouse and human. Our broader validation strategy, integrating human Stereo-seq, snRNA-seq, RNAscope, an independent 10X Genomics dataset, and complementary mouse *in situ* hybridization and spatial transcriptomic resources, establishes convergent evidence across independent cohorts, species, and experimental platforms. Together, these results increase confidence that the *FN1*- and *ETV1*-associated Sub-enriched patterns observed in the human data reflect reproducible anatomical organization rather than a platform-specific or donor-specific artifact.

Building on this molecular reference, we performed region-resolved comparisons in AD as a proof-of-concept application. These analyses revealed divergent transcriptional programs across the CA1–Sub interface, including a more prominent induction of mitochondrial energy metabolism-related signatures within the Sub and stress/glial-associated responses in CA1, with the Sub-specific bioenergetic signature further localized to specific neuronal populations (notably EX_Sub and IN_PVALB neurons) at single-cell resolution. We emphasize that these findings are intended to illustrate the discriminating power of our parcellation framework rather than to establish definitive disease mechanisms. Genes such as *NDUFA4*, which emerged as a hub gene within the Sub-enriched energy metabolism module, represent prioritized candidates for future mechanistic investigation into regional vulnerability in AD, rather than validated causal drivers [45]. Further functional studies will be needed to determine whether this bioenergetic remodeling reflects a compensatory response to metabolic stress or a maladaptive process contributing to regional vulnerability.

Several limitations inherent to human brain banking should be acknowledged [46]. First, the use of post-mortem tissue precludes longitudinal assessment of disease progression and may limit sensitivity to molecular changes that precede overt pathology [47]. Among donors with available metadata, we did not detect statistically significant differences between the AD and Con groups in post-mortem interval (PMI) or RNA integrity number (RIN). However, given the cohort size, these results should not be interpreted as formal evidence of equivalence. Moreover, cause of death and agonal-state information was incomplete, and residual confounding related to terminal events and other donor level clinical or technical factors cannot be excluded. Such factors may influence post-mortem gene expression, particularly that of immediate-early and stress responsive genes, such as *FOS, JUNB, NR4A*, and *NPAS4*. Consequently, the observed metabolic and stress-response signatures cannot be fully disentangled from the potential effects of neuronal loss, terminal stress, and other donor-level clinical or technical factors and should therefore be interpreted as exploratory transcriptional associations rather than direct evidence of altered metabolic function. Second, although our cohort of 12 donors, representing all specimens available in the brain bank with matched key covariates, was carefully balanced for age and sex to maximize inter-group comparability, this sample size does not fully capture the broad spectrum of inter-individual variability across AD stages. Specifically, the AD cohort spanned Braak stages III to V, with most cases classified as Braak stage IV, while control cases showed absent to low Braak pathology (not detected to Braak stage II); this pathological heterogeneity within a relatively small AD cohort may contribute to inter-donor variability in region-resolved transcriptional signatures and should be considered when interpreting AD-associated findings. Other donor-level variables, including APOE genotype, may further influence individual variability but could not be fully assessed given the limited sample size. These constraints reflect the inherent unpredictability of donor availability and the strict tissue-integrity requirements of spatial transcriptomics [48], rather than a limitation of study design. Third, the co-expression modules and hub genes identified here, including the M8 mitochondrial module, provide valuable correlative insight but require independent experimental validation to establish causal relationships to AD pathology. Accordingly, the RNAscope and 10X analyses should be interpreted as orthogonal and supportive validation of selected representative markers, rather than as exhaustive validation of all candidate genes identified in this study. Future studies prospectively collecting larger, stage- and genotype-stratified cohorts will be important to validate the generalizability of this molecular reference and to clarify how AD stage, APOE genotype, and other donor-level variables influence CA1–Sub boundary delineation and region-resolved disease signatures.

In conclusion, this study provides a high-resolution molecular atlas and a reproducible, transferable parcellation pipeline for the operationally defined human CA1–Sub interface, addressing a transition that has eluded consistent definition since the earliest anatomical descriptions of the hippocampus. By identifying definitive boundary markers and demonstrating their utility in unmasking region-specific bioenergetic remodeling in AD, our findings provide a foundational resource for reproducible hippocampal subfield research and for region-resolved investigations into neurodegenerative disease.

## Methods

### Dataset acquisition and preprocessing

The sequencing datasets analyzed in this study were obtained entirely from previously published studies. The high-resolution spatial transcriptomic datasets were obtained from the study by Wang et al. [18] and are deposited in the China National GeneBank DataBase (CNSA) under accession number CNP0005077. The present study represented a secondary, boundary-focused reanalysis of these Stereo-seq data, specifically addressing the molecular organization and delineation of the CA1–Sub interface. For this purpose, a broad region of interest encompassing CA1 and the Sub was extracted from the complete hippocampal sections with reference to the spatial expression pattern of *MPPED1* [18]. This region of interest was subsequently used for downstream analyses of spatial gene-expression changes and the CA1–Sub boundary. The complementary snRNA-seq datasets were obtained from Franjic et al. [21] and are publicly available through the Gene Expression Omnibus (GEO) under accession number GSE186538. This dataset contains transcriptomic profiles from multiple micro-dissected hippocampal and parahippocampal regions, including DG, CA2–4, CA1, Sub, and EC, collected from clinically unremarkable adult human donors. This dataset was used to characterize the spatial-specific marker genes related to CA1–Sub interface identification. An independent 10X spatial transcriptomic dataset generated by Thompson et al. [26], available under GEO accession number GSE264624, was used for external cross-platform validation. This previously published dataset was used to determine whether the candidate Sub-enriched markers identified through the Stereo-seq and snRNA-seq analyses showed reproducible spatial enrichment in the Sub on an independent spatial transcriptomic platform. For additional cross-species anatomical validation, publicly available *in situ* hybridization images were obtained from the Allen Mouse Brain Atlas and used to examine the hippocampal expression patterns of *Fn1* and *Etv1* in the adult mouse brain [27]. The analyzed images included independent experiment series for *Fn1* and *Etv1*, as specified in the corresponding figure legend. In addition, 2 publicly available mouse brain spatial transcriptomic sections generated by Han et al. [28] were analyzed to assess the spatial expression patterns of *Fn1* and *Etv1* across anatomically annotated hippocampal regions [28]. These mouse datasets were used as complementary cross-species evidence to evaluate whether the Sub-enriched expression patterns observed in the human datasets were conserved in the mouse hippocampal formation.

### Spatial transcriptomic sample selection

To ensure high data fidelity, the specimens utilized in this study were sourced from an initial pool of 16 hippocampal samples with RIN > 6. From this initial pool, 12 specimens (6 AD and 6 Control) were prioritized for subsequent spatial transcriptomic analysis. Sample selection was based on histological evaluation of tissue architecture and anatomical coverage. Specifically, samples were included if they showed well-preserved tissue morphology, a continuous CA1 region, and the presence of an identifiable and analyzable Sub region, which was required for CA1–Sub-specific downstream analyses. Specimens exhibiting significant tissue fragmentation, freezing artifacts, poor tissue integrity, or incomplete anatomical coverage of the CA1–Sub region were excluded from CA1–Sub-specific analyses. In particular, tissue sections that did not contain an identifiable and analyzable Sub region were not used for downstream analyses requiring CA1–Sub comparison or parcellation. This targeted selection ensured that the analyzed samples provided sufficient structural integrity and spatial coverage for molecularly informed CA1–Sub parcellation. In addition, chips showing marked global transcriptomic heterogeneity relative to the rest of the cohort, as assessed by PCA, were excluded to minimize chip-level outlier effects. This targeted selection ensured that the analyzed samples provided sufficient structural integrity, spatial coverage, and transcriptomic consistency for molecularly informed CA1–Sub parcellation. The inclusion status of each chip is summarized in Supplementary Table 1, where we indicate whether each chip was included in the CA1–Sub analysis and, for excluded chips, provide the corresponding exclusion reason, including absence of an analyzable Sub region or excessive transcriptomic heterogeneity. Detailed donor characteristics, including age, sex, PMI, APOE genotype, clinical diagnosis, and Braak stage, are summarized in Supplementary Table 1. Control donors exhibited absent to low levels of AD neuropathology, ranging from no detectable pathology to Braak stage II, whereas AD donors ranged from Braak stages III to V, with the majority classified as Braak stage IV.

### Stereo-seq raw data processing

The fastq files generated from Stereo-seq were analyzed in accordance with the established pipeline, which can be found at GitHub [49]. To begin with, the initial reads containing coordinate identity (CID) sequences were aligned to the predefined coordinates of the Stereo-seq section, using the results obtained from the first sequencing round as a reference. During this alignment, a maximum of one base mismatch was permitted to accommodate potential sequencing errors. Subsequently, reads with molecular identifiers (MIDs) that had more than 2 bases with a quality score lower than 10 were filtered out to maintain data quality. For each read that passed these criteria, the corresponding CID and MID were integrated into the read header. The remaining reads were then aligned to the reference genome using STAR, and only those with a mapping quality score exceeding 10 were retained for gene annotation, thereby ensuring the precision of gene expression analysis. Unique molecular identifiers (UMIs) with identical CIDs were merged into a single UMI, allowing for a single mismatch to correct any sequencing or PCR errors. Ultimately, the expression profile matrix containing CIDs was generated.

### Image-based single-cell segmentation of Stereo-seq data with StereoCell

AI-assisted cell segmentation was performed on the Stereo-seq data following a previously reported procedure [50]. Briefly, total UMI counts were computed for each DNA nanoball (DNB) coordinate and assembled into a spatial density matrix. The matrix was converted into a grayscale image in which each pixel represented a DNB position and pixel intensity reflected the summed UMI signal. The nucleic-acid staining image from the same tissue section was manually registered to the Stereo-chip coordinate space. Cell segmentation was then carried out using an ESPANet-based deep learning model. Predicted masks were refined using watershed-based post-processing to generate binary cell segmentation results. For each segmented cell, UMIs from all DNBs within the cell mask were aggregated at the gene level, yielding a cell-by-gene count matrix for downstream analyses.

### Stereo-Seq data analysis and visualization

To comprehensively characterize the spatial transcriptomic landscape within the region, the bin size of 100 (100 × 100, 50 × 50 μm) was utilized as a fundamental unit (spot) for subsequent analysis. After generating the Bin100 data, we performed quality control on it, and finally retained Bin units with a mitochondrial gene ratio of less than 20% and the number of detected genes greater than 500. Ultimately, the average data quality of bin100 was 2,470 genes/bin100 and 5,573 UMIs/bin100. The expression data matrix derived from these bin100 units, inclusive of their spatial coordinates, was subsequently employed to construct a Seurat object utilizing the Seurat package in R (v4.2.2).

### Spatial transcriptome hippocampal region clustering

Transcriptome data from the defined partitions (bin100) on each section were utilized to generate normalized SingleCellExperiment objects, which served as input for subsequent BayesSpace clustering [20]. The BayesSpace package (v1.8.0) was used to cluster the data, thereby annotating the brain regions. Brain regions were annotated and delineated using marker genes identified from clustering subgroups through the *FindAllMarkers* function. The 2D outlines of these brain regions were processed and recognized using the imager R package (v0.42.16). This approach enabled the precise description of hippocampal regions, facilitating the annotation of hippocampal regions and the exploration of heterogeneity within the CA1 region.

### Quality control and cell clustering of snRNA-seq dataset

During data preprocessing, 2 filtering criteria were applied to remove low-quality cells: cells with a mitochondrial gene ratio exceeding 10%, and cells with fewer than 500 detected genes. The data were normalized via the *NormalizeData* function. The top 3,000 highly variable genes were selected using the *FindVariableFeatures* function. Then, the expression data was scaled to eliminate technical variation using *ScaleData* function. For different samples, we used the Harmony package (v0.1) to integrate the samples, thereby eliminating the impact of inter-sample differences. For downstream clustering and visualization, dimension reduction based on principal component analysis (PCA) was first performed, and the first 30 principal components (PCs) were extracted for subsequent Louvain clustering to define cell clusters. Finally, the clustering results were visualized in a 2-dimensional space using Uniform Manifold Approximation and Projection (UMAP), and the cell clusters were annotated. Cell type annotation was conducted based on the information labeled in the original data. After completing the integration and clustering of single-cell data using Seurat functions, we further extracted cells from the Sub and CA1 regions for secondary clustering based on the cell annotation information within the data, aiming to exclude potential interference from CA2–4 and DG cells. Finally, by comprehensively analyzing the “clustering results of cells from all neuronal brain regions” and the “independent clustering results of Sub and CA1 cells,” we collectively verified the expression pattern of Sub region-specific genes.

### Cell type annotation of segmented cells by Spatial-ID

Cell type annotation on segmented Stereo-seq data was performed using Spatial-ID [35], implemented with PyTorch (v1.13.1), PyTorch Geometric (v2.1.0), and Python (v3.9.10). The workflow consisted of 2 stages: first, a 4-layer deep neural network was trained on snRNA-seq data using shared genes as anchors to generate initial cell type probabilities for Stereo-seq cells. Second, a graph convolutional network integrated spatial context encoded in an adjacency matrix (normalized Euclidean distances) with gene expression data. This GCN architecture comprised a deep autoencoder for gene expression, a variational graph autoencoder for spatial neighborhoods, and a classifier module. The autoencoders extracted latent representations via self-supervised reconstruction losses, while the classifier mapped these features to cell types through supervised learning aligned with initial probability distributions. Joint optimization of all components over 300 epochs refined cell type assignments. Final annotations were determined by maximum GCN-derived probabilities, effectively reducing batch effects across Stereo-seq sections.

### Quality control of single-cell stereo-seq data

The MID count matrix from Stereo-seq underwent quality control filtering with the Seurat R package across all 24 chips. Cells expressing fewer than 100 genes or containing mitochondrial transcripts above 20% were excluded to ensure data quality. This filtering strategy was uniformly applied to maintain consistency across sections. The average quality of cellbin data was 430 genes/cell and 767 UMIs/cell.

### Correlation analysis of cell types between stereo-seq and snRNA-seq data

Normalization of Stereo-seq and snRNA-seq data was conducted using Seurat’s RNA assay framework. Top variable genes common to both datasets were identified and subjected to Pearson correlation analysis to establish cell type correspondence between the 2 platforms.

### Aligning Bin100 and cellbin for brain region annotation

For brain region annotation of cellbin data, we first propagated the brain region annotation information from bin100 data to bin1 units. Subsequently, based on the positional correspondence between each bin1 and cellbin, we assigned the brain region annotations from bin1 to the corresponding cellbin, thereby achieving brain region annotation of the cellbin data.

### Differential expression analysis

For Sub-specific genes and AD-specific genes in different brain regions, FindMarkers function of Seurat was used to analyze the DEGs between the AD and Con samples (log_2_FC  >  0.25, *P*  <  0.05, min.pct > 0.1). For subsequent visualization and statistical comparison of selected DEGs, expression values were first summarized within each donor for the corresponding anatomical region. Each donor was treated as an independent biological replicate. Group-level values are presented as the mean ± standard error of the mean (SEM), calculated across donors, rather than across individual cells or spatial spots. Statistical comparisons between groups were likewise performed using donor-level summarized expression values.

### Functional enrichment analysis

Disease ontology (DO) analysis was performed using the EnrichDO function in the clusterProfiler R software package [51] with the following parameters: *P*.adjust = 0.05.

Gene ontology (GO) analysis was performed using the EnrichGO function in the clusterProfiler R software package with the following parameters: *P*.adjust = 0.05.

### Identification of clinically significant modules

Gene co-expression network analysis was performed using the hdWGCNA R package (v0.2.17) [31]. A co-expression network was constructed from the spatial transcriptomic dataset, and the resulting adjacency matrix was transformed into a topological overlap matrix (TOM). Genes were hierarchically clustered according to TOM-based dissimilarity and assigned to co-expression modules. Module eigengenes were then calculated and correlated with AD status and Sub identity to identify disease- and region-associated modules. Within modules showing significant associations, hub genes were defined according to their intramodular connectivity.

### Protein–protein interaction network construction and hub gene identification

The protein–protein interaction (PPI) network was visualized by Cytoscape software (v3.10.0) [52]. The cytoHubba plug-in in Cytoscape was employed to identify hub genes in PPI network based on degree method [53]. The components in the central node could be considered as the core proteins and significant hub genes with essential physiological functions.

### RNAscope validation of marker genes

RNAscope™ Multiplex Fluorescent v2 assay for fixed frozen samples was performed using the RNAscope™ Multiplex Fluorescent Kit (Cat. No. 323100) according to the manufacturer’s instructions (sbio Technology, Inc.). Briefly, to achieve close spatial and anatomical correspondence with the spatial transcriptomic data, RNAscope validation experiments using 2 × 2 cm capture areas were performed on cryopreserved, nearby region-matched tissue sections collected from the same hippocampal tissue blocks used for Stereo-seq profiling [18]. These sections were anatomically matched to the Stereo-seq sections but were not necessarily serially adjacent sections. Each section was used for orthogonal validation of candidate CA1–Sub marker genes. In total, 2 sections were used for this validation assay, one from the cognitively control group and one from a donor with AD. Tissues were fixed in fresh 4% PFA (1 × PBS) at 4°C for 24 h, embedded in OCT post-sucrose gradient treatment, frozen with dry ice/liquid nitrogen, stored at −80°C, sectioned into 7–15 μm slices on SuperFrost® Plus slides (Epredia, 4951PLUS-001E), dried at −20°C for 60–120 min, washed with 1 × PBS, baked at 60°C for 30 min, and post-fixed in pre-cooled 10% NBF/4% PFA at 4°C for 15 min before dehydration with gradient ethanol and air-drying. The following fluorescence channels were used for RNAscope probes: *FN1* (C1) and *ETV1* (C2). Sections were counterstained with DAPI (RT, 1–2 min), mounted with ProLong Gold (Thermo, P36930).

### Quantification of RNAscope fluorescence intensity in CA1 and sub regions

RNAscope fluorescence quantification was performed on annotated fluorescence images for *FN1* and *ETV1*. The CA1 and Sub regions were defined according to the white annotated boundaries in each image. For each chip, 10 square subareas were randomly sampled within the CA1 region, and 10 square subareas were randomly sampled within the Sub region. Subareas were retained only when they fell within the corresponding annotated region. Target fluorescence was quantified from the magenta signal channel. Background intensity was estimated from dark background pixels in the same image and subtracted from the raw fluorescence intensity. Negative background-corrected values were set to zero. For each sampled subarea, the mean background-corrected fluorescence intensity was calculated and used as one technical measurement. For each gene and condition, fluorescence intensity was summarized as mean ± SEM. CA1 and Sub fluorescence intensities were compared using a 2-sided Welch’s *t*-test. Statistical significance was indicated as ns, *P* ≥ 0.05; * *P* < 0.05; ** *P* < 0.01; *** *P* < 0.001; and ^****^  *P* < 0.0001. These measurements represent image-level technical quantification of regional fluorescence enrichment.

## Additional files

**Supplementary Table 1**. Summary metrics from the CNBB cohort.

**Supplementary Table 2**. Group-level comparison of demographic, clinical, and tissue-quality characteristics.

**Supplementary Table 3**. Marker genes for the 4 BayesSpace-defined clusters within the *MPPED1*-defined CA1–Sub region.

**Supplementary Table 4**. Specific Expression Genes in Sub and CA1 Regions.

**Supplementary Table 5**. DEGs between AD and Con in Sub and CA1 regions.

**Supplementary Table 6**. Overlapping Genes Between Sub-Specific Markers and AD-Related DEGs in Sub.

**Supplementary Table 7**. Overlapping Genes Between CA1-Specific Markers and AD-Related DEGs in CA1.

**Supplementary Table 8**. Gene lists of hdWGCNA co-expression modules identified from AD-associated differentially expressed genes.

**Supplementary Table 9**. GO enrichment results by different module eigengenes.

## Availability of source code and requirements

Project name: ST-Human-Hippocampus-Boundary

Project homepage: https://github.com/L-Yuyang/2025_ST-Human-Hippocampus-Boundary

License: MIT license

Operating system: Linux, macOS, Windows

Programming language: R and Python

Package management: CRAN, Bioconductor and Conda

Hardware requirements: Recommended at least 8-core CPU and 64 GB RAM for processing high-resolution Stereo-seq dataset

## Supplementary Material

giag094_Supplemental_File

giag094_Authors_Response_To_Reviewer_Comments_original_submission

giag094_GIGA-D-26-00145_original_submission

giag094_GIGA-D-26-00145_revision_1

giag094_Reviewer_1_Report_original_submission Reviewer 1 -- 6/2/2026

giag094_Reviewer_1_Report_revision_1 Reviewer 1 -- 8/12/2026

giag094_Reviewer_2_Report_original_submission Reviewer 2 -- 6/10/2026

## Data Availability

The datasets supporting the findings of this study are available from public repositories. The high-resolution spatial transcriptomic (Stereo-seq) data have been deposited in the CNGB Nucleotide Sequence Archive (CNSA) of the China National GeneBank DataBase (CNGBdb) under accession code CNP0005077. The publicly available mouse brain spatial transcriptomic data used for cross-species validation were obtained from Han et al. (2025) [28] and are available from CNSA under accession code CNP0003837. The RNAscope images evaluated in this study, together with the *in situ* hybridization images showing *Fn1* and *Etv1* expression in the mouse brain obtained from the Allen Mouse Brain Atlas, are also publicly available through the same GitHub repository (ST-Human-Hippocampus-Boundary/tree/main/Validation_Images). The single-nucleus RNA sequencing (snRNA-seq) data utilized in this study were obtained from the Gene Expression Omnibus (GEO) under accession number GSE186538, which includes transcriptomic profiles microdissected from multiple human hippocampal and parahippocampal regions, including the dentate gyrus (DG), CA2–4, CA1, Sub, and entorhinal cortex (EC).

## References

[1] Iglesias JE, Augustinack JC, Nguyen K et al. A computational atlas of the hippocampal formation using ex vivo, ultra-high resolution MRI: application to adaptive segmentation of in vivo MRI. Neuroimage. 2015;115:117–37. 10.1016/j.neuroimage.2015.04.042. PMC4461537 25936807

[2] Tosches MA, Yamawaki TM, Naumann RK et al. Evolution of pallium, hippocampus, and cortical cell types revealed by single-cell transcriptomics in reptiles. Science. 2018;360:881–88. 10.1126/science.aar4237. 29724907

[3] Lisman J, Buzsáki G, Eichenbaum H et al. Viewpoints: how the hippocampus contributes to memory, navigation and cognition. Nat Neurosci. 2017;20:1434–47. 10.1038/nn.4661. PMC5943637 29073641

[4] Donato F, Alberini CM, Amso D et al. The ontogeny of hippocampus-dependent memories. J Neurosci. 2021;41:920926. 10.1523/jneurosci.1651-20.2020. PMC7880290 33328296

[5] Berdugo‐Vega G, Dhingra S, Calegari F. Sharpening the blades of the dentate gyrus: how adult-born neurons differentially modulate diverse aspects of hippocampal learning and memory. EMBO J. 2023;42:e113524. 10.15252/embj.2023113524. PMC11059975 37743770

[6] O’Mara SM, Commins S, Anderson M et al. The subiculum: a review of form, physiology and function. Prog Neurobiol. 2001;64:129–55. 10.1016/s0301-0082(00)00054-x. 11240210

[7] Sun Y, Jin S, Lin X et al. CA1-projecting subiculum neurons facilitate object-place learning. Nat Neurosci. 2019;22:1857–70. 10.1038/s41593-019-0496-y. PMC6819262 31548723

[8] Mizuseki K, Kitanishi TJCOiN. Oscillation-coordinated, noise-resistant information distribution via the subiculum. Curr Opin Neurobiol. 2022;75:102556. 10.1016/j.conb.2022.102556. 35598550

[9] Huxter JR, Senior TJ, Allen K et al. Theta phase–specific codes for two-dimensional position, trajectory and heading in the hippocampus. Nat Neurosci. 2008;11:587–94. 10.1038/nn.2106. 18425124

[10] Kitanishi T, Umaba R, Mizuseki K. Robust information routing by dorsal subiculum neurons. Sci Adv. 2021;7:eabf1913. 10.1126/sciadv.abf1913. PMC7946376 33692111

[11] Romani A, Antonietti A, Bella D et al. Community-based reconstruction and simulation of a full-scale model of the rat hippocampus CA1 region. PLoS Biol. 2024;22:e3002861. 10.1371/journal.pbio.3002861. PMC11537418 39499732

[12] Liu X, Li F, Czosnyka M et al. Multi-omics and high-spatial-resolution omics: deciphering complexity in neurological disorders. GigaScience. 2025;14:giaf137. 10.1093/gigascience/giaf137. PMC12723665 41348969

[13] Cao L, Yang C, Hu L et al. Deciphering spatial domains from spatially resolved transcriptomics with Siamese graph autoencoder. GigaScience. 2024;13:giae003. 10.1093/gigascience/giae003. PMC10939418 38373745

[14] Zhou L, Peng X, Chen M et al. Unveiling patterns in spatial transcriptomics data: a novel approach utilizing graph attention autoencoder and multiscale deep subspace clustering network. GigaScience. 2025;14:giae103. 10.1093/gigascience/giae103. PMC11727722 39804726

[15] Kang J, Li Q, Liu J et al. Exploring the cellular and molecular basis of murine cardiac development through spatiotemporal transcriptome sequencing. GigaScience. 2025;14:giaf012. 10.1093/gigascience/giaf012. PMC11831923 39960664

[16] Rosenblum EW, Williams EM, Champion SN et al. The prosubiculum in the human hippocampus: a rostrocaudal, feature-driven, and systematic approach. J Comp Neurol. 2024;532:e25604. 10.1002/cne.25604. PMC11060218 38477395

[17] Adler DH, Pluta J, Kadivar S et al. Histology-derived volumetric annotation of the human hippocampal subfields in postmortem MRI. Neuroimage. 2014;84:505–23. 10.1016/j.neuroimage.2013.08.067. PMC3864597 24036353

[18] Wang P, Han L, Wang L et al. Molecular pathways and diagnosis in spatially resolved Alzheimer’s hippocampal atlas. Neuron. 2025;113:2123–2140.e9. 10.1016/j.neuron.2025.03.002. 40168986

[19] El Hayek L, DeVries D, Gogate A et al. Disruption of the autism gene and chromatin regulator KDM5A alters hippocampal cell identity. Sci Adv. 2023;9:eadi0074 10.1126/sciadv.adi0074. PMC10664992 37992166

[20] Zhao E, Stone MR, Ren X et al. Spatial transcriptomics at subspot resolution with BayesSpace. Nat Biotechnol. 2021;39:1375–84. 10.1038/s41587-021-00935-2. PMC8763026 34083791

[21] Franjic D, Skarica M, Ma S et al. Transcriptomic taxonomy and neurogenic trajectories of adult human, macaque, and pig hippocampal and entorhinal cells. Neuron. 2022;110:452–469.e14. 10.1016/j.neuron.2021.10.036. PMC8813897 34798047

[22] Fei F, Shi J, Xi J et al. Fibronectin1-Expressing subicular circuits selectively govern the retrieval of novel object recognition. Adv Sci. 2026;13:e16399. 10.1002/advs.202516399. PMC13334995 41984531

[23] Tao Y, Dai P, Liu Y et al. Erbin regulates NRG1 signaling and myelination. Proc Natl Acad Sci. 2009;106:9477–82. 10.1073/pnas.0901844106. PMC2695084 19458253

[24] Podvin S, Wojnicz A, Hook V. Human brain gene expression profiles of the cathepsin V and cathepsin L cysteine proteases, with the PC1/3 and PC2 serine proteases, involved in neuropeptide production. Heliyon. 2018;4:e00673. 10.1016/j.heliyon.2018.e00673. PMC6037879 29998195

[25] Rigter PMF, de Konink C, Dunn MJ et al. Role of CAMK2D in neurodevelopment and associated conditions. Am Hum Genet. 2024;111:364–82. 10.1016/j.ajhg.2023.12.016. PMC10870144 38272033

[26] Thompson JR, Nelson ED, Tippani M et al. An integrated single-nucleus and spatial transcriptomics atlas reveals the molecular landscape of the human hippocampus. Nat Neurosci. 2025;28:1990–2004. 10.1038/s41593-025-02022-0. PMC12411265 40739059

[27] Lein ES, Hawrylycz MJ, Ao N et al. Genome-wide atlas of gene expression in the adult mouse brain. Nature. 2007;445:168–76. 10.1038/nature05453. 17151600

[28] Han L, Liu Z, Jing Z et al. Single-cell spatial transcriptomic atlas of the whole mouse brain. Neuron. 2025;113:2141–2160.e9. 10.1016/j.neuron.2025.02.015. 40132589

[29] Cadonic C, Sabbir MG, Albensi BC. Mechanisms of mitochondrial dysfunction in Alzheimer’s disease. Mol Neurobiol. 2016;53:6078–90. 10.1007/s12035-015-9515-5. 26537901

[30] Adav SS, Park JE, Sze SK. Quantitative profiling brain proteomes revealed mitochondrial dysfunction in Alzheimer’s disease. Mol Brain. 2019;12:8. 10.1186/s13041-019-0430-y. PMC6350377 30691479

[31] Morabito S, Reese F, Rahimzadeh N et al. hdWGCNA identifies co-expression networks in high-dimensional transcriptomics data. Cell Rep Methods. 2023;3:100498. 10.1016/j.crmeth.2023.100498. PMC10326379 37426759

[32] Hong BV, Zheng J, Agus JK et al. High-density lipoprotein changes in Alzheimer’s disease are APOE genotype-specific. Biomedicines. 2022;10:1495. 10.3390/biomedicines10071495. PMC9312991 35884800

[33] Fleeman RM, Kuhn MK, Chan DC et al. Apolipoprotein E ε4 modulates astrocyte neuronal support functions in the presence of amyloid-β. J Neurochem. 2023;165:536–49. 10.1111/jnc.15781. PMC10903110 36762973

[34] Azarnia Tehran D, Kochlamazashvili G, Pampaloni NP et al. Selective endocytosis of Ca(2+)-permeable AMPARs by the Alzheimer’s disease risk factor CALM bidirectionally controls synaptic plasticity. Sci Adv. 2022;8:eabl5032. 10.1126/sciadv.abl5032. PMC9132451 35613266

[35] Shen R, Liu L, Wu Z et al. Spatial-ID: a cell typing method for spatially resolved transcriptomics via transfer learning and spatial embedding. Nat Commun. 2022;13:7640. 10.1038/s41467-022-35288-0. PMC9741613 36496406

[36] Ding SL . Comparative anatomy of the prosubiculum, subiculum, presubiculum, postsubiculum, and parasubiculum in human, monkey, and rodent. J Comp Neurol. 2013;521:4145–62. 10.1002/cne.23416. 23839777

[37] Ishizuka NJJoCN . Laminar organization of the pyramidal cell layer of the subiculum in the rat. J Comp Neurol. 2001;435:89–110. 10.1002/cne.1195. 11370013

[38] Yushkevich PA, Amaral RSC, Augustinack JC et al. Quantitative comparison of 21 protocols for labeling hippocampal subfields and parahippocampal subregions in in vivo MRI: towards a harmonized segmentation protocol. Neuroimage. 2015;111:526–41. 10.1016/j.neuroimage.2015.01.004. PMC4387011 25596463

[39] Cembrowski MS, Wang L, Lemire AL et al. The subiculum is a patchwork of discrete subregions. eLife. 2018;7:e37701. 10.7554/eLife.37701. PMC6226292 30375971

[40] Ding S-L, Yao Z, Hirokawa KE et al. Distinct transcriptomic cell types and neural circuits of the subiculum and prosubiculum along the dorsal-ventral axis. Cell Rep. 2020;31:107648. 10.1016/j.celrep.2020.107648. PMC7394561 32433957

[41] González-Arnay E, Pérez-Santos I, Jiménez-Sánchez L et al. Immunohistochemical field parcellation of the human hippocampus along its antero-posterior axis. Brain Struct Funct. 2024;229:359–85. 10.1007/s00429-023-02725-9. PMC10917878 38180568

[42] Maynard KR, Collado-Torres L, Weber LM et al. Transcriptome-scale spatial gene expression in the human dorsolateral prefrontal cortex. Nat Neurosci. 2021;24:425–36. 10.1038/s41593-020-00787-0. PMC8095368 33558695

[43] Lv T, Zhang Y, Li M et al. EAGS: efficient and adaptive gaussian smoothing applied to high-resolved spatial transcriptomics. GigaScience. 2024;13:giad097. 10.1093/gigascience/giad097. PMC10939424 38373746

[44] Ding SL, Van Hoesen GWJJoCN. Organization and detailed parcellation of human hippocampal head and body regions based on a combined analysis of cyto-and chemoarchitecture. J Comp Neurol. 2015;523:2233–53. 10.1002/cne.23786. 25872498

[45] Pitceathly RDS, Rahman S, Wedatilake Y et al. NDUFA4 mutations underlie dysfunction of a cytochrome c oxidase subunit linked to human neurological disease. Cell Rep. 2013;3:1795–805. 10.1016/j.celrep.2013.05.005. PMC3701321 23746447

[46] Williams CG, Lee HJ, Asatsuma T et al. An introduction to spatial transcriptomics for biomedical research. Genome Med. 2022;14:68. 10.1186/s13073-022-01075-1. PMC9238181 35761361

[47] Hyman BT, Phelps CH, Beach TG et al. National Institute on Aging–Alzheimer’s Association guidelines for the neuropathologic assessment of Alzheimer’s disease. Alzheimers Dement. 2012;8:1–13. 10.1016/j.jalz.2011.10.007. PMC3266529 22265587

[48] Rao A, Barkley D, França GS et al. Exploring tissue architecture using spatial transcriptomics. Nature. 2021;596:211–20. 10.1038/s41586-021-03634-9. PMC8475179 34381231

[49] Gong C, Li S, Wang L et al. SAW: an efficient and accurate data analysis workflow for stereo-seq spatial transcriptomics. GigaByte. 2024;2024:1–12. 10.46471/gigabyte.111. PMC10905255 38434930

[50] Zhang B, Li M, Kang Q et al. Generating single-cell gene expression profiles for high-resolution spatial transcriptomics based on cell boundary images. GigaByte. 2024;2024:1–13. 10.46471/gigabyte.110. PMC10905256 38434932

[51] Yu G, Wang LG, Han Y et al. clusterProfiler: an R package for comparing biological themes among gene clusters. Omics. 2012;16:284–87. 10.1089/omi.2011.0118. PMC3339379 22455463

[52] Shannon P, Markiel A, Ozier O et al. Cytoscape: a software environment for integrated models of biomolecular interaction networks. Genome Res. 2003;13:2498–504. 10.1101/gr.1239303. PMC403769 14597658

[53] Chin C-H, Chen S-H, Wu H-H et al. cytoHubba: identifying hub objects and sub-networks from complex interactome. BMC Syst Biol. 2014;8:S11. 10.1186/1752-0509-8-s4-s11. PMC4290687 25521941

