# Supplementary material for: A molecularly anchored spatial transcriptomic framework for precise CA1–subiculum parcellation and region-resolved analysis in Alzheimer’s disease
Source: Gigascience. 2026 Sep 17;15:giag094. doi: 10.1093/gigascience/giag094 (PMC13674357; doi:10.1093/gigascience/giag094)
Supplement: Supplementary file 1 [file giag094_authors_response_to_reviewer_comments_original_submission.pdf]

Author's Response To Reviewer Comments

Please note that this text field does not support embedded figures. We have therefore uploaded two separate files in the Files section: a "Cover Letter" summarizing the major revisions and a complete "Response to Reviewers" containing our point-by-point responses and all rebuttal figures. A text-only version of our responses is provided below.

Reviewer reports:
Reviewer #1: Manuscript #GIGA-D-26-00145 "A Molecularly Anchored Spatial Transcriptomic Framework for Precise CA1-Subiculum Parcellation and Region-esolved Analysis in Alzheimer's Disease" by Y. Liu et al., leverages publicly available data obtained by spatial transcriptomics (Stereo-seq) and snRNA-seq from Wang et al., 2025 and Franjic et al., 2022 to establish boundaries of key hippocampal regions, namely the CA1 sector and the subiculum. Discerning the anatomic boundaries between the CA1 sector and subiculum are notoriously difficult, especially within the postmortem human brain. The authors utilize several advanced bioinformatics techniques and AI supported programs to identify specific genetic markers (e.g., FN1 with support from ETV1, PCSK1, and CAMK2G) to delineate CA1-subiculum boundaries, and present a viable panel of potential markers for demarcation. This mapping heuristic may be useful in the context of normal subjects as well as inform neurodegenerative disorders including Alzheimer's disease (AD). There is a lot of information to unpack in this study! The authors are urged to reconsider the organization of the manuscript for readership. Below are major and minor considerations to improve an interesting and potentially impactful manuscript.
Response: We sincerely thank reviewer for the thorough and constructive evaluation of our manuscript, and for recognizing the potential significance of this work in delineating hippocampal boundaries and advancing our understanding of Alzheimer's disease. We have carefully addressed all comments raised, and our point-by-point responses are provided below.

Major
1. Manuscript stewardship. The entire manuscript would benefit from a hard copyedit from a native English speaking collaborator who can help shape the narrative. Reliance on online/AI grammar is not recommended as the issues of organization and higher order integration require collaborative assistance.
Response: We thank the reviewer for this valuable suggestion. In response, we have enlisted the help of a professional academic editing service to conduct a thorough hard copyedit of the entire manuscript, strictly avoiding reliance on AI tools as advised. Beyond grammatical corrections, we undertook a comprehensive revision focused on improving the scientific narrative and higher order integration. Specifically, we reorganized the presentation of the results, eliminated redundant statements, and strengthened the logical transitions between sections. We believe these revisions have substantially improved the clarity, coherence, and accessibility of the manuscript, and we would be pleased to make any further adjustments considered necessary.

2. Tissue sample access. Stereo-seq data was obtained from Wang et al., 2025. RNAscope validation appears to have been conducted on a subset of tissues. It is unclear if the authors had direct access to the tissues or if they are from another cohort. This needs to be specified for the assays. Moreover, the exact relationship with Wang et al., 2025 needs to be stated in the Methods and whether (or not) this study is a reanalysis of previously acquired data or a follow-up of shared tissues.
Response: We thank the reviewer for this valuable comment. We agree that provenance of the spatial transcriptomic data and the relationship between the present study and Wang et al. (2025) should be clearly specified.
(1) All Stereo-seq spatial transcriptomic data analyzed in this study were derived from the previously published human hippocampal Stereo-seq dataset reported by Wang et al. (2025). No new Stereo-seq sequencing experiments or additional spatial transcriptomic data generation were performed in the present study. Instead, this work represents a secondary computational reanalysis of the previously acquired spatial transcriptomic resource using a molecularly anchored framework for CA1-Sub parcellation and region-resolved analysis of AD. The original dataset consisted of a larger collection of human hippocampal Stereo-seq sections. For the present study, we selected 24 tissue sections from 12 specimens (6 AD and 6 Control) based on predefined criteria, including tissue integrity, anatomical preservation, and the presence

of an identifiable CA1-Sub region required for downstream parcellation analyses. The inclusion status and corresponding selection criteria for each chip have now been clarified in Supplementary Table 1. We have added this information to the Methods section (Pages 30-31, lines 591-613) and the Result section (Page 5, lines 94-104).

(2) The RNAscope validation experiments were performed using cryopreserved adjacent tissue sections from the same human hippocampal tissue cohort described in Wang et al. (2025). During the original tissue preparation process Stereo-seq profiling, adjacent sections from the same tissue blocks and anatomical regions were preserved. These matched sections were subsequently used for RNAscope validation. This approach ensures precise spatial and anatomical matching with the sequencing data. Therefore, the RNAscope experiments in this study represent an orthogonal tissue-level validation using anatomically matched human sections from the same cohort, rather than a validation performed on a separate external patient cohort. We have clarified this relationship in the revised Methods section and specified that the present study combines secondary computational analysis of previously published Stereo-seq data with additional targeted molecular validation using adjacent cryopreserved tissue sections (Pages 35, lines 747-754).

3. Tissue handling. The authors need to state specifically how tissues were handled and prepared including tissue fixation procedures, size and number of sections used for snRNA-seq and RNAscope.

Response: We thank the reviewer for pointing out the need for clarification regarding our tissue handling and preparation protocols. Followed the reviewers' recommendation, we have updated the detailed descriptions of RNAscope validation experiments in the Methods section, including the tissue fixation procedures, size and number of sections used for RNAscope (Pages 35, lines 747-754).

We have also clarified that the snRNA-seq data analyzed in this study were not newly generated by us. Instead, the snRNA-seq dataset was retrieved from the public GEO database under accession number GSE186538 and was used for secondary analysis. Therefore, no new tissue handling, tissue sectioning, or fixation procedures for snRNA-seq were performed in the present study. The relevant descriptions have been added to the Methods section (Page 30, lines 570-576; Pages 32-33, lines 662-679).

4. Tissue selection criteria. An explanation of how tissues were included (and excluded) is warranted. The Reviewer suggests a Supplementary image panel of what is acceptable or not acceptable tissue quality for postmortem hippocampal and subiculum sections.

Response: We thank the reviewer for this constructive suggestion regarding tissue selection and quality-control criteria. We agree that a clear description of tissue inclusion and exclusion is important for interpreting the CA1–Sub-specific analyses.

During the anatomical annotation phase, our primary inclusion criterion for CA1–subiculum-specific analyses was whether the tissue section contained an identifiable and analyzable subiculum (Sub) region. Tissue sections that did not encompass an analyzable Sub region were therefore excluded from downstream CA1–Sub analyses. Only sections containing both the relevant hippocampal anatomy and an identifiable Sub region were retained for subsequent CA1–Sub-specific analyses (Rebuttal Fig 1a). In addition to this anatomy-based filtering, we further evaluated chip-level heterogeneity using principal component analysis (PCA). During this quality-control step, two AD chips, AD3.1 and AD3.2, showed marked separation from the remaining chips in the PCA space, indicating substantial heterogeneity relative to the rest of the cohort (Rebuttal Fig 1b). To avoid introducing chip-level outlier effects into the downstream spatial and transcriptomic analyses, AD3.1 and AD3.2 were excluded from the final CA1–Sub analyses.

In response to the reviewer's suggestion, we have expanded Supplementary Table 1, "Summary metrics from the CNBB cohort" to include tissue-selection and quality-control information. Specifically, we added a column indicating whether the Sub region was present and analyzable for each chip, as well as the final inclusion/exclusion status and the corresponding exclusion reason. This provides a transparent overview of how tissues were included or excluded across the cohort. We also added Supplementary Table 2, which summarizes the group-level comparisons of demographic, clinical, and tissue-quality variables between the AD and control cohorts, together with the corresponding statistical tests and p values. The text describing these tissue-selection and quality-control criteria has also been updated in the Methods section (Pages 30-31, lines 595-613).

Rebuttal Fig. 1: Tissue inclusion and chip-level quality control for CA1–Sub analyses.

(a) Anatomy-based tissue selection. Sections were retained only if they contained an identifiable and

analyzable Sub region suitable for CA1–Sub comparison. Sections lacking an analyzable Sub region were excluded from downstream CA1–Sub-specific analyses.
(b) PCA-based chip-level quality control. Principal component analysis showed that AD3.1 and AD3.2 were markedly separated from the remaining chips, indicating substantial global transcriptomic heterogeneity. These two chips were excluded from the final CA1–Sub analyses to reduce chip-level outlier effects.

5. AI assistance verification. Was manual validation of AI-generated segmentation performed? Greater details regarding QA/QC is strongly recommended to indicate validity of the AI techniques used.
Response: Thank you for raising this important point. We have added additional quality-control and validation analyses to support the reliability of the cell segmentation, cell-type annotation, and downstream spatial transcriptomic quantification. These analyses are now included in Extended Data Fig. 6.
First, we evaluated the cell segmentation performance using three representative image fields. Manual inspection-based benchmarking showed a high detection rate, with an average Recall of 0.92 ± 0.02 and F1 score of 0.82 ± 0.02 across the three fields (Extended Data Fig. 6a, lines 409-410). This indicates that the segmentation procedure successfully detected -the majority of cells. The main remaining limitation was occasional over-segmentation in densely packed or clustered regions, which we now explicitly acknowledge.
Second, we added a canonical marker-gene heatmap to validate cell-type annotation. Major neuronal, glial, vascular, immune and ependymal populations showed expected marker enrichment, including excitatory neuronal markers, inhibitory neuronal markers, oligodendrocyte/OPC markers, astrocyte, microglial, endothelial, pericyte and ependymal markers. This supports the biological consistency of the annotated cell classes (Extended Data Fig. 6b, lines 410-412).
Third, we added chip-level quality-control plots comparing UMI counts and detected gene numbers across control and AD samples. These QC distributions showed comparable overall sequencing/capture quality across chips and conditions, supporting that the observed biological differences are unlikely to be driven by gross technical imbalance (Extended Data Fig. 6c-d, lines 412-414).
Together, these additional analyses strengthen the technical validation of the segmentation, annotation and data quality. We have added these results the relevant Results text (Page 20, lines 353-359).

6. RNAscope. The RNAscope approach requires greater detail (e.g., case demographics, technical strategy) along with the method of comparison to spatially derived data from Wang et al., 2025.
Response: We thank the reviewer for the opportunity to clarify the technical strategy and demographic pairing of our validation cohort.
Technical Strategy & Sample Pairing: The tissue sections used for RNAscope validation were cryopreserved sections obtained from corresponding anatomical regions within the same hippocampal tissue blocks used for Stereo-seq profiling in Wang et al. (2025). This tight spatial coupling ensures that our validation assays directly reflect the transcriptomic microenvironments captured in the discovery dataset. We have updated the relevant Methods text (Pages 35, lines 747-754).
Case Demographics & Chip Metadata: In alignment with the reviewer's comment, we have integrated the detailed case demographics, clinical parameters from Wang et al., 2025 into Supplementary Table 1.

7. Discussion. Despite the large amount of data and supportive validation, the Discussion is skeletal. The authors are urged to rewrite the Discussion to reflect the impact of the current boundary delineation findings as well as reframe postmortem human tissue based studies attempting to define the CA1-subiculum continuum in the context of existing literature.
Response: We thank the reviewer for this constructive suggestion. We agree that the original Discussion did not sufficiently emphasize the broader significance of our CA1–Sub parcellation framework or place the findings in the context of postmortem human hippocampal studies.
We have therefore substantially rewritten and expanded the Discussion. In the revised manuscript, we now discuss why the CA1–Sub interface is difficult to define in postmortem human tissue, how gradual cytoarchitectural and molecular transitions complicate traditional anatomical annotation, and how our molecularly anchored framework provides a reproducible strategy for region-resolved analysis. We also clarify that the CA1–Sub interface should be interpreted as a gradual molecular transition zone and that our annotations represent operational, molecularly informed parcellations rather than evidence for a sharp anatomical border. In addition, we now discuss the impact of this framework for postmortem tissue studies, including its potential to reduce regional misclassification, improve reproducibility, and increase the analyzable use of scarce human hippocampal specimens. We also expanded the AD-related discussion to

present the disease findings as a proof-of-concept application and to acknowledge limitations related to sample size, Braak-stage heterogeneity, APOE genotype, and postmortem tissue constraints.
Overall, the revised Discussion now better reflects the impact, context, and limitations of the study. The relevant content has been updated in the Discussion section of the revised manuscript (Pages 25-29, lines 419–558).

Minor
1. Please restructure the manuscript for conventional readership by presenting approaches in the Methods, findings in the Results, and interpretations in the Discussion. The original submission has these sections dispersed throughout the text.
Response: We thank the reviewer for this helpful suggestion. We agree that the original manuscript would benefit from a more conventional and reader-friendly organization.
In the revised manuscript, we have reorganized both the content and the overall structure of the manuscript. Specifically, we adjusted the section order so that the manuscript now presents the Results first, followed by the Discussion and then the Methods, in accordance with the journal format. Within this revised structure, methodological details, including data sources, preprocessing steps, segmentation, annotation, statistical analyses, and validation procedures, have been consolidated into the Methods section. The Results section has been revised to focus on the main findings and supporting analyses, whereas broader interpretation, comparison with existing literature, implications, and limitations have been moved to the Discussion section.

2 . Relocate Source code from the Methods to the Data Availability section.
Response: We thank the reviewer for this suggestion. We have relocated the source code information from the Methods section to the Data Availability section in the revised manuscript. The revised Data Availability section now includes the project name, project homepage, license, operating system, programming language, package management information, and hardware requirements (Pages 37, lines 791-804). This change improves compliance with the journal format and separates code availability information from the methodological descriptions.

3. Are images of the sections that the authors evaluated publicly available? Please upload them to a repository such as www.synapse.org.
Response: We thank the reviewer for this helpful suggestion. The RNAscope images evaluated in this study, together with the in situ hybridization images showing Fn1 and Etv1 expression in the mouse brain obtained from the Allen Mouse Brain Atlas, have now been made publicly available through our GitHub repository (https://github.com/L-Yuyang/2025_ST-Human-Hippocampus-Boundary/tree/main/Validation_Images). The original data sources and relevant image information from the Allen Mouse Brain Atlas are clearly indicated in the repository. We have also added the repository link to the revised manuscript to facilitate public access and independent evaluation (Page 37, lines 796-800).

4. There is an enormous amount of spatial and sequence data. Please curate genelists for ease of readership and consider making them available as Supplementary material.
Response: We thank the reviewer for this helpful suggestion. We agree that curated gene lists are important for improving readability, transparency, and reuse of the spatial and sequencing analyses. In the revised manuscript, we have curated the major gene lists generated and used in this study and made them available as Supplementary Tables.
Specifically, we have added Supplementary Table 3, entitled "Marker genes for the four BayesSpace-defined clusters within the MPPED1-defined CA1–subiculum region" which provides the marker genes supporting the BayesSpace-based molecular parcellation of the CA1–Sub region. We have also added Supplementary Table 8, entitled "Gene lists of hdWGCNA co-expression modules identified from AD-associated differentially expressed genes" which provides the gene composition of the hdWGCNA modules used for network-level interpretation of AD-associated changes.
Together with the existing supplementary materials, the revised manuscript now includes nine Supplementary Tables. These tables are referenced in the relevant Results sections and provide curated donor information, cluster markers, region-associated genes, and network/module gene lists to help readers navigate and reuse the large spatial and sequence datasets analyzed in this study.

5. Little information is given to the AD cohort. Since AD hallmark pathology is present in the CA1 and

subiculum, how is this expected to impact the ability to delineate these boundaries?
Response: We thank the reviewer for raising this important point.
In the revised manuscript, we have clarified the information of AD cohort (Page 5, lines 100-104; Page 31, lines 611-615) and summarized the available demographic and pathological metadata in Supplemental Table 1.
In the original manuscript, CA1- and Sub-enriched genes were identified from control samples to establish the baseline molecular features of the CA-Sub interface. This molecular framework was substantially applied to AD samples to evaluate whether AD-associated pathology affects the ability to delineate this regional boundary. We found that the expression patterns of representative markers were largely preserved in AD samples (Extended Data Fig. 2g, line 179; Fig. 3f-g, lines 245-247). To further evaluate whether AD-associated pathology affects the robustness of this boundary, we performed additional differential expression analyses separately in control and AD samples. The results showed that the regional expression direction of the core CA1/Sub markers was largely preserved in both groups. In particular, the Sub-enriched markers identified in our original analysis, including FN1, ETV1, PCSK1, and CAMK2G, retained more highly expressed in Sub than in CA1 in AD samples (Rebuttal Fig. 3). These results suggest that the major molecular features underlying CA1–Sub regional identity remain detectable despite AD-associated transcriptional alternations. Together. these analyses support the robustness of our molecularly informed CA1–Sub parcellation framework in AD tissues, while we acknowledge that AD pathology may influence the magnitude of some regional transcriptional signals.

Rebuttal Fig. 3: Consistency of CA1–Sub regional marker patterns in control and AD tissues.
Scatter plot comparing the log2 fold change of Sub versus CA1 expression in control samples and AD samples. The strong positive correlation indicates that the regional enrichment direction of CA1- and Sub-associated genes is largely preserved across diagnostic groups, with representative Sub-enriched markers, including FN1, ETV1, PCSK1, and CAMK2G, remaining consistently enriched in the Sub region.

Reviewer #2: Summary
This study addresses the persistent challenge of reliably defining the CA1-subiculum boundary in hippocampal research. To resolve inconsistencies, the authors employ complementary methods Stereo-seq spatial transcriptomics and single-nucleus RNA-seq on 12 human donors (6 with Alzheimer's disease and 6 controls). They identify FN1 as a molecular marker of the subiculum, with support from ETV1, PCSK1, and CAMK2G, and validate these findings using RNAscope and an independent dataset. Applying this molecular boundary to Alzheimer's tissue reveals region-specific changes, most notably increased mitochondrial and energy-metabolism gene expression in the subiculum. The study is interesting and potentially valuable, though several issues remain to be addressed.
Response: We thank the reviewer for the thoughtful summary and positive assessment of our study. We appreciate the recognition of the potential value of defining the CA1–Sub boundary using complementary spatial transcriptomic, single-nucleus, RNAscope, and external validation approaches. We have carefully considered the reviewer’s comments and have addressed each point in detail below.

Major
1. Clarify the origin of the data. The paper reads as if a new 12-donor dataset was generated, but the Stereo-seq, snRNA-seq, and 10X data all come from earlier studies (Wang 2025; Franjic 2022; Thompson 2025); only the RNAscope is new. State this plainly and explain how the study relates to Wang 2025.
Response: We thank the reviewer for highlighting the need to clarify the origin of the data. We have revised the manuscript to state explicitly that the Stereo-seq, snRNA-seq, and 10X datasets were all obtained from previously published studies, namely Wang et al., 2025, Franjic et al., 2022, and Thompson et al., 2025, respectively. No new Stereo-seq, snRNA-seq, or 10X sequencing datasets were generated in this study. The only newly generated experimental data were from RNAscope validation (Page 30, lines 562-589; Page 35, lines 747-750).
We have also clarified the relationship between the RNAscope validation samples and the Stereo-seq dataset reported by Wang et al. (2025). The tissue sections used for RNAscope validation were cryopreserved sections obtained from corresponding anatomical regions within the same hippocampal tissue blocks used for Stereo-seq profiling in Wang et al. (2025). They were therefore anatomically matched to the Stereo-seq sections, although they were not necessarily serially adjacent sections. This sampling strategy provided close spatial and anatomical correspondence between the RNAscope validation assays and the transcriptomic regions analyzed in the discovery dataset (Page 35, lines 747-750).

2. Strengthen the statistical analysis. Testing a large number of bins or cells does not compensate for the small cohort of 6 Alzheimer's disease donors and 6 control donors, as the donor is the appropriate unit of analysis. Therefore, the main results should be recomputed at the donor level using suitable models, such as pseudobulk or mixed-effects approaches.
Response: We thank the reviewer for this important and constructive comment. As suggested, we have therefore recomputed the key analyses at the donor level using donor-level mean expression and pseudobulk-based approaches. First, for the regional enrichment analysis of MPPED1 in Fig. 1b, we recalculated MPPED1 expression at the donor level. Specifically, MPPED1 expression was averaged within CA1 and within the other hippocampal regions for each chip, and donor-level paired comparisons were then performed separately in the control and Alzheimer's disease groups. The revised donor-level analysis confirmed that MPPED1 remained enriched in CA1 compared with other hippocampal regions in both groups (Fig. 1b, lines 122-127). Second, we further validated the expression patterns of FN1, ETV1, PCSK1, and CAMK2G by calculating their average expression in CA1 and Sub for each donor/chip-level sample. The donor-level regional mean expression analysis showed that these genes were consistently higher in the Sub than in CA1, supporting their use as Sub-enriched markers (Fig. 3c and e, lines 240-245; Extended Data Fig. 3d-e, lines 257-259). The corresponding procedures for donor-level aggregation and statistical analysis have also been described in the revised Methods section (Page 34, lines 716-721).

3. Provide direct evidence that FN1 marks neurons. Although FN1 is primarily expressed in the extracellular matrix and vasculature bordering the subiculum, the observed elevated FN1 signal may reflect non-neuronal contamination. Therefore, the authors should provide direct evidence that FN1 marks subicular neurons.
Response: We thank the reviewer for this important comment. In the published snRNA-seq dataset analyzed in our study (Franjic et al., 2022; PMID: 34798047, was selectively expressed in a subicular excitatory neuronal population annotated as the FN1-positive subtype. In addition, Fei et al. (2026; PMID: 41984531) provided another anatomical evidence supporting the expression of FN1 in Sub projection neurons. TB-555, a retrograde neuronal tracer that labels neurons projecting to the injection site, whose injection into the entorhinal cortex showed that FN1 expression was predominantly associated with retrogradely labeled neurons in the Sub.
We also performed additional validation using the CellBin-level spatial transcriptomic data in our study. At the cell-bin resolution, we examined FN1 expression across annotated cell types and brain regions. This analysis showed that FN1 was highly expressed in neuronal cells within the Sub region. In parallel, ETV1, another marker identified in our analysis, also showed elevated expression in Sub neuronal populations (Rebuttal Fig. 4).
Importantly, we have revised the wording throughout the manuscript to avoid overgeneralizing FN1 as a pan-neuronal marker. Instead, we now describe FN1 as a marker enriched in a subset of Sub neurons and use it together with ETV1 and other markers as part of a molecular panel for CA1–Sub boundary delineation. We also acknowledge that FN1 has known extracellular matrix and vascular associations (PMID: 38598053), and therefore its interpretation is strengthened by cell-type-resolved CellBin analysis and independent anatomical evidence (Page 2, lines 36-37; Page 7, lines 150-151; Page 25, lines 424-426).
,

Rebuttal Fig. 4: CellBin-level expression of FN1 and ETV1 across CA1 and Sub cell types.
Dot plot showing scaled expression and percentage of expressing cells for FN1 and ETV1 across annotated CellBin cell types in the Sub and CA1. FN1 and ETV1 showed higher expression in EX_Sub than in the corresponding CA1 neuronal and non-neuronal populations, supporting their association with subicular neuronal populations.

4.No evidence is provided that the Alzheimer's and control groups were matched for post-mortem interval, RNA quality, or cause of death. Several leading Alzheimer's disease genes (FOS, JUNB, NR4A, NPAS4) are stress- and death-response genes, so bin-level results may confound cell loss with real expression changes. These factors weaken the metabolic findings.
Response: We thank the reviewer for raising this important concern. As suggested, we have expanded Supplementary Table 1 to include all available donor-level metadata on post-mortem interval (PMI), RNA integrity number (RIN), Braak stage, and Thal stage, and have summarized the corresponding group-level

comparisons in updated Supplementary Table 2. Using donors as independent statistical units, we did not detect statistically significant differences between AD and control groups in PMI (Welch's t-test, $p = 0.36$) or RIN ($p = 0.15$). However, given the cohort size, we do not interpret these non-significant results as formal evidence of equivalence. Complete cause-of-death information was not available for all donors; therefore, residual confounding related to agonal state or terminal events cannot be excluded.
To assess whether reduced EX_CA1 abundance contributed to the observed expression differences, we first quantified EX_CA1 density in the AD and control groups. EX_CA1 density was lower in the AD group than in the control group (Rebuttal Fig. 5a; Wilcoxon test, $p < 0.05$), indicating that differences in neuronal abundance could potentially influence the bin-level comparison. We therefore performed a density-matched subsampling sensitivity analysis in which EX_CA1-assigned cells from the control group were down-sampled to approximate the EX_CA1 density distribution observed in the AD group. After downsampling, we did not detect a statistically significant group difference in EX_CA1 density (Rebuttal Fig. 5b; Wilcoxon test, $p > 0.05$), and the neuronal module scores were also similar between the density-matched AD and control groups (Rebuttal Fig. 5c; Wilcoxon test, $p > 0.05$). We then repeated the differential-expression analysis using these density-matched EX_CA1 cells. Among the four activity- and stress-responsive genes highlighted by the reviewer, JUNB retained the same direction of change and remained significant according to our predefined statistical criteria, whereas FOS, NR4A1, and NPAS4 no longer met those criteria (Rebuttal Fig. 5d). Because subsampling reduces the number of observations and therefore statistical power, the loss of significance for these three genes should not be interpreted as evidence that their original differences were caused solely by neuronal loss. Rather, this sensitivity analysis indicates that the JUNB signal was more robust to density-matched subsampling, whereas the evidence for FOS, NR4A1, and NPAS4 was less stable.
We agree that neither density-matched downsampling nor cell-type-restricted analysis can fully distinguish AD-associated transcriptional responses from responses related to neuronal stress. degeneration, agonal state, or other unmeasured donor-level factors. We have therefore revised the Result and Discussion to avoid presenting these genes as definitive AD-specific alterations. We have also moderated the interpretation of the associated metabolic findings and now describe them as exploratory transcriptional associations rather than direct evidence of altered metabolic function (Page 2, line 43; Page 16, lines 293-296; Page 27, lines 516-519).

Rebuttal Fig. 5: Density-matched analysis of EX_CA1 cellbins reduces the potential confounding effect of neuronal loss on transcriptional differences between AD and Con.
(a) Comparison of EX_CA1 cell-bin density between the Alzheimer's disease (AD) and control groups before downsampling. EX_CA1 density was significantly higher in the control group than in the AD group. (b) Comparison of EX_CA1 cell-bin density after downsampling the control group to match the density distribution observed in AD. (c) Comparison of neuronal module scores between the density-matched AD and control EX_CA1 cell bins. (d) Differential-expression analysis of density-matched EX_CA1 cell bins between the AD and control groups. Among the stress- and death-response genes highlighted by the reviewer, JUNB remained significantly differentially expressed.

5. Resolve the conflicting boundary description. While graded markers and proximal/distal neuron subtypes suggest a gradual transition, the text describes a sharp boundary. This contradiction should be addressed, and strong claims about boundary sharpness should be tempered accordingly.
Response: We thank the reviewer for pointing out this important inconsistency. We have therefore revised the manuscript to avoid overstatement of boundary sharpness. Specifically, we have replaced descriptions such as "sharp boundary" or "clear-cut boundary" with more accurate terms, including "gradual transition", "molecular transition zone" and "operationally defined CA1-Sub interface". We now clarify that the purpose of our framework is not to impose an artificial sharp border, but to use spatially graded molecular markers to define a reproducible and biologically informed transition zone between CA1 and the Sub. We have also revised the Discussion to explicitly state that proximal/distal neuronal subtypes and graded marker expression are consistent with a continuum model of the CA1-Sub interface. Accordingly, the boundary annotations in this study should be interpreted as molecularly informed operational parcellations for region-resolved analysis, rather than as evidence for an abrupt anatomical border. The relevant content has been updated in revised manuscript (Page 25, lines 446-447; Page 26, lines 473-474; Page 10, lines 197-198; Page 11, lines 222-223; Page 26, lines 468-469; Page 28, lines 552-554).

Minor

6. Validation is limited: RNAscope was performed for only two genes, assessed visually with minimal sampling, and 10X validation is incomplete. The legend of Extended Data Fig. 1 reads "Con," while the figure is titled "AD"; this requires correction.
Response: We thank the reviewer for this critical comment. We agree that the original validation was limited in both marker breadth and quantitative sampling. To strengthen the validation, we added quantitative analysis of the RNAscope images for two representative markers FN1 and ETV1. Both the analyzed control section and the analyzed AD section showed higher FN1 and ETV1 signals in the Sub than in CA1 (Extended Data Fig. 3f–g, lines 259-261). We have added the complete image-sampling and quantification procedures to the Methods (Page 35, lines 762-775).
In addition, we have expanded the analysis of the independent 10X spatial transcriptomics transcriptomic dataset from Thompson et al. 2025. We included all available sections containing both CA1 and Sub according to the anatomical annotations, comprising 4 sections from 3 donors. To avoid treating individual spatial spots as independent biological replicates, gene expression was summarized at the section level within each anatomical region, and paired Sub–CA1 expression differences were calculated for FN1 and ETV1. Both genes showed a consistent Sub-enriched expression pattern in 4/4 of the evaluable sections (Extended Data Fig. 3h-j, lines 261-264). Since only two sections contained the complete CA1–Sub continuum, we now describe this analysis as supportive external cross-platform validation rather than comprehensive validation of the entire boundary (Page 30, lines 577-578).
Given the limited number of human hippocampal sections available for RNAscope validation, we additionally examined publicly available in situ hybridization data from the Allen Mouse Brain Atlas. Across multiple mouse brain sections and independent image series, both Fn1 and Etv1 showed prominent expression in the Sub region (Extended Data Fig. 4a-b, lines 268-271). In addition, analysis of two publicly available mouse hippocampal spatial transcriptomic sections generated by Han et al. (2025) showed that Fn1 and Etv1 consistently exhibited higher expression in the anatomically annotated Sub than in neighboring hippocampal regions (Extended Data Fig. 4c–e, lines 271-274). These publicly available anatomical data provide independent, cross-species support for the association of FN1 and ETV1 with the Sub, while we acknowledge that they do not substitute for direct validation in additional human samples.
Finally, we revised the Results and Discussion to avoid overstatement. Specifically, we now describe the RNAscope, Allen Mouse Brain Atlas, and 10X Genomics analyses as complementary, orthogonal, and supportive validation of representative Sub-enriched markers, rather than as exhaustive validation of all candidate genes or definitive validation of the complete CA1-Sub boundary. RNAscope provides direct validation in human tissue, the Allen Mouse Brain Atlas offers independent cross-species anatomical support, and the external 10X dataset provides cross-platform confirmation in human spatial transcriptomic data (Page 27, lines 500-504).
We rechecked the sample annotation in Extended Data Fig. 1. The incorrect “Con” label in the legend has been corrected to “AD” to match the corresponding figure panel and sample identity (Page 7, line 138).

7. Donor information is incomplete: APOE genotype, sex, age, and post-mortem interval are not reported, and the Alzheimer's disease cases span a broad pathological range (Braak III-V) within a small cohort.
Response: We thank the reviewer for pointing out this important issue. In the revised manuscript, we have added detailed donor-level information, including age, sex, post-mortem interval (PMI), APOE genotype, diagnosis, and Braak stage, to Supplementary Table 1. We have also added Supplementary Table 2, which summarizes the group-level comparisons of demographic, clinical, and tissue-quality variables between the AD and control cohorts, together with the corresponding statistical tests and p values. The control cases showed absent to low Braak pathology, ranging from not detected to Braak stage II, whereas the AD cases ranged from Braak stage III to V, with most AD cases classified as Braak stage IV. The relevant description has also been added to the Methods section (Pages 31, lines 611-615). We agree that the AD cohort spans a pathological range within a relatively small sample size. We have therefore revised the manuscript to interpret AD-associated findings more cautiously and now explicitly acknowledge that Braak-stage heterogeneity may contribute to inter-donor variability (Page 28, lines 535-540). Future studies with larger cohorts and more balanced pathological staging will be needed to further assess how AD stage, APOE genotype, and other donor-level variables influence CA1–Sub boundary delineation and region-resolved disease signatures.

8. Inaccurate method description. The manuscript describes hdWGCNA as characterizing gene correlation patterns in microarray samples. This describes the original WGCNA; hdWGCNA was specifically designed for high-dimensional single-cell and spatial transcriptomic data, which is the data type used here. Please

clarify.
Response: We thank the reviewer for pointing out this inaccurate description. We agree that the previous wording incorrectly referred to microarray samples, which describes the original WGCNA framework rather than hdWGCNA. This was a wording error in the manuscript. In the present study, we used hdWGCNA, which is designed for high-dimensional single-cell and spatial transcriptomic datasets. We have revised the Methods section to accurately describe hdWGCNA as a framework for identifying gene co-expression modules in high-dimensional single-cell/spatial transcriptomic data. The incorrect reference to microarray samples has been removed from the revised manuscript (Page 34, lines 730-736).

Editor comments:

The manuscript would benefit from a hard copyedit by a native English speaker or a professional language editing service to improve fluency and clarity.
Thank you for this valuable suggestion. In response, have enlisted the help of a professional academic editing service to conduct a thorough hard copyedit of the entire manuscript, strictly avoiding reliance on AI tools as advised. Beyond grammatical corrections, we undertook a comprehensive revision focused on improving the scientific narrative and higher order integration. Specifically, we reorganized the presentation of the results, eliminated redundant statements, and strengthened the logical transitions between sections. We believe these revisions have substantially improved the clarity, coherence, and accessibility of the manuscript, and we would be pleased to make any further adjustments considered necessary.

Editorial Manager®

Cookie Preference Center

We use cookies which are necessary to make our site work. We may also use additional cookies to analyze, improve and personalize our content and your digital experience. For more information, see our Cookie Policy.

You may choose not to allow some types of cookies. However, blocking some types may impact your experience of our site and the services we are able to offer. See the different category headings below to find out more or change your settings.

Allow all

Manage Consent Preferences

Strictly Necessary Cookies

Always active

These cookies are necessary for the website to function and cannot be switched off in our systems. They are usually only set in response to actions made by you which amount to a request for services, such as setting your privacy preferences, logging in or filling in forms. You can set your browser to block or alert you about these cookies, but some parts of the site will not then work.

Functional Cookies

These cookies enable the website to provide enhanced functionality and personalisation. They may be set by us or by third party providers whose services we have added to our pages. If you do not allow these cookies then some or all of these services may not function properly.

Performance Cookies

These cookies allow us to count visits and traffic sources so we can measure and improve the performance of our site. They help us to know which pages are the most and least popular and see how visitors move around the site.

Targeting Cookies

These cookies may be set through our site by our advertising partners. They may be used by those companies to build a profile of your interests and show you relevant adverts on other sites. If you do not allow these cookies, you will experience less targeted advertising.

Cookie List

Clear

Apply Cancel

Consent Leg.Interest

Confirm my choices

Power
