# Supplementary material for: A molecularly anchored spatial transcriptomic framework for precise CA1–subiculum parcellation and region-resolved analysis in Alzheimer’s disease
Source: Gigascience. 2026 Sep 17;15:giag094. doi: 10.1093/gigascience/giag094 (PMC13674357; doi:10.1093/gigascience/giag094)
Supplement: Supplementary file 2 [file giag094_giga-d-26-00145_original_submission.pdf]

GigaScience

A Molecularly Anchored Spatial Transcriptomic Framework for Precise CA1–Subiculum Parcellation and Region-Resolved Analysis in Alzheimer's Disease

--Manuscript Draft--

**Manuscript Number:**	GIGA-D-26-00145
**Full Title:**	A Molecularly Anchored Spatial Transcriptomic Framework for Precise CA1–Subiculum Parcellation and Region-Resolved Analysis in Alzheimer's Disease
**Article Type:**	Research
**Funding Information:**	Key Program of the National Natural Science Foundation of China (32530027) — Prof. Jing Zhang<br>Key Program of the National Natural Science Foundation of China (82501681) — Doc. Pan Wang<br>National Science and Technology Innovation 2030 Major Program (2021ZD0204400) — Doc. Lei Han
**Abstract:**	Background: The precise molecular delineation of the interface between the Subiculum (Sub) and cornu ammonis 1 (CA1) is a long-standing challenge that limits the reproducibility of hippocampal research. Conventional cytoarchitectural boundaries are often ambiguous, leading to inconsistent regional annotation and the underuse of scarce human brain specimens. To resolve this, we present a molecularly anchored spatial transcriptomic framework designed to transform subjective anatomical challenges into a verifiable molecular reference. By integrating high-definition spatial transcriptomics (Stereo-seq) with single-nucleus RNA sequencing (snRNA-seq) across 12 human donors, we developed a data-driven parcellation pipeline that defines discrete transcriptomic domains with high spatial fidelity, transcending traditional anatomical constraints.<br>Findings: A core output of this framework is a stringent molecular marker panel established through systematic cross-modality benchmarking. We identified FN1 as a definitive, Sub-restricted boundary marker and provided a complementary marker, ETV1, to ensure reproducible subfield annotation across diverse datasets. To demonstrate the utility and robustness of this reference, we applied the framework to Alzheimer’s Disease (AD) tissues. This application confirms that our pipeline enables precise, region-resolved investigations even under pathological conditions, unmasking divergent transcriptional signatures, such as region-specific bioenergetic remodeling, that are otherwise obscured by conventional anatomical mapping.<br>Conclusions: Collectively, this study provides a standardized molecular reference and a transferable workflow for human CA1–Sub parcellation. By replacing subjective anatomical interpretation with a data-driven molecular coordinate system, this framework facilitates anatomically grounded, high-resolution investigations of the human hippocampus. This resource offers immediate utility for enhancing the reproducibility of both basic neurobiological studies and clinical investigations into neurodegenerative pathologies.
**Corresponding Author:**	Lifang Wang<br>BGI Research, Hangzhou<br>Hangzhou, CHINA
**Corresponding Author Secondary Information:**	
**Corresponding Author's Institution:**	BGI Research, Hangzhou
**Corresponding Author's Secondary Institution:**	
**First Author:**	Lifang Wang
**First Author Secondary Information:**	
**Order of Authors:**	Lifang Wang

Powered by Editorial Manager® and ProduXion Manager® from Aries Systems Corporation

	Yuyang Liu
	Youzhe He
	Yanrong Wei
	Pan Wang
	Chunyu Huang
	Quyuan Tao
	Langjian Zhu
	Xun Xu
	Longqi Liu
	Lei Han
	Jing Zhang
**Order of Authors Secondary Information:**	
**Additional Information:**	
**Question**	**Response**
Are you submitting this manuscript to a special series or article collection?	No
**Experimental design and statistics**<br><br>Full details of the experimental design and statistical methods used should be given in the Methods section, as detailed in our Minimum Standards Reporting Checklist. Information essential to interpreting the data presented should be made available in the figure legends.<br><br>Have you included all the information requested in your manuscript?	Yes
**Resources**<br><br>A description of all resources used, including antibodies, cell lines, animals and software tools, with enough information to allow them to be uniquely identified, should be included in the Methods section. Authors are strongly encouraged to cite Research Resource Identifiers (RRIDs) for antibodies, model organisms and tools, where possible.<br><br>Have you included the information requested as detailed in our Minimum	Yes

Powered by Editorial Manager® and ProduXion Manager® from Aries Systems Corporation

Standards Reporting Checklist?	
**Availability of data and materials**<br><br>All datasets and code on which the conclusions of the paper rely must be either included in your submission or deposited in publicly available repositories (where available and ethically appropriate), referencing such data using a unique identifier in the references and in the "Availability of Data and Materials" section of your manuscript.<br><br>Have you have met the above requirement as detailed in our Minimum Standards Reporting Checklist?	Yes
GigaScience has policies and guidelines in place for the use of generative AI-writing tools such as ChatGPT. If you have used such writing tools to assist with writing the manuscript this must be declared and cited in the text. Authors should not list AI-writing tools and other AI-assisted technologies as an author or co-author and should acknowledge that they are fully responsible for text generated or refined by AI-writing tools.<p><br><br>A summary of use (particularly in the introduction or among methods) needs to be included at the end of the paper, and the outputs should also be included as a supplementary file hosted in GigaDB or other open repositories. Please read our guidelines for more information. <p><br><br>By submitting to GigaScience, you are aware of the journal's AI-writing tools policy, and if you have declared use of such tools below, you have acknowledged this where appropriate in your manuscript and have made a summary of use and outputs available. <p><br><b>AI-assisted writing tools have been used in the preparation of this manuscript?	No

Manuscript
Click here to access/download;Manuscript;02_Sub_Manu260512_V3.5.docx

A Molecularly Anchored Spatial Transcriptomic Framework for Precise CA1–Subiculum Parcellation and Region-Resolved Analysis in Alzheimer's Disease

Yuyang Liu[1,2†], Youzhe He[1,2†], Yanrong Wei[1,2], Pan Wang[3], Chunyu Huang[2], Quyuan Tao[1,2], Langjian Zhu[2], Xun Xu[4,5], Longqi Liu[4,5,6], Lei Han[4,5,6*], Jing Zhang[3*], Lifang Wang[2,7,8*]

Author affiliations:

[1] College of Life Sciences, University of Chinese Academy of Sciences, Beijing 100049, China.

[2] BGI Research, Hangzhou 310030, China.

[3] Department of Pathology, The First Affiliated Hospital, Zhejiang University School of Medicine, Hangzhou, Zhejiang 310002, China.

[4] State Key Laboratory of Genome and Multi-omics Technologies, BGI Research, Hangzhou 310030, China.

[5] State Key Laboratory of Genome and Multi-omics Technologies, BGI Research, Shenzhen 518083, China.

[6] Key Laboratory of Brain Cell Mapping of Zhejiang Province, Hangzhou 310030, China

[7] BGI Research, Shenzhen 518083, China.

[8] Lead contact

[†]These authors contributed equally to this work.

*Correspondence: wanglifang@genomics.cn (L.W.), jzhang1989@zju.edu.cn (J.Z.) hanlei2@genomics.cn (L.H.).

Abstract

Background: The precise molecular delineation of the interface between the Subiculum (Sub) and cornu ammonis 1 (CA1) is a long-standing challenge that limits the reproducibility of hippocampal research. Conventional cytoarchitectural boundaries are often ambiguous, leading to inconsistent regional annotation and the underuse of scarce human brain specimens. To resolve this, we present a molecularly anchored spatial transcriptomic framework designed to transform subjective anatomical challenges into a verifiable molecular reference. By integrating high-definition spatial transcriptomics (Stereo-seq) with single-nucleus RNA sequencing (snRNA-seq) across 12 human donors, we developed a data-driven parcellation pipeline that defines discrete transcriptomic domains with high spatial fidelity, transcending traditional anatomical constraints.

Findings: A core output of this framework is a stringent molecular marker panel established through systematic cross-modality benchmarking. We identified *FN1* as a definitive, Sub-restricted boundary marker and provided a complementary marker, *ETV1*, to ensure reproducible subfield annotation across diverse datasets. To demonstrate the utility and robustness of this reference, we applied the framework to Alzheimer's Disease (AD) tissues. This application confirms that our pipeline enables precise, region-resolved investigations even under pathological conditions, unmasking divergent transcriptional signatures, such as region-specific bioenergetic remodeling, that are otherwise obscured by conventional anatomical mapping.

Conclusions: Collectively, this study provides a standardized molecular reference and a transferable workflow for human CA1–Sub parcellation. By replacing subjective anatomical interpretation with a data-driven molecular coordinate system, this framework facilitates anatomically grounded, high-resolution investigations of the human hippocampus. This resource offers immediate utility for enhancing the reproducibility of both basic neurobiological studies and clinical investigations into neurodegenerative pathologies.

Introduction

The hippocampus is a highly conserved structure composed of distinct anatomical regions, including the dentate gyrus (DG), cornu ammonis (CA1–CA4), and the subiculum (Sub)[1]. These regions are highly conserved during evolution[2], and form critical neural circuits essential for cognitive functions such as memory consolidation, spatial navigation, and emotional regulation[3]. As the primary output gateway of the hippocampal formation, the Sub is positioned between the CA1 region and the entorhinal cortex[4,5]. It serves as a vital relay station, receiving convergent inputs from CA1 and various cortical areas while transmitting processed information to downstream cortical and thalamic structures[6-11]. This anatomical positioning renders the Sub an indispensable hub for information flow within and beyond the hippocampal system, playing a pivotal role in hippocampal-dependent learning and memory.

Despite these functional differences, the precise anatomical and functional boundary between Sub and CA1 remains poorly defined. Traditional histological markers often fail to capture the molecular transitions at this interface, leaving the precise anatomical-functional boundary poorly defined and hindering our understanding of region-specific contributions to hippocampal circuitry.

Precise spatial delineation is paramount for deciphering how the hippocampal formation segregates complex cognitive processing. While traditional transcriptomic methods provide deep cellular insights, they often lack the spatial context and architectural resolution required to resolve these fine-scale anatomical boundaries in the human brain. This lack of a standardized molecular reference prevents researchers from accurately attributing pathological changes, such as those seen in Alzheimer's Disease (AD), to specific hippocampal subfields, potentially masking region-specific disease mechanisms.

In this study, we present a high-definition molecularly anchored framework for human CA1–Sub parcellation. By integrating high-definition spatial transcriptomics (Stereo-seq) with single-nucleus RNA sequencing (snRNA-seq), we established a definitive molecular reference at near-cellular resolution. We report a validated marker panel, headlined by *FN1* and *ETV1*, which provides a reproducible toolkit for subfield annotation. The AD research under this framework demonstrates its application for anatomically grounded interrogation of neurodegenerative pathologies.

Methods

Dataset acquisition and preprocessing

The high-resolution spatial transcriptomic datasets were obtained from the study by Wang et al. (2025)[22] (deposited in CNSA under CNP0005077). For targeted analysis, the CA1 area (encompassing both CA1 and Subiculum) was specifically extracted from the full hippocampal sections based on the expression profile of *MPPED1*. This molecularly defined region was used for all subsequent downstream investigations.

The complementary snRNA-seq datasets were sourced from the research by Franjic et al. (2022)[24] (available via GEO accession GSE186538). These transcriptomic profiles were derived from multiple hippocampal subfields (including DG, CA2–4, CA1, Sub, and EC) microdissected from clinically unremarkable adult human donors.

Spatial Transcriptomic Sample Selection

To ensure high data fidelity, the specimens utilized in this study were sourced from an initial pool of 16 hippocampal samples with RNA integrity number (RIN) > 6.0. From this initial pool, 12 specimens (6 AD and 6 Control) were prioritized for subsequent spatial transcriptomic analysis. The selection was based on a rigorous histological evaluation of tissue architecture, specifically prioritizing samples with a well-preserved and continuous CA1 region, which was essential for resolving the CA1–Subiculum interface. Specimens exhibiting significant tissue fragmentation, freezing artifacts, or incomplete anatomical coverage of the CA1 territory were excluded. This targeted selection ensured that the 12 analyzed samples provided the structural integrity and high-resolution spatial coverage required for robust molecular parcellation.

Stereo-seq raw data processing

The fastq files generated from Stereo-seq were analyzed in accordance with the established pipeline, which can be found at GitHub (https://github.com/STOmics/SAW)[12]. To begin with, the initial reads containing coordinate identity (CID) sequences were aligned to the predefined coordinates of the Stereo-seq section, using the results obtained from the first sequencing round as a reference. During this alignment, a maximum of one base mismatch was permitted to accommodate potential sequencing errors. Subsequently, reads with molecular identifiers (MIDs) that had more than two bases with a quality score lower than 10 were filtered out to maintain data quality. For each read that passed these criteria, the corresponding CID and MID were integrated into the read header. The remaining reads were then aligned to the reference genome using STAR, and only those with a mapping quality score exceeding 10 were retained for gene annotation, thereby ensuring the precision of gene expression analysis. Unique molecular identifiers (UMIs) with identical CIDs were merged into a single UMI, allowing for a single mismatch to correct any sequencing or PCR

errors. Ultimately, the expression profile matrix containing CIDs was generated.

Image-based single-cell segmentation of Stereo-seq data with StereoCell

AI-assisted cell segmentation was performed on the Stereo-seq data following a previously reported procedure[13]. Briefly, total UMI counts were computed for each DNA nanoball (DNB) coordinate and assembled into a spatial density matrix. The matrix was converted into a grayscale image in which each pixel represented a DNB position and pixel intensity reflected the summed UMI signal. The nucleic-acid staining image from the same tissue section was manually registered to the Stereo-chip coordinate space.

Cell segmentation was then carried out using an ESPANet-based deep learning model. Predicted masks were refined using watershed-based post-processing to generate binary cell segmentation results. For each segmented cell, UMIs from all DNBs within the cell mask were aggregated at the gene level, yielding a cell-by-gene count matrix for downstream analyses.

Stereo-Seq data analysis and visualization

To comprehensively characterize the spatial transcriptomic landscape within the region, the bin size of 100 (100×100, 50×50 μm) was utilized as a fundamental unit (spot) for subsequent analysis. After generating the Bin100 data, we performed quality control on it, and finally retained Bin units with a mitochondrial gene ratio of less than 20% and the number of detected genes greater than 500. Ultimately, the average data quality of bin100 was 2,470 genes/bin100 and 5,573 UMIs/bin100. The expression data matrix derived from these bin100 units, inclusive of their spatial coordinates, was subsequently employed to construct a Seurat object utilizing the Seurat package in R (v4.2.2).

Spatial transcriptome hippocampal region clustering

Transcriptome data from the defined partitions (bin100) on each section were utilized to generate normalized SingleCellExperiment objects, which served as input for subsequent BayesSpace clustering[14]. The BayesSpace package (v1.8.0) was used to cluster the data, thereby annotating the brain regions. Brain regions were annotated and delineated using marker genes identified from clustering subgroups through the *FindAllMarkers* function. The 2D outlines of these brain regions were processed and recognized using the imager R package (http://dahtah.github.io/imager/) (v0.42.16). This approach enabled the precise description of hippocampal regions, facilitating the annotation of hippocampal regions and the exploration of heterogeneity within the CA1 region.

Quality control and cell clustering of snRNA-seq dataset

During data preprocessing, two filtering criteria were applied to remove low-quality cells: cells with a mitochondrial gene ratio exceeding 10%, and cells with fewer than 500 detected genes. The data

was normalized via the *NormalizeData* function. The top 3000 highly variable genes were selected using the *FindVariableFeatures* function. Then, the expression data was scaled to eliminate technical variation using *ScaleData* function. For different samples, we used the Harmony package (v0.1) to integrate the samples, thereby eliminating the impact of inter-sample differences. For downstream clustering and visualization, dimension reduction based on Principal Component Analysis (PCA) was first performed, and the first 30 Principal Components (PCs) were extracted for subsequent Louvain clustering to define cell clusters. Finally, the clustering results were visualized in a two-dimensional space using Uniform Manifold Approximation and Projection (UMAP), and the cell clusters were annotated. Cell type annotation was conducted based on the information labeled in the original data.

After completing the integration and clustering of single-cell data using Seurat functions, we further extracted cells from the Sub and CA1 regions for secondary clustering based on the cell annotation information within the data, aiming to exclude potential interference from CA2-4 and DG cells. Finally, by comprehensively analyzing the "clustering results of cells from all neuronal brain regions" and the "independent clustering results of Sub and CA1 cells," we collectively verified the expression pattern of Sub region-specific genes.

Cell type annotation of segmented cells by Spatial-ID

Cell type annotation on segmented Stereo-seq data was performed using Spatial-ID[15], implemented with PyTorch (v1.13.1), PyTorch Geometric (v2.1.0), and Python (v3.9.10). The workflow consisted of two stages: First, a four-layer deep neural network was trained on snRNA-seq data using shared genes as anchors to generate initial cell type probabilities for Stereo-seq cells. Second, a graph convolutional network integrated spatial context encoded in an adjacency matrix (normalized Euclidean distances) with gene expression data. This GCN architecture comprised a deep autoencoder for gene expression, a variational graph autoencoder for spatial neighborhoods, and a classifier module. The autoencoders extracted latent representations via self-supervised reconstruction losses, while the classifier mapped these features to cell types through supervised learning aligned with initial probability distributions. Joint optimization of all components over 300 epochs refined cell type assignments. Final annotations were determined by maximum GCN-derived probabilities, effectively reducing batch effects across Stereo-seq sections.

Quality control of single-cell Stereo-seq data

The MID count matrix from Stereo-seq underwent quality control filtering with the Seurat R package across all 24 chips. Cells expressing fewer than 100 genes or containing mitochondrial transcripts above 20% were excluded to ensure data quality. This filtering strategy was uniformly applied to maintain consistency across sections. The average quality of cellbin data was 430

genes/cell and 767 UMIs/cell.

Correlation analysis of cell types between Stereo-seq and snRNA-seq data

Normalization of Stereo-seq and snRNA-seq data was conducted using Seurat's RNA assay framework. Top variable genes common to both datasets were identified and subjected to Pearson correlation analysis to establish cell type correspondence between the two platforms.

Aligning Bin100 & Cellbin for Brain Region Annotation

For brain region annotation of cellbin data, we first propagated the brain region annotation information from bin100 data to bin1 units. Subsequently, based on the positional correspondence between each bin1 and cellbin, we assigned the brain region annotations from bin1 to the corresponding cellbin, thereby achieving brain region annotation of the cellbin data.

Differential expression analysis

For Sub-specific genes and AD-specific genes in different brain regions, FindMarkers function of Seurat was used to analyze the differentially expressed genes (DEGs) between the AD and Consamples ($\log_2$FC > 0.25, $p < 0.05$, min.pct > 0.1).

Functional enrichment analysis

Disease Ontology (DO) analysis was performed using the EnrichDO function in the clusterProfiler R software package[16] with the following parameters: *p*.adjust = 0.05.
Gene ontology (GO) analysis was performed using the EnrichGO function in the clusterProfiler R software package with the following parameters: *p*.adjust = 0.05.

Identification of Clinically Significant Modules

hdWGCNA is a systems biology approach to characterize correlation patterns between genes in microarray samples[17]. This analysis method is designed to find co-expressed gene modules, explore the associations between gene networks and phenotypes of interest, and core genes in the network, and this approach is used to identify candidate biomarkers or therapeutic targets. hdWGCNA R package (v0.2.17) was used to identify AD traits, Sub region related gene modules and hub genes. The adjacency matrix was converted to a topological overlap matrix (TOM). Genes were clustered into distinct modules based on the TOM-based dissimilarity measure.

Protein-Protein Interaction (PPI) Network Construction and Hub Gene Identification

The PPI network was visualized by Cytoscape software (v3.10.0)[18]. The cytoHubba plug-in in Cytoscape was employed to identify hub genes in PPI network based on degree method[19]. The

components in the central node could be considered as the core proteins and significant hub genes with essential physiological functions.

RNAscope validation of marker genes

RNAscope^{TM} Multiplex Fluorescent v2 assay for fixed frozen samples was conducted using the RNAscope^{TM} Multiplex Fluorescent Kit (Cat. No. 323100) per the manufacturer's instructions (sbio Technology, Inc.). Tissues were fixed in fresh 4% PFA (1× PBS) at 4°C for 24h, embedded in OCT post-sucrose gradient treatment, frozen with dry ice/liquid nitrogen, stored at -80°C, sectioned into 7-15 μm slices on SuperFrost® Plus slides (Epredia, 4951PLUS-001E), dried at -20°C for 60-120min, washed with 1× PBS, baked at 60°C for 30min, and post-fixed in pre-cooled 10% NBF/4% PFA at 4°C for 15min before dehydration with gradient ethanol and air-drying.

The following fluorescence channels were used for RNAscope probes: *FN1* (C1) and *ETV1* (C2). Sections were counterstained with DAPI (RT, 1-2min), mounted with ProLong Gold (Thermo, P36930).

Availability of source code and requirements

Project name: ST-Human-Hippocampus-Boundary

Project homepage: https://github.com/L-Yuyang/2025_ST-Human-Hippocampus-Boundary

License: MIT license

Operating system: Linux, macOS, Windows

Programming language: R and Python

Package management: CRAN, Bioconductor and Conda

Hardware requirements: Recommended at least 8-core CPU and 64 GB RAM for processing high-resolution Stereo-seq dataset

Results

A Data-driven spatial framework resolves the ambiguous CA1–Subiculum boundary

For decades, the precise border between the Subiculum (Sub) and CA1 regions of the hippocampus has remained poorly defined by classical anatomy[20,21]. We addressed this long-standing challenge by developing a high-resolution molecularly anchored parcellation framework. To build this reference, we profiled 12 donated human hippocampal samples (6 Alzheimer's disease (AD) and 6 controls (non-demented)) with Spatial Enhanced Resolution Omics sequencing (Stereo-seq)[22], generating a high-density spatial transcriptomic atlas from 24 tissue sections (**Fig. 1a**, **Supplemental Table 1**). All samples were post-mortem donated hippocampal tissues: 6 were obtained from patients with moderate-to-advanced Alzheimer's disease (AD) (Braak III-V, with abundant Aβ deposition), and the other 6 were age- and sex-matched control samples from individuals without dementia and no pathological changes in the hippocampus. To ensure biological and technical reproducibility, two independent tissue sections were sequenced per donor, yielding a robust spatial atlas across 24 hippocampal chips.

To define the putative CA1 territory, we first leveraged Metallophosphoesterase Domain Containing 1 (*MPPED1*) as a known CA1 marker, observing its stable expression across all samples (**Fig. 1b** and **c**, **Extended Data Fig. 1a**)[23]. Based on MPPED1 expression and histology (H&E staining)[22], we extracted the broad CA1 region from each section (**Fig. 1a**). While this initial demarcation aligned with traditional landmarks, unsupervised clustering (BayesSpace)[14] within the *MPPED1*-defined CA1 unveiled two transcriptionally distinct domains (**Fig. 1d**, **Extended Data Fig. 1b**). In other words, what appeared as one anatomical region harbored hidden gene expression heterogeneity, hinting at a latent boundary. This data-driven parcellation indicates that tissue annotated as a single anatomical field by conventional landmarks contains reproducible molecular heterogeneity, suggesting the presence of a latent boundary at the Sub–CA1 interface.

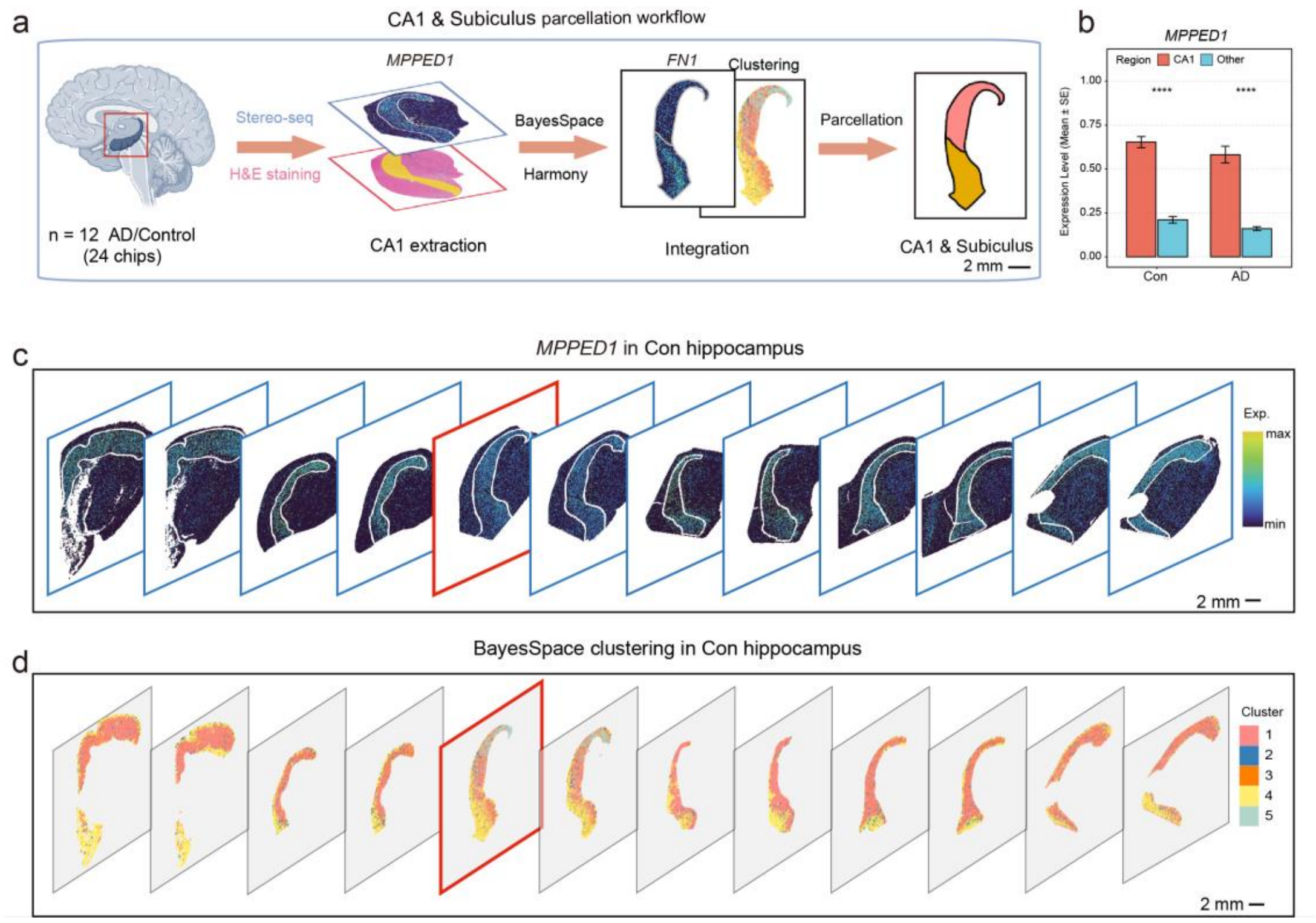

Fig. 1| Region parcellation of human hippocampus by gene expression pattern.

(**a**) The schematic diagram of the study design. A total of 12 samples (24 tissue sections) from AD and Con individuals are performed sequencing and H&E staining experiments. Stereo-seq tissue sections were cut coronally through the hippocampal body. (**b**) Quantitative statistics of the expression level of *MPPED1* gene in CA1 and other brain regions. Difference between CA1 and other regions was examined by *t*-test with Holm's correction, **** $p < 0.0001$. The "Other brain regions" refer to the non-CA1 hippocampal subfields captured on the same Stereo-seq chips, specifically DG, CA2–4, FAS (fimbria, alveus, and stratum oriens (SO)) and SLRM (stratum lucidum (SL), stratum radiatum (SR), stratum moleculare (SM)). (**c**) The expression of *MPPED1* gene in hippocampal CA1 brain region of Con individuals. All Con individuals are age- and sex-matched controls without dementia or hippocampal pathology. The chip marked with a red circle contained two brain regions of relatively moderate size, and this chip would serve as the primary subject for display in the main figures. (**d**) The gene expression of internal heterogeneity in the Con CA1 regions based on BayesSpace algorithm. Scale bar: 2 mm.

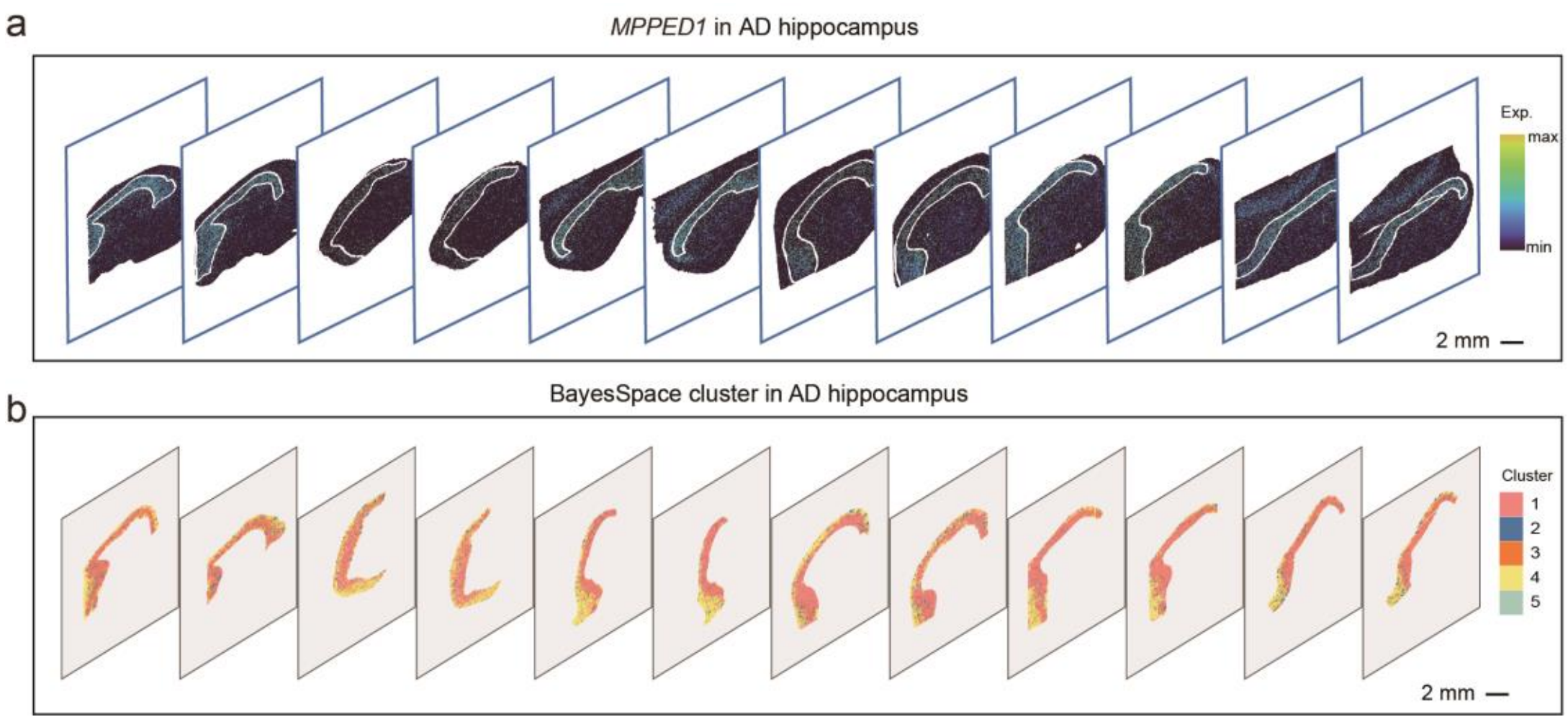

Extended Data Fig. 1| Region parcellation of human hippocampus of AD samples by gene expression pattern.

(**a**) The expression of *MPPED1* gene in hippocampal CA1 brain region in AD patients. (**b**) The gene expression of internal heterogeneity in the Con CA1 regions based on BayesSpace algorithm. Scale bar: 2 mm.

Molecularly anchoring the Sub–CA1 boundary

Given the ambiguous definition of the Sub/CA1 boundary in conventional maps[20,21], we hypothesized that one of the two transcriptomic domains corresponded to the Sub. Notably, BayesSpace Cluster 4 localized to the peripheral edge of the putative CA1 territory, consistent with the expected position of Sub.

To validate this assignment, we integrated our spatial data with single-nucleus RNA-seq profiles from six human hippocampi[24]. Joint clustering of CA1 and Sub cells identified three distinct Sub neuron subtypes (distal Sub, proximal Sub marked by *COL5A2*, and proximal Sub marked by *SEMA3E*) (**Fig. 2a** and **b**, **Extended data Fig. 2a** and **b**). We next screened established hippocampal cell-type markers to find those enriched in Sub. Strikingly, only *FN1* was uniquely and highly expressed in Sub cells (**Fig. 2c** and **d**, **Extended data Fig. 2c-f**), whereas other candidates either marked different regions (e.g., *ROBO1* labeled CA2–4; **Extended Data Fig. 2c**) or lacked specificity (*COL5A2*, *NTNG1* and *SEMA3E* showed some Sub enrichment but also signal in CA1 or CA2–4; **Extended Data Fig. 2c–e**). Crucially, in our spatial atlas *FN1* expression was almost entirely confined to Cluster 4 and absent from the surrounding CA1 (**Fig. 2e**). This one-to-one correspondence unequivocally identified Cluster 4 as the Sub, allowing us to draw a clear molecular boundary between CA1 and Sub regions for the first time (**Fig. 2f, g**; **Extended Data Fig. 2g, h**). In summary, by using *FN1* as a molecular boundary marker, we resolved the previously blurred interface of the Sub within the hippocampal formation.

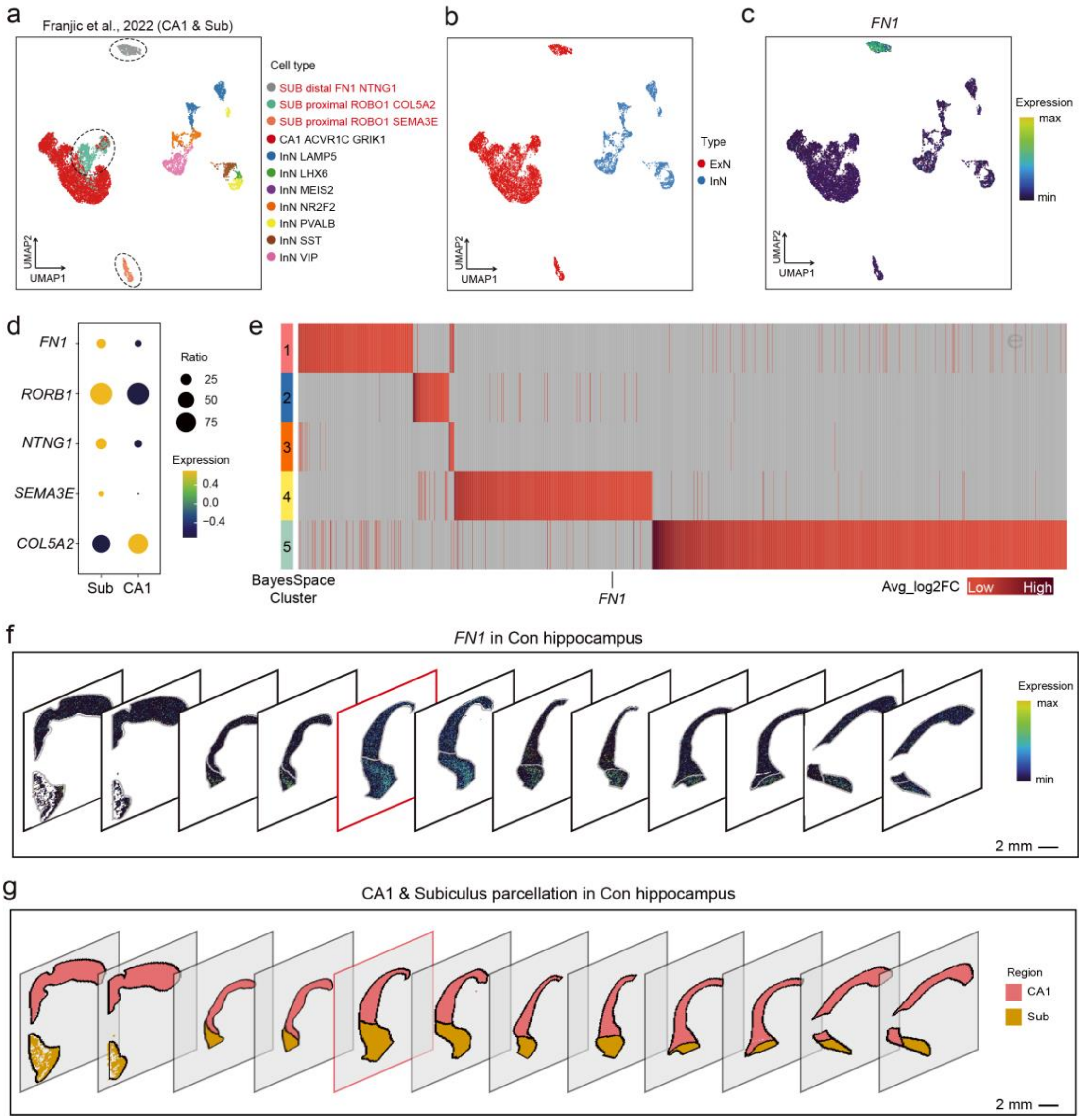

Fig. 2| Identification and screening of marker genes in Sub region using public data.
(**a-b**) UMAP visualization of snRNA dataset from the human hippocampus colored by cell types. (**a**) and neuron types (**b**). The UMAP was obtained by extracting cells from the CA1 and Sub brain regions and then re-clustering them. (**c**) Expression pattern of *FN1* in the UMAP. (**d**) Dot plot showing the expression of reported region-specific genes cross brain regions. (**e**) The heatmap showing the differential expression genes of each cluster in BayesSpace (**Fig. 1d**, **Extended Data Fig. 1b**). (**f**) Spatial expression of *FN1* in the Con hippocampal regions. (**g**) Parcellation of the Sub and CA1 regions in the Con hippocampal regions. Scale bar: 2 mm.

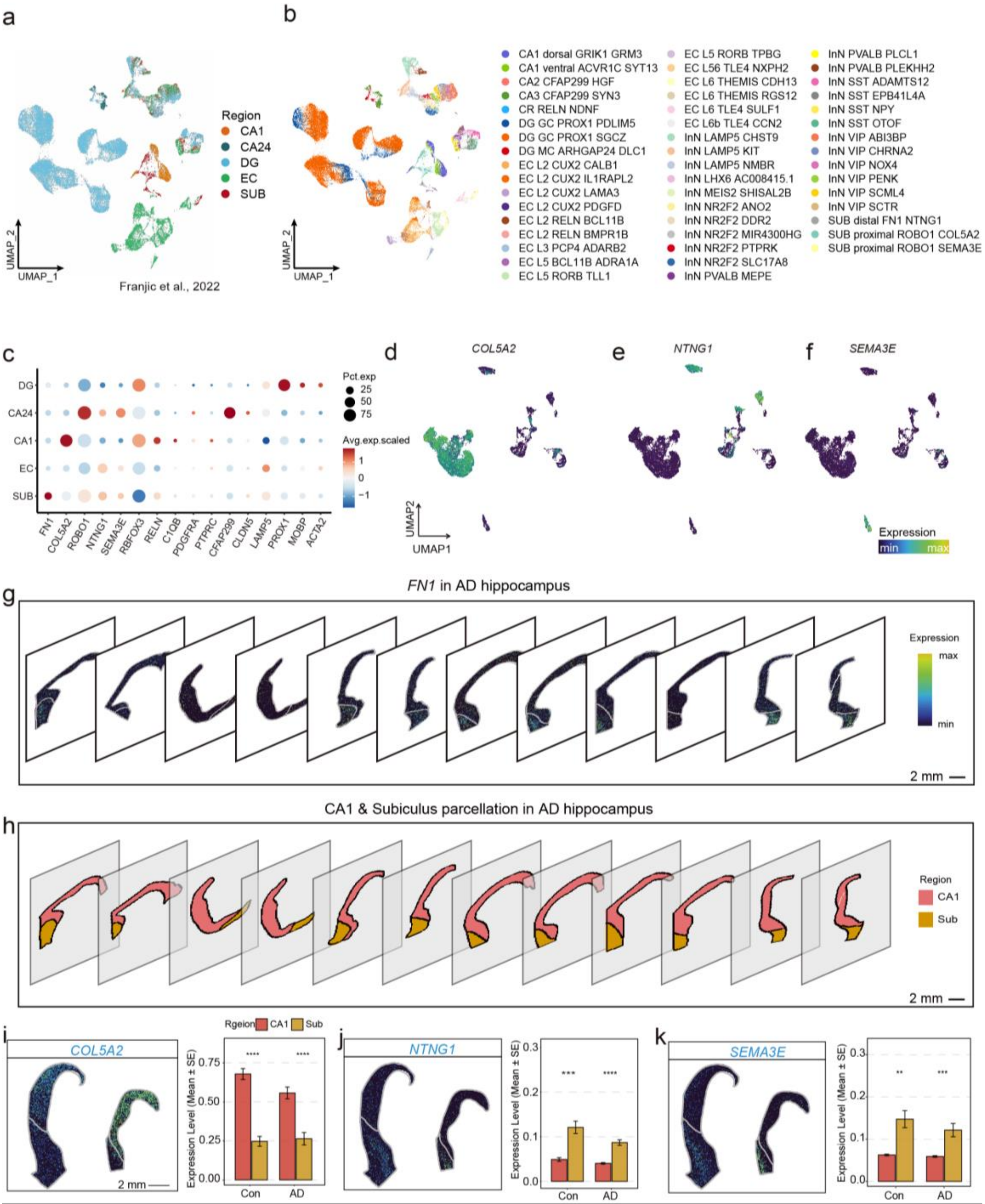

Extended Data Fig. 2| Expression of non-signature genes in public data and confirmation of hippocampal regions.

(**a-b**) UMAP visualization of snRNA dataset[24] from the human hippocampus colored by regions (**a**) and cell types (**b**). (**c**) Dot plot showing the expression of reported region-specific genes cross brain regions. (**d-f**) Expression of *COL5A2* (**c**), *NTNG1* (**e**) and *SEMA3E* (**f**) genes in the UMAP of CA1 and Sub regions. (**g**) Spatial expression of *FN1* gene in the AD hippocampal regions. (**h**) Parcellation of the Sub and CA1 regions in the AD hippocampal regions.

Characterization and cross-platform benchmarking of the Sub-specific molecular signature

After establishing the spatial demarcation of the Subiculum (Sub), we sought to define the definitive molecular signature that distinguishes this architecture from the adjacent CA1. Differential expression analysis revealed a suite of genes with highly enriched expression in Sub versus CA1 (**Fig. 3a**; **Supplementary Table 2**). Among these, we focused on three top markers: ETS Variant Transcription Factor 1 (*ETV1*), Proprotein Convertase Subtilisin/Kexin Type 1 (*PCSK1*), and Calcium/Calmodulin-Dependent Protein Kinase II Gamma (*CAMK2G*), which were robustly upregulated in the Sub.

We verified their regional specificity in the public snRNA-seq dataset[24], confirming that each is predominantly expressed by Subiculum neurons (**Fig. 3b**). *ETV1*, a transcription factor linked to synaptic plasticity, showed a patchy distribution corresponding to subicular subdomains[25]; *PCSK1*, involved in neuropeptide processing[26], and *CAMK2G*, a kinase regulating calcium signaling[27], were likewise concentrated in Subiculum. We further quantified *FN1* expression in spatial data, which confirmed *FN1*'s exclusive enrichment in Subiculum compared to CA1 (**Fig. 3c**). Similarly, spatial expression maps and spot-wise quantification for *ETV1*, *PCSK1*, and *CAMK2G* showed sharp enrichment at the CA1–Sub boundary and within Sub, reinforcing their specificity (**Fig. 3d**, **e**; **Extended Data Fig. 3a–e**).

To provide orthogonal validation, we performed RNAscope in situ hybridization for *FN1* and *ETV1*, which visibly localized both transcripts to the Sub in human hippocampal sections, mirroring the spatial transcriptomic data (**Fig. 3f, g**). We also leveraged a recent 10X Genomics spatial transcriptomics dataset of human hippocampus[28] for independent confirmation. Although the 10X chips were smaller and did not fully cover the CA1-Sub continuum, we identified sections containing both regions (**Fig. 3h**) and observed that *FN1* and *ETV1* were specifically expressed in the Subiculum, with little to no signal in adjacent CA1 (**Fig. 3i** and **j**). This cross-validation underscores the reliability of our markers and boundary demarcation. Moreover, by digitally partitioning multiple control hippocampi into CA1 vs. Sub regions, we examined expression gradients of key genes across the boundary (**Fig. 3k**, **Extended Data Fig. 3f** and **h**). We found that Sub-specific genes, especially *FN1* and *ETV1*, are virtually absent in CA1, then sharply upregulated at the CA1–Sub border, with levels increasing deeper into Subiculum. In contrast, *MPPED1*, a CA1-enriched but not CA1-exclusive marker, showed the opposite trend: high in proximal CA1 and tapering off across the boundary into Sub. Interestingly, *NTNG1* and *SEMA3E* also increased gradually along the Subiculum, suggesting they may serve as markers for proximal-distal subicular sublayers (**Fig. 3l**, **Extended Data Fig. 3g** and **i**). Finally, to explore functional distinctions, we performed disease-relevance enrichment using genes enriched in each region (focusing on control samples to avoid AD-related bias). The CA1 signature showed overlap with pathways associated

with intellectual disability, whereas the Subiculum signature was associated with synucleinopathies and dementia (**Fig. 3m**). These descriptive associations indicate that CA1 and the Subiculum possess distinct molecular profiles. While further study is required to determine if these signatures relate to specific disease vulnerabilities, this underscores our framework's utility in enabling anatomically grounded, region-resolved interrogation of brain disease.

Fig. 3

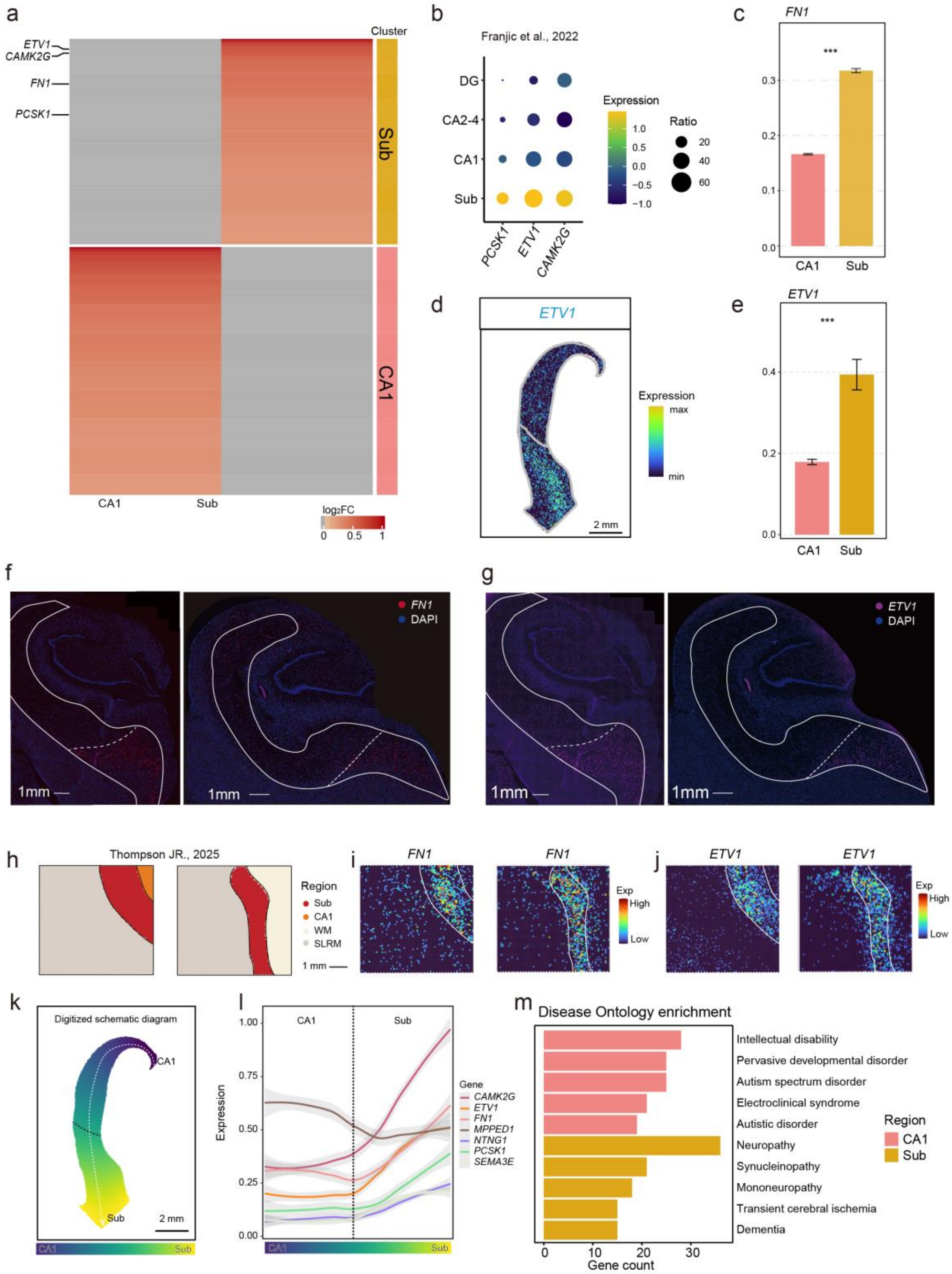

Fig. 3| Analysis of differences between Sub and CA1 regions.

(**a**) Heatmap showing average $\log_2$ (fold change (FC)) of region-specific expressing genes for each area. (**b**) Dot plot showing the marker genes of Sub region in public snRNA dataset. (**c**) Quantitative statistics of *FN1* gene in hippocampal regions. Difference between CA1 and Sub was examined by *t*-test with Holm's correction, *** $p < 0.001$. (**d**) Spatial expression of *ETV1* gene in hippocampal regions of Con individual. (**e**) Quantitative statistics of *ETV1* gene in hippocampal regions of Con

individuals. Difference between CA1 and Sub was examined by *t*-test with Holm's correction, *** $p < 0.001$. (**f**) Verification of *FN1* gene (red) and cells (blue) staining in Con (left) and AD (right) chips respectively by RNAscope. (**g**) Verification of *ETV1* gene (purple) and cells (blue) staining in Con (left) and AD (right) chips respectively by RNAscope. (**h**) area annotation of public spatial data from Thompson JR et al. 2025[28]. (**i**) The expression of *FN1* in public spatial data. (**j**) The expression of *ETV1* in public spatial data. (**k**) The digitized schematic diagram of CA1 and Sub brain regions. (**l**) The expression of marker genes statistically analyzed after digital stratification. (**m**) Disease Ontology (DO) terms analysis was enriched by Sub/CA1-specific genes.

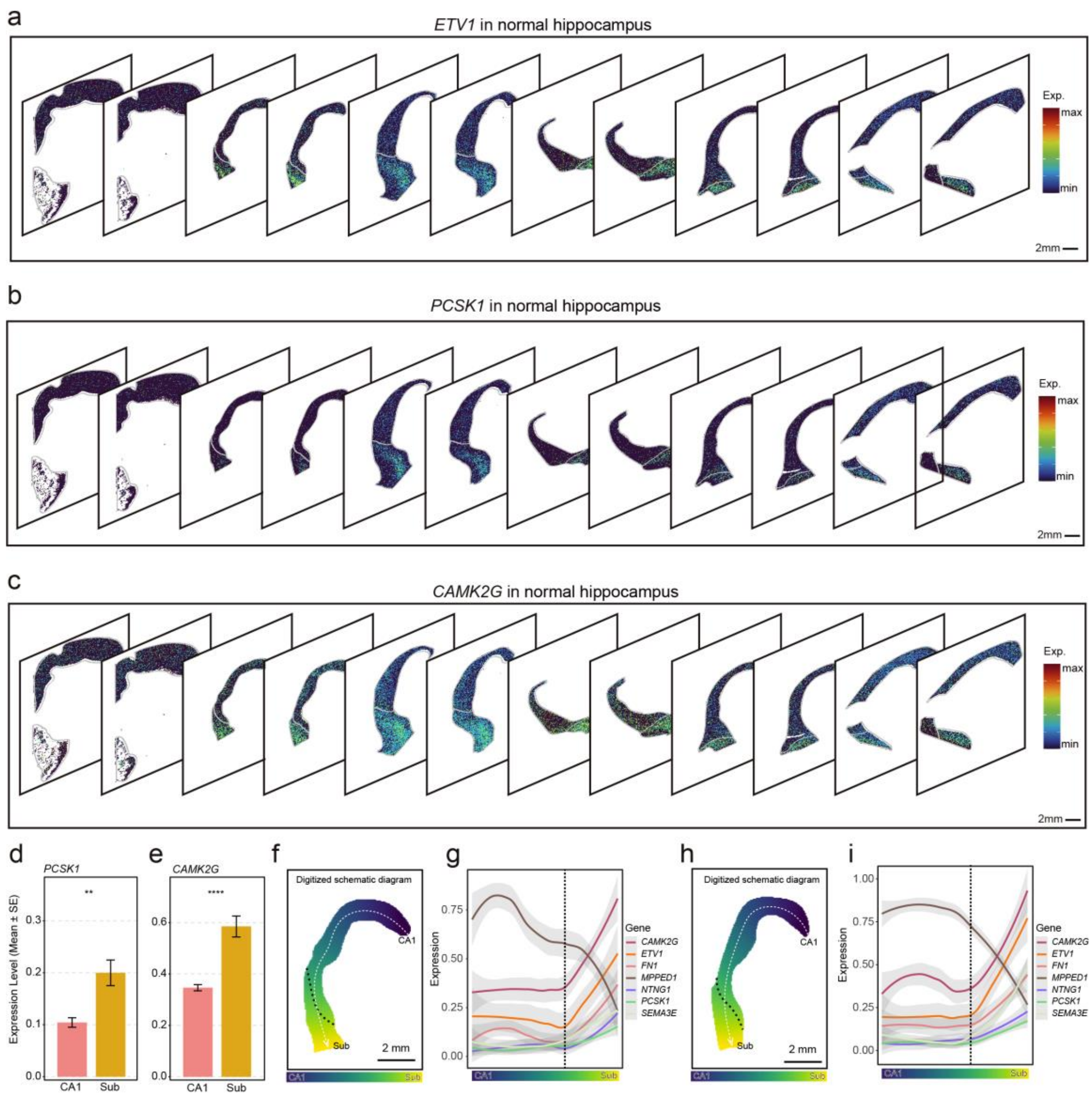

Extended Data Fig. 3| Spatial visualization of *PCSK1*, *ETV1* and *CAMK2G* genes in all samples

(**a-c**) Spatial expression of *ETV1* (**a**), *PCSK1* (**b**), and *CAMK2G* (**c**) genes in the Con hippocampal regions. Scale bar: 2 mm. (**d-e**) Quantitative statistics of *PCSK1* (**d**) and *CAMK2G* (**e**) in hippocampal regions of Con individuals. Difference between CA1 and Sub was examined by *t*-test with Holm's correction, **** $p < 0.0001$, ** $p < 0.01$. (**f** and **h**) The digitized schematic diagram of

CA1 and Sub regions. (**g** and **i**) The expression of specific genes in the CA1 and Sub brain regions after digitalization (**f** and **h**).

Application Case Study: High-resolution parcellation unmasks region-specific Metabolic-Functional Remodeling in AD

To demonstrate the functional utility of our molecularly anchored framework (**Fig. 3**), we interrogated region-resolved transcriptional alterations in Alzheimer's Disease (AD) across the newly defined CA1–Sub interface. By applying our precise parcellation pipeline, we identified 467 AD-associated differentially expressed genes (DEGs) across the CA1–Sub interface (278 upregulated and 189 downregulated; **Supplementary Table 3**), revealing distinct region-specific metabolic-functional biases that would otherwise be masked by conventional anatomical mapping. Among upregulated DEGs, 27 genes were shared between Sub and CA1 and were enriched for processes related to metal ion homeostasis and regulation of apoptosis. Region-biased signals were also evident: CA1-upregulated DEGs (n = 122) were enriched for hypoxia response and gliogenesis, consistent with glial activation, whereas Sub-upregulated DEGs (n = 129) showed strong enrichment for mitochondrial energy metabolism, including ATP metabolic processes and electron transport–coupled pathways[29] (**Fig. 4a** and **b**; **Extended Data Fig. 4a** and **b**). For example, *NDUFA4*, a mitochondria-associated gene linked to respiratory chain function, was markedly dysregulated in Sub in AD, supporting altered oxidative phosphorylation-related programs in this region[30] (**Fig. 4a** and **b**).

Downregulated DEGs in both regions converged on proteostasis-related pathways, while Sub-biased downregulated DEGs showed additional enrichment for synapse organization (**Fig. 4c** and **d**). To assess whether AD preferentially perturbs regional identity programs, we intersected region-specific marker sets with AD-related DEGs. In Sub, 58 Sub markers were downregulated in AD, whereas only three Sub markers (*SNCG*, *TUBB2B*, *RPS27*) were upregulated (**Fig. 4e**; **Supplementary Table 4**); the downregulated Sub markers were enriched for synaptic functional terms (**Fig. 4f**). Notably, *NDUFA4* was not a Sub identity marker, suggesting that AD-related transcriptional remodeling in Sub involves not only attenuation of region-specific programs but also induction of additional stress-associated processes. In CA1, 43 CA1-specific markers were all downregulated in AD (**Extended Data Fig. 4c**; **Supplementary Table 5**), with enrichment pointing to protein polymerization-related processes (**Extended Data Fig. 4d)**.

To capture coordinated network-level changes, we performed high-dimensional weighted gene co-expression network analysis (hdWGCNA)[17] using AD-associated DEGs and identified 10 co-expression modules (**Fig. 4g**; **Extended Data Fig. 4e**). Module eigengenes showed region-dependent expression patterns and functional annotations (**Fig. 4h**; **Extended Data Fig. 4f–i**). Sub-biased downregulated genes were enriched in modules associated with lipid handling/high-density

lipoprotein particle clearance[31], neuronal support function[32] and regulation of synaptic plasticity[22] (M2, M5, and M4; **Fig. 4g**; **Supplementary Table 6**). In contrast, Sub-biased upregulated genes were concentrated in an energy metabolism module (M8), enriched for ATP metabolic processes and electron transport–coupled pathways (**Fig. 4g–j**; **Supplementary Table 6**). Network topology further highlighted mitochondria-related hub genes, including *NDUFA4* and *CALM2*[30,33] (**Fig. 4k**). Collectively, these results serve as a functional validation of our spatial framework. Relative to CA1, the Sub region exhibits a more prominent induction of mitochondrial energy metabolism–related transcriptional networks in AD, complementing our boundary framework with a region-resolved view of disease-associated programs.

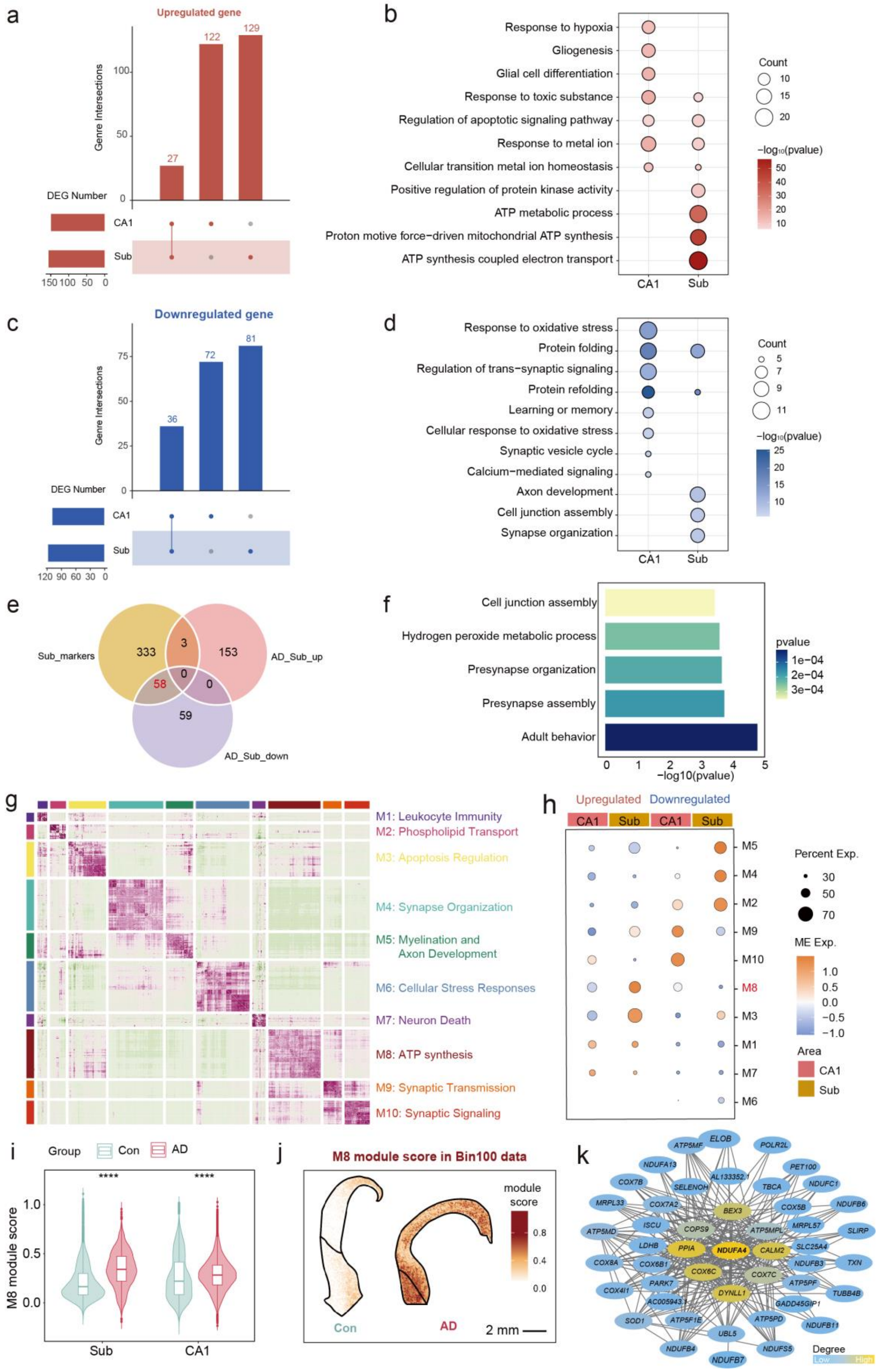

Fig. 4| Differential gene expression and co-expression network analysis in Sub and CA1 regions of AD using bin100 data

(**a-b**) The number of upregulated (**a**) and downregulated (**b**) DEGs across the Sub and CA1 regions in AD. (**c-d**) Dot plots showing the enriched GO terms for upregulated (**c**) and downregulated (**d**) DEGs in the Sub and CA1 regions, respectively. **(e)** The intersection of Sub-specific genes and the AD DEGs in the Sub region. **(f)** Pathway enrichment analysis Sub-specific genes downregulated in AD. (**g**) Heatmap showing ten modules of DEGs classified by hdWGCNA, with each correlated to distinct biological processes. (**h**) Dot plot showing the Module eigengenes (ME) for DEGs in Sub and CA1 regions within AD and Con. (**i**) Volin plot illustrating the M8 module gene scores between AD and Con within Sub and CA1 regions in all chips. Difference between AD and Con was examined by *t*-test with Holm's correction, **** $p < 0.0001$. (**j**) Spatial expression of M8 module gene scores in hippocampal regions of AD (right) and Con (left). Scale bar: 2 mm. (**k**) Co-expression network of Intersection of M8 module genes and upregulated genes in AD Sub region visualized using Cytoscape. Edges represent interactions, and node color indicates connectivity degree.

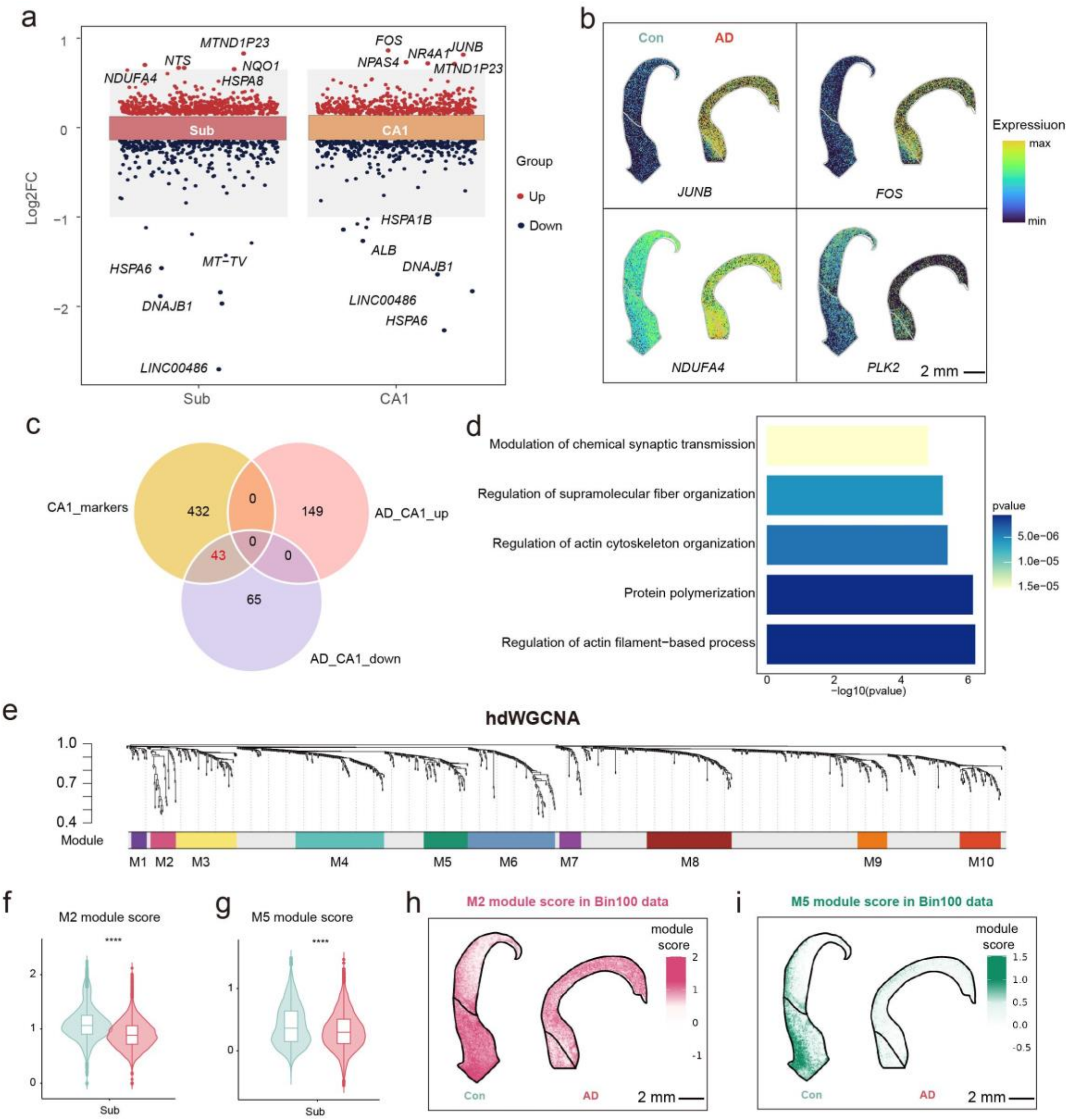

Extended Data Fig. 4| Spatial analysis of AD-linked DEGs and modules in Sub and CA1 regions

(**a**) The volcano plots showing the AD-associated DEGs in the Sub and CA1 regions. (**b**) Spatial expression of *JUNB*, *PLK2*, *NDUFA4*, *CALM2* genes between AD and Con group. (**c**) The intersection of Sub-specific genes and the AD DEGs in the Sub region. (**d**) Pathway enrichment analysis CA1-specific genes downregulated in AD. (**e**) Ten co-expression modules of DEGs clustered by hdWGCNA. (**f-g**) Volin plots illustrating the M2 and M5 module gene scores between AD and Con within Sub and CA1 regions. Difference between AD and Con was examined by *t*-test with Holm's correction, **** $p < 0.0001$. (**h-i**) Spatial distribution of M2 and M5 module gene scores of Con (left) and AD (right). Scale bar: 2 mm.

Multi-scale mapping identifies neuronal drivers of Sub-specific bioenergetic programs

To dissect the cellular contributors to the regional metabolic signature observed in the Sub, we performed brain region-specific alignment by mapping bin100 matrices to cellbin coordinates, yielding a spatially resolved single-cell transcriptomic atlas (**Fig. 5a**). The accuracy of our cell-type annotations, performed via the Spatial-ID method[15], was corroborated by high similarity with public snRNA-seq benchmarks (**Fig. 5b**).

Quantitative density analysis revealed a significant reduction in neuronal populations, particularly excitatory neurons, alongside an increased microglial density in both the Sub and CA1 regions of AD patients (**Fig. 5c**). In addition, cell-type-specific DEGs analysis showed different expression patterns between Sub and CA1 regions. In the CA1 region, the expression of DEGs across multiple cell types, such as Astro, Micro, OPC, and pericyte, were enrichment for pathways related to cellular cation homeostasis and axonal development (**Fig. 5d**, **f**). In contrast, in the Sub region, DEGs were predominantly upregulated in specific neuronal populations, most notably EX_Sub and IN_PVALB neurons, and were enriched for mitochondrial energy metabolism pathways including ATP metabolic processes, aerobic respiration, and oxidative phosphorylation (**Fig. 5d**, **f**).

To connect these cell-type signatures to our region-level co-expression network, we compared cell-type–specific DEGs with the hdWGCNA modules using Jaccard similarity. This analysis highlighted a strong overlap between the mitochondrial/energy module M8 and DEGs from EX_Sub and IN_PVALB neurons (**Fig. 5g**). Consistently, gene set scoring showed significant elevation of M8-associated expression programs in EX_Sub and IN_PVALB neurons in the AD Sub (**Fig. 5h-l**), with increased M8 scores in AD versus controls for both neuronal populations (**Fig. 5i**, **k**) and spatial visualization recapitulating these shifts at the tissue level (**Fig. 5j**, **l**). Finally, analysis of M8 hub genes identified *NDUFA4* as one of the most strongly upregulated electron transport chain–associated genes in AD EX_Sub neurons (**Fig. 5m**, **n**).

Collectively, these results demonstrate that the Sub-specific metabolic signature in AD is not a global tissue response but is preferentially engaged within specialized neuronal populations, a discovery enabled by our high-resolution parcellation framework.

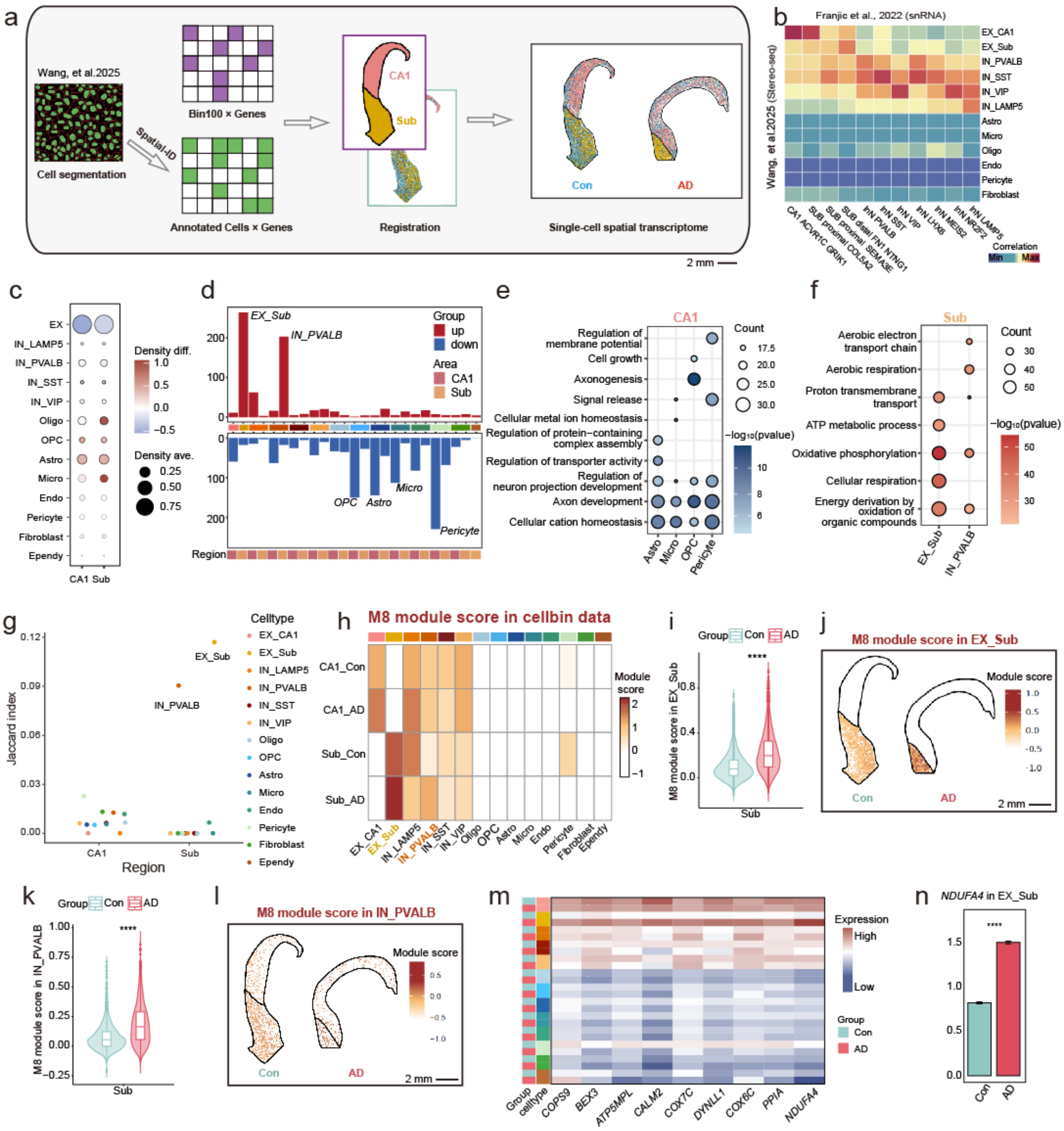

Fig. 5| Single-cell spatial transcriptome analysis revealed cell type-specific gene expression changes in the Sub and CA1 regions of AD

(**a**) The workflow illustrating the integration of bin100 and cellbin matrices to generate spatially resolved single-cell transcriptomic datasets with brain region annotations. (**b**) Pearson correlation of cell types between snRNA dataset from Franjic et al. (2022)[24] and our stereo-cell data. (**c**) Dot plot depicting cell type densities in the Sub and CA1 regions. The spot size represented the average density of each cell type for AD and Con. The spot color represented an increase (red) or decrease (blue) of cell density in AD. Density diff., Density difference. (**d**) Bar plot quantifying upregulated and downregulated DEGs across cell types in the AD group. (**e**) Dot plot showing the enriched GO

terms for downregulated DEGs of Astro, Micro, OPC and Pericyte in the CA1 region. (**f**) Dot plot showing the enriched GO terms for upregulated DEGs of EX_Sub and IN_PVALB neurons in the Sub region. (**g**) Dot plot showing the Jaccard similarity index of M8 module genes and DEGs of different cell types. (**h**) Heatmap of M8 module score across Sub and CA1 regions within the different cell types in AD and Con groups. (**i** and **k**) Violin plots showed the M8 module gene scores between AD and Con for EX_Sub (**i**) and IN_PVALB (**k**) cell types within the Sub region across all chips. Difference between AD and Con was examined by *t*-test with Holm's correction, **** $p < 0.0001$. (**j** and **l**) Spatial expression of M8 module gene scores in EX_Sub (**j**) and IN_PVALB neurons (**l**) of AD (right) and Con (left). Scale bar: 2 mm. (**m**) Heatmap of expression for hub genes (*COPS9*, *BEX3*, *ATP5MPL*, *CALM2*, *COX7C*, *DYNLL1*, *COX6C*, *PPIA*, *NDUFA4* genes) in the Sub region of different cell types. (**n**) Bar plot showing mean expression of *NDUFA4* in EX_Sub neurons of AD and Con groups. Difference between AD and Con was examined by *t*-test with Holm's correction, **** $p < 0.0001$.

Discussion

In this study, we established a molecularly anchored framework to delineate the Subiculum (Sub) from the CA1 in the human hippocampus by integrating high-definition spatial transcriptomics (Stereo-seq) with snRNA-seq. Our data-driven parcellation resolved the historically ambiguous CA1–Sub interface, transforming a subjective anatomical challenge into a verifiable molecular reference. By systematically benchmarking markers across modalities, we identified *FN1* as a stringent boundary marker and established a robust toolkit including *ETV1*, *PCSK1*, and *CAMK2G* for reproducible annotation in human hippocampal research.

A major contribution of this framework is its ability to address the "boundary-spanning" dilemma in neuropathology. Because the CA1–Sub transition is difficult to define via conventional anatomy, previous studies often discarded tissue spanning this interface to avoid misassignment, leading to a significant underuse of scarce post-mortem human material[34,35]. Our gene expression–based definition enables the inclusion of these transition zones in large-scale omics analyses, providing molecularly grounded regional assignment. This standardized approach not only increases the volume of analyzable data per specimen but also enhances the reproducibility of hippocampal subfield studies across different laboratories.

Using the molecular CA1–Sub boundary as a reference, we performed region-resolved comparisons in Alzheimer's disease (AD). These analyses suggested divergent transcriptional programs across regions, including stronger induction of mitochondrial energy metabolism–related signatures in Sub and stress/glial-associated responses in CA1. At the cell-resolved level, these regional patterns appeared to be preferentially engaged within specific neuronal populations in Sub. We present these findings as a "proof-of-concept" application of our framework, highlighting prioritized targets such as NDUFA4 for future mechanistic studies[36] into regional disease vulnerability rather than definitive causal mechanisms, with further functional studies required to clarify whether this response is compensatory or maladaptive.

While our spatially resolved single-cell approach significantly enhances regional specificity, several constraints inherent to human brain banking should be noted[37]. The reliance on post-mortem tissue precludes the tracking of dynamic disease progression and may limit the detection of early-stage molecular changes[38]. Furthermore, although our sample size was carefully matched for key covariates such as age and sex to ensure high inter-group comparability, it may not fully capture the broad spectrum of inter-individual variability across all AD stages. The co-expression modules and hub genes identified, such as the M8 module, provide valuable correlative insights but require further experimental validation to establish definitive causal relationships. Future prospective

studies with larger, stage-stratified cohorts will be essential to further validate the generalizability of this molecular reference.

The limited sample size may not fully capture inter-individual variability. It also precludes detailed stratification of AD cases by disease stage. These constraints stem from the inherent nature of brain bank-donated samples. Donor availability is often unpredictable. Furthermore, spatial transcriptomics imposes strict technical requirements for tissue integrity[39]. Notably, these 12 samples represent all available specimens in the brain bank with matched key covariates. We prioritized age-matching to ensure high inter-group comparability. Future studies should prospectively collect larger cohorts stratified by AD stage. This will validate our findings and enhance their generalizability.

In conclusion, this study provides a high-resolution molecular atlas and a reproducible parcellation pipeline for the human CA1–Sub interface. By identifying definitive boundary markers and unmasking region-specific bioenergetic remodeling in AD, our findings provide a foundational resource for the fields of neurology and spatial pathology.

Acknowledgments

This work was supported by Key Program of the National Natural Science Foundation of China (32530027 and 82501681), the National Science and Technology Innovation 2030 Major Program (2021ZD0204400). We thank BGI for hosting various datasets from this study, DCS Cloud (https://cloud.stomics.tech/) for providing the computational resources and software support necessary for this study.

Author contributions

LF.W designed the research; YY.L and YZ.H analyzed the sequencing data and wrote the manuscript. LF.W revised the manuscript. L.H, YR.W, P.W, CY.H, CY.H, QY.T, LJ.Z, X.X and LQ.L contributed to unpublished reagents/analytic tools/advices. All authors read and approved the manuscript for submission.

Data Availability

The datasets supporting the findings of this study are available from public repositories. The high-resolution spatial transcriptomic (Stereo-seq) data have been deposited in the CNGB Nucleotide Sequence Archive (CNSA) of the China National GeneBank DataBase (CNGBdb) under accession code CNP0005077 (https://db.cngb.org/search/project/CNP0005077/).

The single-nucleus RNA sequencing (snRNA-seq) data utilized in this study were obtained from the Gene Expression Omnibus (GEO) under accession number GSE186538, which includes transcriptomic profiles microdissected from multiple human hippocampal and parahippocampal regions, including the dentate gyrus (DG), CA2–4, CA1, Subiculum (Sub), and entorhinal cortex (EC).

Competing interests

Employees of BGI have stock holdings in BGI. All other authors declare no competing interests.

Supplementary information

Correspondence and requests for materials should be addressed to wanglifang@genomics.cn

References

1 Iglesias, J. E. *et al.* A computational atlas of the hippocampal formation using ex vivo, ultra-high resolution MRI: Application to adaptive segmentation of in vivo MRI. *NeuroImage* **115**, 117-137, doi:10.1016/j.neuroimage.2015.04.042 (2015).

2 Tosches, M. A. *et al.* Evolution of pallium, hippocampus, and cortical cell types revealed by single-cell transcriptomics in reptiles. *Science* **360**, 881-888 (2018).

3 Lisman, J. *et al.* Viewpoints: how the hippocampus contributes to memory, navigation and cognition. *Nature neuroscience* **20**, 1434-1447 (2017).

4 Donato, F. *et al.* The Ontogeny of Hippocampus-Dependent Memories. *The Journal of neuroscience : the official journal of the Society for Neuroscience* **41**, 920-926, doi:10.1523/jneurosci.1651-20.2020 (2021).

5 Berdugo-Vega, G., Dhingra, S. & Calegari, F. Sharpening the blades of the dentate gyrus: how adult-born neurons differentially modulate diverse aspects of hippocampal learning and memory. *The EMBO journal* **42**, e113524, doi:10.15252/embj.2023113524 (2023).

6 O'Mara, S. M., Commins, S., Anderson, M. & Gigg, J. The subiculum: a review of form, physiology and function. *Progress in neurobiology* **64**, 129-155, doi:10.1016/s0301-0082(00)00054-x (2001).

7 Sun, Y. *et al.* CA1-projecting subiculum neurons facilitate object-place learning. *Nature neuroscience* **22**, 1857-1870, doi:10.1038/s41593-019-0496-y (2019).

8 Mizuseki, K. & Kitanishi, T. J. C. O. i. N. Oscillation-coordinated, noise-resistant information distribution via the subiculum. *Current Opinion in Neurobiology* **75**, 102556 (2022).

9 Huxter, J. R., Senior, T. J., Allen, K. & Csicsvari, J. J. N. n. Theta phase–specific codes for two-dimensional position, trajectory and heading in the hippocampus. *Nature neuroscience* **11**, 587-594 (2008).

10 Kitanishi, T., Umaba, R. & Mizuseki, K. Robust information routing by dorsal subiculum neurons. *Sci Adv* **7**, doi:10.1126/sciadv.abf1913 (2021).

11 Romani, A. *et al.* Community-based reconstruction and simulation of a full-scale model of the rat hippocampus CA1 region. *PLoS biology* **22**, e3002861, doi:10.1371/journal.pbio.3002861 (2024).

12 Gong, C. *et al.* SAW: an efficient and accurate data analysis workflow for Stereo-seq spatial transcriptomics. *GigaByte* **2024**, 1-12 (2024).

13 Zhang, B. *et al.* Generating single-cell gene expression profiles for high-resolution spatial transcriptomics based on cell boundary images. *GigaByte* **2024**, 1-13 (2024).

14 Zhao, E. *et al.* Spatial transcriptomics at subspot resolution with BayesSpace. *Nature biotechnology* **39**, 1375-1384, doi:10.1038/s41587-021-00935-2 (2021).

15 Shen, R. *et al.* Spatial-ID: a cell typing method for spatially resolved transcriptomics via transfer learning and spatial embedding. *Nature communications* **13**, 7640, doi:10.1038/s41467-022-35288-0 (2022).

16 Yu, G., Wang, L. G., Han, Y. & He, Q. Y. clusterProfiler: an R package for comparing biological themes among gene clusters. *Omics : a journal of integrative biology* **16**, 284-287, doi:10.1089/omi.2011.0118 (2012).

17 Morabito, S., Reese, F., Rahimzadeh, N., Miyoshi, E. & Swarup, V. hdWGCNA identifies co-expression networks in high-dimensional transcriptomics data. *Cell reports methods* **3**, 100498, doi:10.1016/j.crmeth.2023.100498 (2023).

18 Shannon, P. *et al.* Cytoscape: a software environment for integrated models of biomolecular interaction networks. *Genome research* **13**, 2498-2504, doi:10.1101/gr.1239303 (2003).

19 Chin, C. H. *et al.* cytoHubba: identifying hub objects and sub-networks from complex interactome. *BMC systems biology* **8 Suppl 4**, S11, doi:10.1186/1752-0509-8-s4-s11 (2014).

20 Rosenblum, E. W., Williams, E. M., Champion, S. N., Frosch, M. P. & Augustinack, J. C. The prosubiculum in the human hippocampus: A rostrocaudal, feature-driven, and systematic approach. *The Journal of comparative neurology* **532**, e25604, doi:10.1002/cne.25604 (2024).

21 Adler, D. H. *et al.* Histology-derived volumetric annotation of the human hippocampal subfields in postmortem MRI. *NeuroImage* **84**, 505-523, doi:10.1016/j.neuroimage.2013.08.067 (2014).

22 Wang, P. *et al.* Molecular pathways and diagnosis in spatially resolved Alzheimer's hippocampal atlas. *Neuron* **113**, 2123-2140.e2129, doi:10.1016/j.neuron.2025.03.002 (2025).

23 El Hayek, L. *et al.* Disruption of the autism gene and chromatin regulator KDM5A alters hippocampal cell identity. *Sci Adv* **9**, eadi0074, doi:10.1126/sciadv.adi0074 (2023).

24 Franjic, D. *et al.* Transcriptomic taxonomy and neurogenic trajectories of adult human, macaque, and pig hippocampal and entorhinal cells. *Neuron* **110**, 452-469.e414, doi:10.1016/j.neuron.2021.10.036 (2022).

25 Tao, Y. *et al.* Erbin regulates NRG1 signaling and myelination. *Proceedings of the National Academy of Sciences of the United States of America* **106**, 9477-9482, doi:10.1073/pnas.0901844106 (2009).

26 Podvin, S., Wojnicz, A. & Hook, V. Human brain gene expression profiles of the cathepsin V and cathepsin L cysteine proteases, with the PC1/3 and PC2 serine proteases, involved in neuropeptide production. *Heliyon* **4**, e00673, doi:10.1016/j.heliyon.2018.e00673 (2018).

27 Rigter, P. M. F. *et al.* Role of CAMK2D in neurodevelopment and associated conditions. *American journal of human genetics* **111**, 364-382, doi:10.1016/j.ajhg.2023.12.016 (2024).

28 Thompson, J. R. *et al.* An integrated single-nucleus and spatial transcriptomics atlas reveals the molecular landscape of the human hippocampus. *Nature neuroscience* **28**, 1990-2004, doi:10.1038/s41593-025-02022-0 (2025).

29 Cadonic, C., Sabbir, M. G. & Albensi, B. C. Mechanisms of Mitochondrial Dysfunction in Alzheimer's Disease. *Molecular neurobiology* **53**, 6078-6090, doi:10.1007/s12035-015-9515-5 (2016).

30 Adav, S. S., Park, J. E. & Sze, S. K. Quantitative profiling brain proteomes revealed mitochondrial dysfunction in Alzheimer's disease. *Molecular brain* **12**, 8, doi:10.1186/s13041-019-0430-y (2019).

31 Hong, B. V. *et al.* High-Density Lipoprotein Changes in Alzheimer's Disease Are APOE Genotype-Specific. *Biomedicines* **10**, doi:10.3390/biomedicines10071495 (2022).

32 Fleeman, R. M., Kuhn, M. K., Chan, D. C. & Proctor, E. A. Apolipoprotein E ε4 modulates astrocyte neuronal support functions in the presence of amyloid-β. *Journal of neurochemistry* **165**, 536-549, doi:10.1111/jnc.15781 (2023).

33 Azarnia Tehran, D. *et al.* Selective endocytosis of Ca(2+)-permeable AMPARs by the Alzheimer's disease risk factor CALM bidirectionally controls synaptic plasticity. *Sci Adv* **8**, eabl5032, doi:10.1126/sciadv.abl5032 (2022).

34 González-Arnay, E. *et al.* Immunohistochemical field parcellation of the human hippocampus along its antero-posterior axis. *Brain structure & function* **229**, 359-385, doi:10.1007/s00429-023-02725-9 (2024).

35 Maynard, K. R. *et al.* Transcriptome-scale spatial gene expression in the human dorsolateral prefrontal cortex. *Nature neuroscience* **24**, 425-436 (2021).

36 Pitceathly, R. D. *et al.* NDUFA4 mutations underlie dysfunction of a cytochrome c oxidase subunit linked to human neurological disease. *Cell reports* **3**, 1795-1805 (2013).

37 Williams, C. G., Lee, H. J., Asatsuma, T., Vento-Tormo, R. & Haque, A. J. G. m. An introduction to spatial transcriptomics for biomedical research. *Genome medicine* **14**, 68 (2022).

38 Hyman, B. T. *et al.* National Institute on Aging–Alzheimer's Association guidelines for the neuropathologic assessment of Alzheimer's disease. *Alzheimer's & Dementia* **8**, 1-13 (2012).

39 Rao, A., Barkley, D., França, G. S. & Yanai, I. J. N. Exploring tissue architecture using spatial transcriptomics. *Nature* **596**, 211-220 (2021).

Supplementary Table

Click here to access/download
Supplementary Material
05_SupplementaryTables.xlsx
