# Supplementary material for: A molecularly anchored spatial transcriptomic framework for precise CA1–subiculum parcellation and region-resolved analysis in Alzheimer’s disease
Source: Gigascience. 2026 Sep 17;15:giag094. doi: 10.1093/gigascience/giag094 (PMC13674357; doi:10.1093/gigascience/giag094)
Supplement: Supplementary file 3 [file giag094_giga-d-26-00145_revision_1.pdf]

GigaScience

A Molecularly Anchored Spatial Transcriptomic Framework for Precise CA1–Subiculum Parcellation and Region-Resolved Analysis in Alzheimer's Disease

--Manuscript Draft--

**Manuscript Number:**	GIGA-D-26-00145R1
**Full Title:**	A Molecularly Anchored Spatial Transcriptomic Framework for Precise CA1–Subiculum Parcellation and Region-Resolved Analysis in Alzheimer's Disease
**Article Type:**	Research
**Funding Information:**	Key Program of the National Natural Science Foundation of China (32530027) — Prof. Jing Zhang, Professor Lifang Wang<br>National Science and Technology Innovation 2030 Major Program (2021ZD0204400) — Doc. Lei Han
**Abstract:**	Background: The precise molecular delineation of the interface between the Subiculum (Sub) and cornu ammonis 1 (CA1) is a challenge in hippocampal research, as conventional cytoarchitectural boundaries are often ambiguous and limit reproducible regional annotation. Here, we developed a molecularly anchored spatial transcriptomic framework to define CA1-Sub regional identities using high-definition spatial transcriptomics (Stereo-seq) and single-nucleus RNA sequencing (snRNA-seq) references.<br>Findings: Using a human hippocampal Stereo-seq dataset from 12 donors, we established a data-driven parcellation framework that defines reproducible molecular features distinguishing CA1 and Sub while capturing the transition between these regions. FN1 was identified as a Sub-enriched marker in a subset of EX_Sub and, together with ETV1 and additional regional markers, enabled molecular assignment of CA1 and Sub identities across datasets. The Sub association of FN1 and ETV1 was further supported by human 10X Genomics spatial transcriptomics, mouse in situ hybridization data, and a mouse spatial transcriptomic dataset. Applying this framework to Alzheimer's disease (AD) tissues revealed region-specific transcriptional alterations across CA1 and Sub, including enrichment of mitochondrial energy metabolism-related transcripts in the Sub, suggesting exploratory transcriptional associations of altered metabolic function.<br>Conclusions: This study provides a molecularly anchored framework for human CA1–Sub parcellation that complements conventional annotation. By defining regional molecular states while preserving the biological continuum across CA1–Sub interface, this approach enables more consistent regional analysis of human hippocampus tissue across donors, datasets, and disease conditions.
**Corresponding Author:**	Lifang Wang<br>BGI Research, Hangzhou<br>CHINA
**Corresponding Author Secondary Information:**	
**Corresponding Author's Institution:**	BGI Research, Hangzhou
**Corresponding Author's Secondary Institution:**	
**First Author:**	Yuyang Liu
**First Author Secondary Information:**	
**Order of Authors:**	Yuyang Liu
	Youzhe He
	Yanrong Wei
	Pan Wang
	Chunyu Huang
	Ouyuan Tao

	Langjian Zhu
	Xun Xu
	Longqi Liu
	Lei Han
	Jing Zhang
	Lifang Wang
**Order of Authors Secondary Information:**	

Response to Reviewers:

Please note that this text field does not support embedded figures. We have therefore uploaded two separate files in the Files section: a “Cover Letter” summarizing the major revisions and a complete “Response to Reviewers” containing our point-by-point responses and all rebuttal figures. A text-only version of our responses is provided below.

Reviewer reports:
Reviewer #1: Manuscript #GIGA-D-26-00145 "A Molecularly Anchored Spatial Transcriptomic Framework for Precise CA1-Subiculum Parcellation and Region-esolved Analysis in Alzheimer's Disease" by Y. Liu et al., leverages publicly available data obtained by spatial transcriptomics (Stereo-seq) and snRNA-seq from Wang et al., 2025 and Franjic et al., 2022 to establish boundaries of key hippocampal regions, namely the CA1 sector and the subiculum. Discerning the anatomic boundaries between the CA1 sector and subiculum are notoriously difficult, especially within the postmortem human brain. The authors utilize several advanced bioinformatics techniques and AI supported programs to identify specific genetic markers (e.g., FN1 with support from ETV1, PCSK1, and CAMK2G) to delineate CA1-subiculum boundaries, and present a viable panel of potential markers for demarcation. This mapping heuristic may be useful in the context of normal subjects as well as inform neurodegenerative disorders including Alzheimer's disease (AD). There is a lot of information to unpack in this study! The authors are urged to reconsider the organization of the manuscript for readership. Below are major and minor considerations to improve an interesting and potentially impactful manuscript.
Response: We sincerely thank reviewer for the thorough and constructive evaluation of our manuscript, and for recognizing the potential significance of this work in delineating hippocampal boundaries and advancing our understanding of Alzheimer's disease. We have carefully addressed all comments raised, and our point-by-point responses are provided below.

Major
1. Manuscript stewardship. The entire manuscript would benefit from a hard copyedit from a native English speaking collaborator who can help shape the narrative. Reliance on online/AI grammar is not recommended as the issues of organization and higher order integration require collaborative assistance.
Response: We thank the reviewer for this valuable suggestion. In response, we have enlisted the help of a professional academic editing service to conduct a thorough hard copyedit of the entire manuscript, strictly avoiding reliance on AI tools as advised. Beyond grammatical corrections, we undertook a comprehensive revision focused on improving the scientific narrative and higher order integration. Specifically, we reorganized the presentation of the results, eliminated redundant statements, and strengthened the logical transitions between sections. We believe these revisions have substantially improved the clarity, coherence, and accessibility of the manuscript, and we would be pleased to make any further adjustments considered necessary.

2. Tissue sample access. Stereo-seq data was obtained from Wang et al., 2025. RNAscope validation appears to have been conducted on a subset of tissues. It is unclear if the authors had direct access to the tissues or if they are from another cohort. This needs to be specified for the assays. Moreover, the exact relationship with Wang et al., 2025 needs to be stated in the Methods and whether (or not) this study is a reanalysis of previously acquired data or a follow-up of shared tissues.
Response: We thank the reviewer for this valuable comment. We agree that provenance of the spatial transcriptomic data and the relationship between the present study and Wang et al. (2025) should be clearly specified.

Powered by Editorial Manager® and ProduXion Manager® from Aries Systems Corporation

(1) All Stereo-seq spatial transcriptomic data analyzed in this study were derived from the previously published human hippocampal Stereo-seq dataset reported by Wang et al. (2025). No new Stereo-seq sequencing experiments or additional spatial transcriptomic data generation were performed in the present study. Instead, this work represents a secondary computational reanalysis of the previously acquired spatial transcriptomic resource using a molecularly anchored framework for CA1-Sub parcellation and region-resolved analysis of AD. The original dataset consisted of a larger collection of human hippocampal Stereo-seq sections. For the present study, we selected 24 tissue sections from 12 specimens (6 AD and 6 Control) based on predefined criteria, including tissue integrity, anatomical preservation, and the presence of an identifiable CA1-Sub region required for downstream parcellation analyses. The inclusion status and corresponding selection criteria for each chip have now been clarified in Supplementary Table 1. We have added this information to the Methods section (Pages 30-31, lines 591-613) and the Result section (Page 5, lines 94-104).
(2) The RNAscope validation experiments were performed using cryopreserved adjacent tissue sections from the same human hippocampal tissue cohort described in Wang et al. (2025). During the original tissue preparation process Stereo-seq profiling, adjacent sections from the same tissue blocks and anatomical regions were preserved. These matched sections were subsequently used for RNAscope validation. This approach ensures precise spatial and anatomical matching with the sequencing data. Therefore, the RNAscope experiments in this study represent an orthogonal tissue-level validation using anatomically matched human sections from the same cohort, rather than a validation performed on a separate external patient cohort. We have clarified this relationship in the revised Methods section and specified that the present study combines secondary computational analysis of previously published Stereo-seq data with additional targeted molecular validation using adjacent cryopreserved tissue sections (Pages 35, lines 747-754).

3. Tissue handling. The authors need to state specifically how tissues were handled and prepared including tissue fixation procedures, size and number of sections used for snRNA-seq and RNAscope.
Response: We thank the reviewer for pointing out the need for clarification regarding our tissue handling and preparation protocols. Followed the reviewers' recommendation, we have updated the detailed descriptions of RNAscope validation experiments in the Methods section, including the tissue fixation procedures, size and number of sections used for RNAscope (Pages 35, lines 747-754).
We have also clarified that the snRNA-seq data analyzed in this study were not newly generated by us. Instead, the snRNA-seq dataset was retrieved from the public GEO database under accession number GSE186538 and was used for secondary analysis. Therefore, no new tissue handling, tissue sectioning, or fixation procedures for snRNA-seq were performed in the present study. The relevant descriptions have been added to the Methods section (Page 30, lines 570-576; Pages 32-33, lines 662-679).

4. Tissue selection criteria. An explanation of how tissues were included (and excluded) is warranted. The Reviewer suggests a Supplementary image panel of what is acceptable or not acceptable tissue quality for postmortem hippocampal and subiculum sections.
Response: We thank the reviewer for this constructive suggestion regarding tissue selection and quality-control criteria. We agree that a clear description of tissue inclusion and exclusion is important for interpreting the CA1–Sub-specific analyses. During the anatomical annotation phase, our primary inclusion criterion for CA1–subiculum-specific analyses was whether the tissue section contained an identifiable and analyzable subiculum (Sub) region. Tissue sections that did not encompass an analyzable Sub region were therefore excluded from downstream CA1–Sub analyses. Only sections containing both the relevant hippocampal anatomy and an identifiable Sub region were retained for subsequent CA1–Sub-specific analyses (Rebuttal Fig 1a). In addition to this anatomy-based filtering, we further evaluated chip-level heterogeneity using principal component analysis (PCA). During this quality-control step, two AD chips, AD3.1 and AD3.2, showed marked separation from the remaining chips in the PCA space, indicating substantial heterogeneity relative to the rest of the cohort (Rebuttal Fig 1b). To avoid introducing chip-level outlier effects into the downstream spatial and transcriptomic analyses, AD3.1 and AD3.2 were excluded from the final CA1–Sub analyses.

In response to the reviewer's suggestion, we have expanded Supplementary Table 1, "Summary metrics from the CNBB cohort" to include tissue-selection and quality-control information. Specifically, we added a column indicating whether the Sub region was present and analyzable for each chip, as well as the final inclusion/exclusion status and the corresponding exclusion reason. This provides a transparent overview of how tissues were included or excluded across the cohort. We also added Supplementary Table 2, which summarizes the group-level comparisons of demographic, clinical, and tissue-quality variables between the AD and control cohorts, together with the corresponding statistical tests and p values. The text describing these tissue-selection and quality-control criteria has also been updated in the Methods section (Pages 30-31, lines 595-613).

Rebuttal Fig. 1: Tissue inclusion and chip-level quality control for CA1–Sub analyses.
(a) Anatomy-based tissue selection. Sections were retained only if they contained an identifiable and analyzable Sub region suitable for CA1–Sub comparison. Sections lacking an analyzable Sub region were excluded from downstream CA1–Sub-specific analyses.
(b) PCA-based chip-level quality control. Principal component analysis showed that AD3.1 and AD3.2 were markedly separated from the remaining chips, indicating substantial global transcriptomic heterogeneity. These two chips were excluded from the final CA1–Sub analyses to reduce chip-level outlier effects.

5. AI assistance verification. Was manual validation of AI-generated segmentation performed? Greater details regarding QA/QC is strongly recommended to indicate validity of the AI techniques used.
Response: Thank you for raising this important point. We have added additional quality-control and validation analyses to support the reliability of the cell segmentation, cell-type annotation, and downstream spatial transcriptomic quantification. These analyses are now included in Extended Data Fig. 6.
First, we evaluated the cell segmentation performance using three representative image fields. Manual inspection-based benchmarking showed a high detection rate, with an average Recall of 0.92 ± 0.02 and F1 score of 0.82 ± 0.02 across the three fields (Extended Data Fig. 6a, lines 409-410). This indicates that the segmentation procedure successfully detected -the majority of cells. The main remaining limitation was occasional over-segmentation in densely packed or clustered regions, which we now explicitly acknowledge.
Second, we added a canonical marker-gene heatmap to validate cell-type annotation. Major neuronal, glial, vascular, immune and ependymal populations showed expected marker enrichment, including excitatory neuronal markers, inhibitory neuronal markers, oligodendrocyte/OPC markers, astrocyte, microglial, endothelial, pericyte and ependymal markers. This supports the biological consistency of the annotated cell classes (Extended Data Fig. 6b, lines 410-412).
Third, we added chip-level quality-control plots comparing UMI counts and detected gene numbers across control and AD samples. These QC distributions showed comparable overall sequencing/capture quality across chips and conditions, supporting that the observed biological differences are unlikely to be driven by gross technical imbalance (Extended Data Fig. 6c-d, lines 412-414).
Together, these additional analyses strengthen the technical validation of the segmentation, annotation and data quality. We have added these results the relevant Results text (Page 20, lines 353-359).

6. RNAscope. The RNAscope approach requires greater detail (e.g., case demographics, technical strategy) along with the method of comparison to spatially derived data from Wang et al., 2025.
Response: We thank the reviewer for the opportunity to clarify the technical strategy and demographic pairing of our validation cohort.
Technical Strategy & Sample Pairing: The tissue sections used for RNAscope validation were cryopreserved sections obtained from corresponding anatomical regions within the same hippocampal tissue blocks used for Stereo-seq profiling in Wang et al. (2025). This tight spatial coupling ensures that our validation assays directly reflect the transcriptomic microenvironments captured in the discovery dataset. We have updated the relevant Methods text (Pages 35, lines 747-754).
Case Demographics & Chip Metadata: In alignment with the reviewer's comment, we have integrated the detailed case demographics, clinical parameters from Wang et al.,

Powered by Editorial Manager® and ProduXion Manager® from Aries Systems Corporation

2025 into Supplementary Table 1.

7. Discussion. Despite the large amount of data and supportive validation, the Discussion is skeletal. The authors are urged to rewrite the Discussion to reflect the impact of the current boundary delineation findings as well as reframe postmortem human tissue based studies attempting to define the CA1-subiculum continuum in the context of existing literature.
Response: We thank the reviewer for this constructive suggestion. We agree that the original Discussion did not sufficiently emphasize the broader significance of our CA1–Sub parcellation framework or place the findings in the context of postmortem human hippocampal studies.
We have therefore substantially rewritten and expanded the Discussion. In the revised manuscript, we now discuss why the CA1–Sub interface is difficult to define in postmortem human tissue, how gradual cytoarchitectural and molecular transitions complicate traditional anatomical annotation, and how our molecularly anchored framework provides a reproducible strategy for region-resolved analysis. We also clarify that the CA1–Sub interface should be interpreted as a gradual molecular transition zone and that our annotations represent operational, molecularly informed parcellations rather than evidence for a sharp anatomical border. In addition, we now discuss the impact of this framework for postmortem tissue studies, including its potential to reduce regional misclassification, improve reproducibility, and increase the analyzable use of scarce human hippocampal specimens. We also expanded the AD-related discussion to present the disease findings as a proof-of-concept application and to acknowledge limitations related to sample size, Braak-stage heterogeneity, APOE genotype, and postmortem tissue constraints.
Overall, the revised Discussion now better reflects the impact, context, and limitations of the study. The relevant content has been updated in the Discussion section of the revised manuscript (Pages 25-29, lines 419–558).

Minor
1. Please restructure the manuscript for conventional readership by presenting approaches in the Methods, findings in the Results, and interpretations in the Discussion. The original submission has these sections dispersed throughout the text.
Response: We thank the reviewer for this helpful suggestion. We agree that the original manuscript would benefit from a more conventional and reader-friendly organization.
In the revised manuscript, we have reorganized both the content and the overall structure of the manuscript. Specifically, we adjusted the section order so that the manuscript now presents the Results first, followed by the Discussion and then the Methods, in accordance with the journal format. Within this revised structure, methodological details, including data sources, preprocessing steps, segmentation, annotation, statistical analyses, and validation procedures, have been consolidated into the Methods section. The Results section has been revised to focus on the main findings and supporting analyses, whereas broader interpretation, comparison with existing literature, implications, and limitations have been moved to the Discussion section.

2 . Relocate Source code from the Methods to the Data Availability section.
Response: We thank the reviewer for this suggestion. We have relocated the source code information from the Methods section to the Data Availability section in the revised manuscript. The revised Data Availability section now includes the project name, project homepage, license, operating system, programming language, package management information, and hardware requirements (Pages 37, lines 791-804). This change improves compliance with the journal format and separates code availability information from the methodological descriptions.

3. Are images of the sections that the authors evaluated publicly available? Please upload them to a repository such as www.synapse.org.
Response: We thank the reviewer for this helpful suggestion. The RNAscope images evaluated in this study, together with the in situ hybridization images showing Fn1 and Etv1 expression in the mouse brain obtained from the Allen Mouse Brain Atlas, have now been made publicly available through our GitHub repository (https://github.com/L-Yuyang/2025_ST-Human-Hippocampus-Boundary/tree/main/Validation_Images). The original data sources and relevant image information from the Allen Mouse Brain Atlas

Powered by Editorial Manager® and ProduXion Manager® from Aries Systems Corporation

are clearly indicated in the repository. We have also added the repository link to the revised manuscript to facilitate public access and independent evaluation (Page 37, lines 796-800).

4. There is an enormous amount of spatial and sequence data. Please curate genelists for ease of readership and consider making them available as Supplementary material.
Response: We thank the reviewer for this helpful suggestion. We agree that curated gene lists are important for improving readability, transparency, and reuse of the spatial and sequencing analyses. In the revised manuscript, we have curated the major gene lists generated and used in this study and made them available as Supplementary Tables.
Specifically, we have added Supplementary Table 3, entitled "Marker genes for the four BayesSpace-defined clusters within the MPPED1-defined CA1–subiculum region" which provides the marker genes supporting the BayesSpace-based molecular parcellation of the CA1–Sub region. We have also added Supplementary Table 8, entitled "Gene lists of hdWGCNA co-expression modules identified from AD-associated differentially expressed genes" which provides the gene composition of the hdWGCNA modules used for network-level interpretation of AD-associated changes.
Together with the existing supplementary materials, the revised manuscript now includes nine Supplementary Tables. These tables are referenced in the relevant Results sections and provide curated donor information, cluster markers, region-associated genes, and network/module gene lists to help readers navigate and reuse the large spatial and sequence datasets analyzed in this study.

5. Little information is given to the AD cohort. Since AD hallmark pathology is present in the CA1 and subiculum, how is this expected to impact the ability to delineate these boundaries?
Response: We thank the reviewer for raising this important point.
In the revised manuscript, we have clarified the information of AD cohort (Page 5, lines 100-104; Page 31, lines 611-615) and summarized the available demographic and pathological metadata in Supplemental Table 1.
In the original manuscript, CA1- and Sub-enriched genes were identified from control samples to establish the baseline molecular features of the CA-Sub interface. This molecular framework was substantially applied to AD samples to evaluate whether AD-associated pathology affects the ability to delineate this regional boundary. We found that the expression patterns of representative markers were largely preserved in AD samples (Extended Data Fig. 2g, line 179; Fig. 3f-g, lines 245-247). To further evaluate whether AD-associated pathology affects the robustness of this boundary, we performed additional differential expression analyses separately in control and AD samples. The results showed that the regional expression direction of the core CA1/Sub markers was largely preserved in both groups. In particular, the Sub-enriched markers identified in our original analysis, including FN1, ETV1, PCSK1, and CAMK2G, retained more highly expressed in Sub than in CA1 in AD samples (Rebuttal Fig. 3). These results suggest that the major molecular features underlying CA1–Sub regional identity remain detectable despite AD-associated transcriptional alternations. Together. these analyses support the robustness of our molecularly informed CA1–Sub parcellation framework in AD tissues, while we acknowledge that AD pathology may influence the magnitude of some regional transcriptional signals.

Rebuttal Fig. 3: Consistency of CA1–Sub regional marker patterns in control and AD tissues.
Scatter plot comparing the log2 fold change of Sub versus CA1 expression in control samples and AD samples. The strong positive correlation indicates that the regional enrichment direction of CA1- and Sub-associated genes is largely preserved across diagnostic groups, with representative Sub-enriched markers, including FN1, ETV1, PCSK1, and CAMK2G, remaining consistently enriched in the Sub region.

Reviewer #2: Summary
This study addresses the persistent challenge of reliably defining the CA1-subiculum boundary in hippocampal research. To resolve inconsistencies, the authors employ complementary methods Stereo-seq spatial transcriptomics and single-nucleus RNA-seq on 12 human donors (6 with Alzheimer's disease and 6 controls). They identify FN1 as a molecular marker of the subiculum, with support from ETV1, PCSK1, and CAMK2G, and validate these findings using RNAscope and an independent dataset.

Applying this molecular boundary to Alzheimer's tissue reveals region-specific changes, most notably increased mitochondrial and energy-metabolism gene expression in the subiculum. The study is interesting and potentially valuable, though several issues remain to be addressed.
Response: We thank the reviewer for the thoughtful summary and positive assessment of our study. We appreciate the recognition of the potential value of defining the CA1–Sub boundary using complementary spatial transcriptomic, single-nucleus, RNAscope, and external validation approaches. We have carefully considered the reviewer's comments and have addressed each point in detail below.

Major
1. Clarify the origin of the data. The paper reads as if a new 12-donor dataset was generated, but the Stereo-seq, snRNA-seq, and 10X data all come from earlier studies (Wang 2025; Franjic 2022; Thompson 2025); only the RNAscope is new. State this plainly and explain how the study relates to Wang 2025.
Response: We thank the reviewer for highlighting the need to clarify the origin of the data. We have revised the manuscript to state explicitly that the Stereo-seq, snRNA-seq, and 10X datasets were all obtained from previously published studies, namely Wang et al., 2025, Franjic et al., 2022, and Thompson et al., 2025, respectively. No new Stereo-seq, snRNA-seq, or 10X sequencing datasets were generated in this study. The only newly generated experimental data were from RNAscope validation (Page 30, lines 562-589; Page 35, lines 747-750).
We have also clarified the relationship between the RNAscope validation samples and the Stereo-seq dataset reported by Wang et al. (2025). The tissue sections used for RNAscope validation were cryopreserved sections obtained from corresponding anatomical regions within the same hippocampal tissue blocks used for Stereo-seq profiling in Wang et al. (2025). They were therefore anatomically matched to the Stereo-seq sections, although they were not necessarily serially adjacent sections. This sampling strategy provided close spatial and anatomical correspondence between the RNAscope validation assays and the transcriptomic regions analyzed in the discovery dataset (Page 35, lines 747-750).

2. Strengthen the statistical analysis. Testing a large number of bins or cells does not compensate for the small cohort of 6 Alzheimer's disease donors and 6 control donors, as the donor is the appropriate unit of analysis. Therefore, the main results should be recomputed at the donor level using suitable models, such as pseudobulk or mixed-effects approaches.
Response: We thank the reviewer for this important and constructive comment. As suggested, we have therefore recomputed the key analyses at the donor level using donor-level mean expression and pseudobulk-based approaches. First, for the regional enrichment analysis of MPPED1 in Fig. 1b, we recalculated MPPED1 expression at the donor level. Specifically, MPPED1 expression was averaged within CA1 and within the other hippocampal regions for each chip, and donor-level paired comparisons were then performed separately in the control and Alzheimer's disease groups. The revised donor-level analysis confirmed that MPPED1 remained enriched in CA1 compared with other hippocampal regions in both groups (Fig. 1b, lines 122-127). Second, we further validated the expression patterns of FN1, ETV1, PCSK1, and CAMK2G by calculating their average expression in CA1 and Sub for each donor/chip-level sample. The donor-level regional mean expression analysis showed that these genes were consistently higher in the Sub than in CA1, supporting their use as Sub-enriched markers (Fig. 3c and e, lines 240-245; Extended Data Fig. 3d-e, lines 257-259). The corresponding procedures for donor-level aggregation and statistical analysis have also been described in the revised Methods section (Page 34, lines 716-721).

3. Provide direct evidence that FN1 marks neurons. Although FN1 is primarily expressed in the extracellular matrix and vasculature bordering the subiculum, the observed elevated FN1 signal may reflect non-neuronal contamination. Therefore, the authors should provide direct evidence that FN1 marks subicular neurons.
Response: We thank the reviewer for this important comment. In the published snRNA-seq dataset analyzed in our study (Franjic et al., 2022; PMID: 34798047, was selectively expressed in a subicular excitatory neuronal population annotated as the FN1-positive subtype. In addition, Fei et al. (2026; PMID: 41984531) provided another anatomical evidence supporting the expression of FN1 in Sub projection neurons. TB-555, a retrograde neuronal tracer that labels neurons projecting to the injection site,

whose injection into the entorhinal cortex showed that FN1 expression was predominantly associated with retrogradely labeled neurons in the Sub.
We also performed additional validation using the CellBin-level spatial transcriptomic data in our study. At the cell-bin resolution, we examined FN1 expression across annotated cell types and brain regions. This analysis showed that FN1 was highly expressed in neuronal cells within the Sub region. In parallel, ETV1, another marker identified in our analysis, also showed elevated expression in Sub neuronal populations (Rebuttal Fig. 4).
Importantly, we have revised the wording throughout the manuscript to avoid overgeneralizing FN1 as a pan-neuronal marker. Instead, we now describe FN1 as a marker enriched in a subset of Sub neurons and use it together with ETV1 and other markers as part of a molecular panel for CA1–Sub boundary delineation. We also acknowledge that FN1 has known extracellular matrix and vascular associations (PMID: 38598053), and therefore its interpretation is strengthened by cell-type-resolved CellBin analysis and independent anatomical evidence (Page 2, lines 36-37; Page 7, lines 150-151; Page 25, lines 424-426).
,

Rebuttal Fig. 4: CellBin-level expression of FN1 and ETV1 across CA1 and Sub cell types.
Dot plot showing scaled expression and percentage of expressing cells for FN1 and ETV1 across annotated CellBin cell types in the Sub and CA1. FN1 and ETV1 showed higher expression in EX_Sub than in the corresponding CA1 neuronal and non-neuronal populations, supporting their association with subicular neuronal populations.

4.No evidence is provided that the Alzheimer's and control groups were matched for post-mortem interval, RNA quality, or cause of death. Several leading Alzheimer's disease genes (FOS, JUNB, NR4A, NPAS4) are stress- and death-response genes, so bin-level results may confound cell loss with real expression changes. These factors weaken the metabolic findings.
Response: We thank the reviewer for raising this important concern. As suggested, we have expanded Supplementary Table 1 to include all available donor-level metadata on post-mortem interval (PMI), RNA integrity number (RIN), Braak stage, and Thal stage, and have summarized the corresponding group-level comparisons in updated Supplementary Table 2. Using donors as independent statistical units, we did not detect statistically significant differences between AD and control groups in PMI (Welch's t-test, $p = 0.36$) or RIN ($p = 0.15$). However, given the cohort size, we do not interpret these non-significant results as formal evidence of equivalence. Complete cause-of-death information was not available for all donors; therefore, residual confounding related to agonal state or terminal events cannot be excluded.
To assess whether reduced EX_CA1 abundance contributed to the observed expression differences, we first quantified EX_CA1 density in the AD and control groups. EX_CA1 density was lower in the AD group than in the control group (Rebuttal Fig. 5a; Wilcoxon test, $p < 0.05$), indicating that differences in neuronal abundance could potentially influence the bin-level comparison. We therefore performed a density-matched subsampling sensitivity analysis in which EX_CA1-assigned cells from the control group were down-sampled to approximate the EX_CA1 density distribution observed in the AD group. After downsampling, we did not detect a statistically significant group difference in EX_CA1 density (Rebuttal Fig. 5b; Wilcoxon test, $p > 0.05$), and the neuronal module scores were also similar between the density-matched AD and control groups (Rebuttal Fig. 5c; Wilcoxon test, $p > 0.05$). We then repeated the differential-expression analysis using these density-matched EX_CA1 cells. Among the four activity- and stress-responsive genes highlighted by the reviewer, JUNB retained the same direction of change and remained significant according to our predefined statistical criteria, whereas FOS, NR4A1, and NPAS4 no longer met those criteria (Rebuttal Fig. 5d). Because subsampling reduces the number of observations and therefore statistical power, the loss of significance for these three genes should not be interpreted as evidence that their original differences were caused solely by neuronal loss. Rather, this sensitivity analysis indicates that the JUNB signal was more robust to density-matched subsampling, whereas the evidence for FOS, NR4A1, and NPAS4 was less stable.
We agree that neither density-matched downsampling nor cell-type-restricted analysis can fully distinguish AD-associated transcriptional responses from responses related to neuronal stress. degeneration, agonal state, or other unmeasured donor-level factors.

Powered by Editorial Manager® and ProduXion Manager® from Aries Systems Corporation

We have therefore revised the Result and Discussion to avoid presenting these genes as definitive AD-specific alterations. We have also moderated the interpretation of the associated metabolic findings and now describe them as exploratory transcriptional associations rather than direct evidence of altered metabolic function (Page 2, line 43; Page 16, lines 293-296; Page 27, lines 516-519).

Rebuttal Fig. 5: Density-matched analysis of EX_CA1 cellbins reduces the potential confounding effect of neuronal loss on transcriptional differences between AD and Con.
(a) Comparison of EX_CA1 cell-bin density between the Alzheimer's disease (AD) and control groups before downsampling. EX_CA1 density was significantly higher in the control group than in the AD group. (b) Comparison of EX_CA1 cell-bin density after downsampling the control group to match the density distribution observed in AD. (c) Comparison of neuronal module scores between the density-matched AD and control EX_CA1 cell bins. (d) Differential-expression analysis of density-matched EX_CA1 cell bins between the AD and control groups. Among the stress- and death-response genes highlighted by the reviewer, JUNB remained significantly differentially expressed.

5. Resolve the conflicting boundary description. While graded markers and proximal/distal neuron subtypes suggest a gradual transition, the text describes a sharp boundary. This contradiction should be addressed, and strong claims about boundary sharpness should be tempered accordingly.
Response: We thank the reviewer for pointing out this important inconsistency. We have therefore revised the manuscript to avoid overstatement of boundary sharpness. Specifically, we have replaced descriptions such as "sharp boundary" or "clear-cut boundary" with more accurate terms, including "gradual transition", "molecular transition zone" and "operationally defined CA1-Sub interface". We now clarify that the purpose of our framework is not to impose an artificial sharp border, but to use spatially graded molecular markers to define a reproducible and biologically informed transition zone between CA1 and the Sub. We have also revised the Discussion to explicitly state that proximal/distal neuronal subtypes and graded marker expression are consistent with a continuum model of the CA1-Sub interface. Accordingly, the boundary annotations in this study should be interpreted as molecularly informed operational parcellations for region-resolved analysis, rather than as evidence for an abrupt anatomical border. The relevant content has been updated in revised manuscript (Page 25, lines 446-447; Page 26, lines 473-474; Page 10, lines 197-198; Page 11, lines 222-223; Page 26, lines 468-469; Page 28, lines 552-554).

Minor
6. Validation is limited: RNAscope was performed for only two genes, assessed visually with minimal sampling, and 10X validation is incomplete. The legend of Extended Data Fig. 1 reads "Con," while the figure is titled "AD"; this requires correction.
Response: We thank the reviewer for this critical comment. We agree that the original validation was limited in both marker breadth and quantitative sampling. To strengthen the validation, we added quantitative analysis of the RNAscope images for two representative markers FN1 and ETV1. Both the analyzed control section and the analyzed AD section showed higher FN1 and ETV1 signals in the Sub than in CA1 (Extended Data Fig. 3f–g, lines 259-261). We have added the complete image-sampling and quantification procedures to the Methods (Page 35, lines 762-775).
In addition, we have expanded the analysis of the independent 10X spatial transcriptomics transcriptomic dataset from Thompson et al. 2025. We included all available sections containing both CA1 and Sub according to the anatomical annotations, comprising 4 sections from 3 donors. To avoid treating individual spatial spots as independent biological replicates, gene expression was summarized at the section level within each anatomical region, and paired Sub–CA1 expression differences were calculated for FN1 and ETV1. Both genes showed a consistent Sub-enriched expression pattern in 4/4 of the evaluable sections (Extended Data Fig. 3h-j, lines 261-264). Since only two sections contained the complete CA1–Sub continuum, we now describe this analysis as supportive external cross-platform validation rather than comprehensive validation of the entire boundary (Page 30, lines 577-578).
Given the limited number of human hippocampal sections available for RNAscope validation, we additionally examined publicly available in situ hybridization data from

Powered by Editorial Manager® and ProduXion Manager® from Aries Systems Corporation

the Allen Mouse Brain Atlas. Across multiple mouse brain sections and independent image series, both Fn1 and Etv1 showed prominent expression in the Sub region (Extended Data Fig. 4a-b, lines 268-271). In addition, analysis of two publicly available mouse hippocampal spatial transcriptomic sections generated by Han et al. (2025) showed that Fn1 and Etv1 consistently exhibited higher expression in the anatomically annotated Sub than in neighboring hippocampal regions (Extended Data Fig. 4c–e, lines 271-274). These publicly available anatomical data provide independent, cross-species support for the association of FN1 and ETV1 with the Sub, while we acknowledge that they do not substitute for direct validation in additional human samples.
Finally, we revised the Results and Discussion to avoid overstatement. Specifically, we now describe the RNAscope, Allen Mouse Brain Atlas, and 10X Genomics analyses as complementary, orthogonal, and supportive validation of representative Sub-enriched markers, rather than as exhaustive validation of all candidate genes or definitive validation of the complete CA1-Sub boundary. RNAscope provides direct validation in human tissue, the Allen Mouse Brain Atlas offers independent cross-species anatomical support, and the external 10X dataset provides cross-platform confirmation in human spatial transcriptomic data (Page 27, lines 500-504).
We rechecked the sample annotation in Extended Data Fig. 1. The incorrect “Con” label in the legend has been corrected to “AD” to match the corresponding figure panel and sample identity (Page 7, line 138).

7. Donor information is incomplete: APOE genotype, sex, age, and post-mortem interval are not reported, and the Alzheimer's disease cases span a broad pathological range (Braak III-V) within a small cohort.
Response: We thank the reviewer for pointing out this important issue. In the revised manuscript, we have added detailed donor-level information, including age, sex, post-mortem interval (PMI), APOE genotype, diagnosis, and Braak stage, to Supplementary Table 1. We have also added Supplementary Table 2, which summarizes the group-level comparisons of demographic, clinical, and tissue-quality variables between the AD and control cohorts, together with the corresponding statistical tests and p values. The control cases showed absent to low Braak pathology, ranging from not detected to Braak stage II, whereas the AD cases ranged from Braak stage III to V, with most AD cases classified as Braak stage IV. The relevant description has also been added to the Methods section (Pages 31, lines 611-615). We agree that the AD cohort spans a pathological range within a relatively small sample size. We have therefore revised the manuscript to interpret AD-associated findings more cautiously and now explicitly acknowledge that Braak-stage heterogeneity may contribute to inter-donor variability (Page 28, lines 535-540). Future studies with larger cohorts and more balanced pathological staging will be needed to further assess how AD stage, APOE genotype, and other donor-level variables influence CA1–Sub boundary delineation and region-resolved disease signatures.

8. Inaccurate method description. The manuscript describes hdWGCNA as characterizing gene correlation patterns in microarray samples. This describes the original WGCNA; hdWGCNA was specifically designed for high-dimensional single-cell and spatial transcriptomic data, which is the data type used here. Please clarify.
Response: We thank the reviewer for pointing out this inaccurate description. We agree that the previous wording incorrectly referred to microarray samples, which describes the original WGCNA framework rather than hdWGCNA. This was a wording error in the manuscript. In the present study, we used hdWGCNA, which is designed for high-dimensional single-cell and spatial transcriptomic datasets. We have revised the Methods section to accurately describe hdWGCNA as a framework for identifying gene co-expression modules in high-dimensional single-cell/spatial transcriptomic data. The incorrect reference to microarray samples has been removed from the revised manuscript (Page 34, lines 730-736).

Editor comments:

The manuscript would benefit from a hard copyedit by a native English speaker or a professional language editing service to improve fluency and clarity.
Thank you for this valuable suggestion. In response, have enlisted the help of a professional academic editing service to conduct a thorough hard copyedit of the entire manuscript, strictly avoiding reliance on AI tools as advised. Beyond grammatical

	corrections, we undertook a comprehensive revision focused on improving the scientific narrative and higher order integration. Specifically, we reorganized the presentation of the results, eliminated redundant statements, and strengthened the logical transitions between sections. We believe these revisions have substantially improved the clarity, coherence, and accessibility of the manuscript, and we would be pleased to make any further adjustments considered necessary.
Additional Information:	
Question	Response
Are you submitting this manuscript to a special series or article collection?	No
**Experimental design and statistics**<br><br>Full details of the experimental design and statistical methods used should be given in the Methods section, as detailed in our Minimum Standards Reporting Checklist. Information essential to interpreting the data presented should be made available in the figure legends.<br><br>Have you included all the information requested in your manuscript?	Yes
**Resources**<br><br>A description of all resources used, including antibodies, cell lines, animals and software tools, with enough information to allow them to be uniquely identified, should be included in the Methods section. Authors are strongly encouraged to cite Research Resource Identifiers (RRIDs) for antibodies, model organisms and tools, where possible.<br><br>Have you included the information requested as detailed in our Minimum Standards Reporting Checklist?	Yes
**Availability of data and materials**<br><br>All datasets and code on which the conclusions of the paper rely must be either included in your submission or deposited in publicly available repositories (where available and ethically	Yes

appropriate), referencing such data using a unique identifier in the references and in the “Availability of Data and Materials” section of your manuscript.<br><br>Have you have met the above requirement as detailed in our Minimum Standards Reporting Checklist?	
GigaScience has policies and guidelines in place for the use of generative AI-writing tools such as ChatGPT. If you have used such writing tools to assist with writing the manuscript this must be declared and cited in the text. Authors should not list AI-writing tools and other AI-assisted technologies as an author or co-author and should acknowledge that they are fully responsible for text generated or refined by AI-writing tools.<p><br><br>A summary of use (particularly in the introduction or among methods) needs to be included at the end of the paper, and the outputs should also be included as a supplementary file hosted in GigaDB or other open repositories. Please read our guidelines for more information. <p><br><br>By submitting to GigaScience, you are aware of the journal's AI-writing tools policy, and if you have declared use of such tools below, you have acknowledged this where appropriate in your manuscript and have made a summary of use and outputs available. <p><br><b>AI-assisted writing tools have been used in the preparation of this manuscript?	No

Main text

Click here to access/download;Manuscript;02_Sub_Manu_GigaScience_260

A Molecularly Anchored Spatial Transcriptomic Framework for Precise CA1–Subiculum Parcellation and Region-Resolved Analysis in Alzheimer's Disease

Yuyang Liu[1,2†], Youzhe He[1,2†], Yanrong Wei[1,2], Pan Wang[3], Chunyu Huang[1,2], Quyuan Tao[1,2], Langjian Zhu[2], Xun Xu[1,4], Longqi Liu[1,6], Shiping Liu[1,6], Lei Han[4,5*], Jing Zhang[3*], Lifang Wang[2,6,7*]

Author affiliations:

[1] College of Life Sciences, University of Chinese Academy of Sciences, Beijing 100049, China.

[2] BGI Research, Hangzhou 310030, China.

[3] Department of Pathology, The First Affiliated Hospital, Zhejiang University School of Medicine, Hangzhou, Zhejiang 310002, China.

[4] BGI Research, Shenzhen 518083, China.

[5] Key Laboratory of Brain Cell Mapping of Zhejiang Province, Hangzhou 310030, China

[6] Key Laboratory of Spatial Omics of Zhejiang Province, BGI Research, Hangzhou 310030, China.

[7] Lead contact

[†]These authors contributed equally to this work.

*Correspondence: wanglifang@genomics.cn (L.W.), jzhang1989@zju.edu.cn (J.Z.) hanlei2@genomics.cn (L.H.).

Abstract

Background: The precise molecular delineation of the interface between the Subiculum (Sub) and cornu ammonis 1 (CA1) is a challenge in hippocampal research, as conventional cytoarchitectural boundaries are often ambiguous and limit reproducible regional annotation. Here, we developed a molecularly anchored spatial transcriptomic framework to define CA1-Sub regional identities using high-definition spatial transcriptomics (Stereo-seq) and single-nucleus RNA sequencing (snRNA-seq) references.

Findings: Using a human hippocampal Stereo-seq dataset from 12 donors, we established a data-driven parcellation framework that defines reproducible molecular features distinguishing CA1 and Sub while capturing the transition between these regions. *FN1* was identified as a Sub-enriched marker in a subset of EX_Sub and, together with *ETV1* and additional regional markers, enabled molecular assignment of CA1 and Sub identities across datasets. The Sub association of *FN1* and *ETV1* was further supported by human 10X Genomics spatial transcriptomics, mouse *in situ* hybridization data, and a mouse spatial transcriptomic dataset. Applying this framework to Alzheimer's disease (AD) tissues revealed region-specific transcriptional alterations across CA1 and Sub, including enrichment of mitochondrial energy metabolism-related transcripts in the Sub, suggesting exploratory transcriptional associations of altered metabolic function.

Conclusions: This study provides a molecularly anchored framework for human CA1–Sub parcellation that complements conventional annotation. By defining regional molecular states while preserving the biological continuum across CA1–Sub interface, this approach enables more consistent regional analysis of human hippocampus tissue across donors, datasets, and disease conditions.

Introduction

The hippocampus is a highly conserved structure composed of distinct anatomical regions, including the dentate gyrus (DG), cornu ammonis (CA1–CA4), and the Sub[1]. These regions are highly conserved during evolution[2], and form critical neural circuits essential for cognitive functions such as memory consolidation, spatial navigation, and emotional regulation[3]. As the primary output gateway of the hippocampal formation, the Sub is positioned between the CA1 region and the entorhinal cortex[4,5]. It serves as a vital relay station, receiving convergent inputs from CA1 and various cortical areas while transmitting processed information to downstream cortical and thalamic structures[6-11]. This anatomical positioning renders the Sub an indispensable hub for information flow within and beyond the hippocampal system, playing a pivotal role in hippocampal-dependent learning and memory.

Despite these functional differences, the precise anatomical and functional boundary between Sub and CA1 remains poorly defined. Traditional histological markers often fail to capture the molecular transitions at this interface, leaving the precise anatomical-functional boundary poorly defined and hindering our understanding of region-specific contributions to hippocampal circuitry[12].

Precise spatial delineation is paramount for deciphering how the hippocampal formation segregates complex cognitive processing. While traditional transcriptomic methods provide deep cellular insights, they often lack the spatial context and architectural resolution required to resolve these fine-scale anatomical boundaries in the human brain. This lack of a standardized molecular reference prevents researchers from accurately attributing pathological changes, such as those seen in AD, to specific hippocampal subfields, potentially masking region-specific disease mechanisms[13,14].

Spatial and spatiotemporal transcriptomic technologies have been increasingly applied to reconstruct tissue architecture and organ-level molecular organization across developmental and disease contexts[15]. In this study, we present a high-definition molecularly anchored framework for human CA1–Sub parcellation. By integrating high-definition spatial transcriptomics (Stereo-seq) with single-nucleus RNA sequencing (snRNA-seq), we established a definitive molecular reference at near-cellular resolution. We report a validated marker panel, headlined by *FN1* and *ETV1*, which provides a reproducible toolkit for subfield annotation. This framework enables the CA1–Sub interface to be examined as a molecularly continuous yet regionally organized structure, rather than as a boundary defined solely by conventional histological landmarks. It also provides a standardized reference for comparing regional transcriptional profiles across individuals, platforms, and disease states. By applying this framework to Alzheimer’s disease samples, we further demonstrate its utility for resolving region-associated transcriptional heterogeneity while preserving anatomical context. These analyses are intended to provide a spatially grounded basis for future studies of

hippocampal subfield vulnerability, circuit organization, and disease-associated molecular remodeling.

Results

A Data-driven Spatial Framework Resolves the Ambiguous CA1–Subiculum Boundary

For decades, the precise border between the Subiculum (Sub) and CA1 regions of the hippocampus has remained poorly defined by classical anatomy[16,17]. We addressed this long-standing challenge by developing a high-resolution molecularly anchored parcellation framework. To establish a molecularly anchored framework for precise CA1–Sub parcellation, we performed a secondary analysis of a previously published human hippocampal Stereo-seq spatial transcriptomic dataset (Stereo-seq)[18]. From the original spatial transcriptomic resource, we selected 12 specimens (6 AD and 6 Control) comprising 24 tissue sections based on tissue integrity, anatomical coverage, and the availability of an identifiable CA1–Sub region for downstream parcellation analyses (**Fig. 1a**, **Supplementary Table 1-2**). The selected samples included patients with moderate-to-advanced AD (Braak III–V, with abundant Aβ deposition) and age- and sex-matched controls from individuals without dementia and with absent to low levels of AD neuropathology. Two independent tissue sections were available per donor in the original dataset, providing a robust spatial transcriptomic resource for secondary analysis in the present study.

To define the putative CA1 territory, we first leveraged metallophosphoesterase domain containing 1 (*MPPED1*) as a known CA1 marker, observing its stable expression across all samples (**Fig. 1b** and **c**, **Extended Data Fig. 1a**)[19]. Based on *MPPED1* expression[18], we extracted the broad CA1 region from each section (**Fig. 1a**). While this initial demarcation aligned with traditional landmarks, unsupervised clustering (BayesSpace) within the *MPPED1*-defined CA1 unveiled two transcriptionally distinct domains (**Fig. 1d**, **Extended Data Fig. 1b**)[20]. This data-driven parcellation indicates that tissue annotated as a single anatomical field by conventional landmarks contains reproducible molecular heterogeneity, suggesting the presence of a latent boundary at the Sub–CA1 interface.

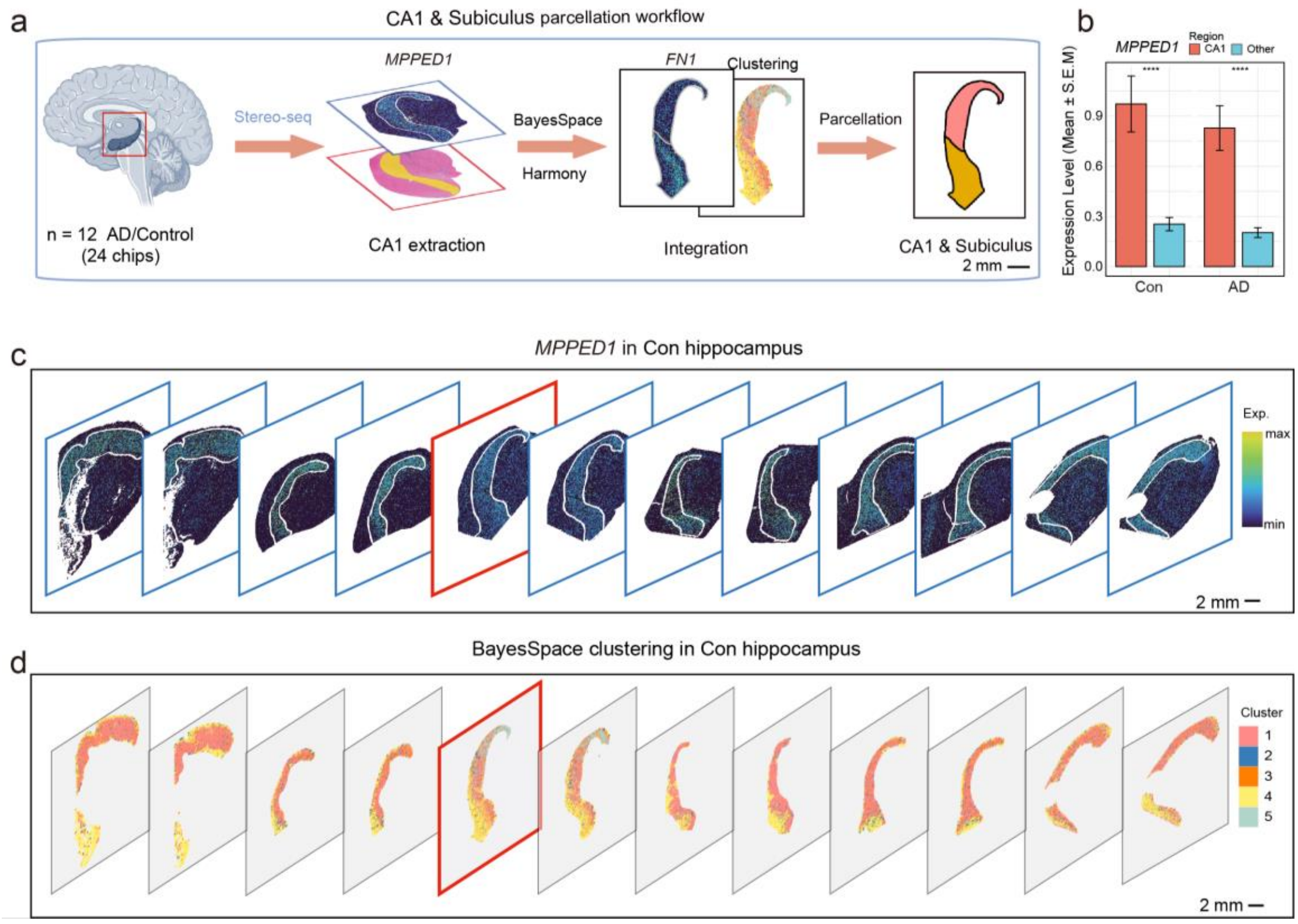

Fig. 1| Region parcellation of human hippocampus by gene expression pattern.

(**a**) The schematic diagram of the study design. A total of 12 samples (24 tissue sections) from AD and Con individuals are performed sequencing. Stereo-seq tissue sections were cut coronally through the hippocampal body. (**b**) Quantitative statistics of the expression level of *MPPED1* gene in CA1 and other brain regions. Data are presented as the mean ± standard error of the mean (S.E.M.). Difference between CA1 and other regions was examined by *t*-test with Holm's correction, **** $p < 0.0001$. The "Other brain regions" refer to the non-CA1 hippocampal subfields captured on the same Stereo-seq chips, specifically DG, CA2–4, FAS (fimbria, alveus, and stratum oriens (SO)) and SLRM (stratum lucidum (SL), stratum radiatum (SR), stratum moleculare (SM)). (**c**) The expression of *MPPED1* gene in hippocampal CA1 brain region of Con individuals. All Con individuals are age- and sex-matched controls without dementia or hippocampal pathology. The chip marked with a red circle contained two brain regions of relatively moderate size, and this chip would serve as the primary subject for display in the main figures. (**d**) The gene expression of internal heterogeneity in the Con CA1 regions based on BayesSpace algorithm. Scale bar: 2 mm.

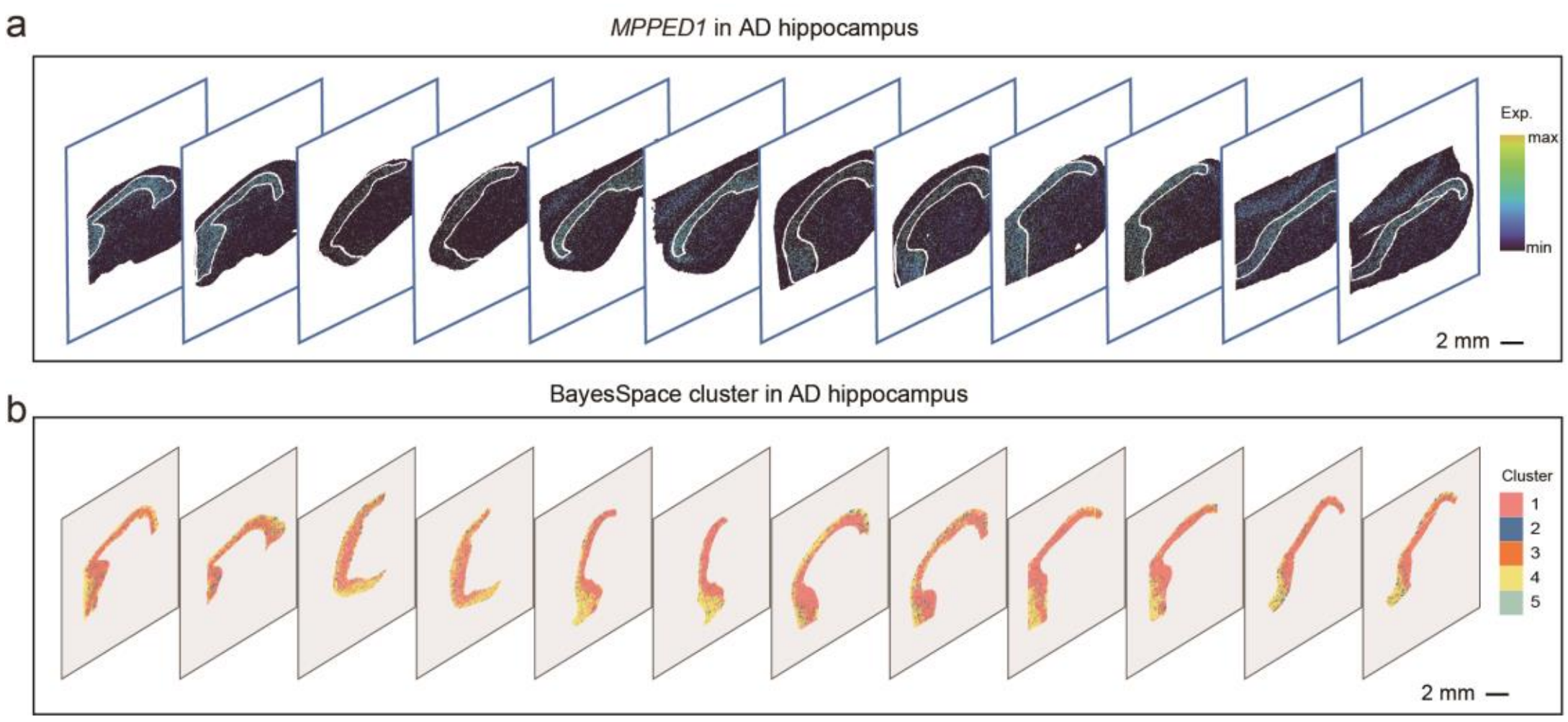

Extended Data Fig. 1| Region parcellation of human hippocampus of AD samples by gene expression pattern.

(**a**) The expression of *MPPED1* gene in hippocampal CA1 brain region in AD patients. (**b**) The gene expression of internal heterogeneity in the AD CA1 regions based on BayesSpace algorithm. Scale bar: 2 mm.

Molecularly Anchoring the Sub-CA1 Boundary

Given the ambiguous definition of the Sub-CA1 boundary in conventional maps[16,17], we hypothesized that one of the two transcriptomic domains corresponded to the Sub. Based on its spatial position, BayesSpace Cluster 4 was the more likely candidate: it localized to the peripheral edge of the putative CA1 territory, consistent with the expected position of Sub. To validate this assignment, we integrated our spatial data with single-nucleus RNA-seq profiles from six human hippocampi[21]. Joint clustering of CA1 and Sub cells identified three distinct Sub neuron subtypes (distal Sub, proximal Sub marked by *COL5A2*, and proximal Sub marked by *SEMA3E*) (**Fig. 2a** and **b**, **Extended data Fig. 2a** and **b**). We next screened established hippocampal cell-type markers to find those enriched in Sub. Strikingly, only *FN1* showed pronounced enrichment in a subset of Sub neurons[21,22] (**Fig. 2c** and **d**, **Extended data Fig. 2c-f**), whereas other candidates either marked different regions (e.g., *ROBO1* labeled CA2–4; **Extended Data Fig. 2c**) or lacked specificity (*COL5A2*, *NTNG1* and *SEMA3E* showed some Sub enrichment but also signal in CA1 or CA2–4; **Extended Data Fig. 2c-e**). Crucially, in our spatial atlas *FN1* expression was almost entirely confined to Cluster 4 and absent from the surrounding CA1 (**Fig. 2e, Supplementary Table 3**). This one-to-one correspondence unequivocally identified Cluster 4 as the Sub, allowing us to use *FN1* as a molecular boundary marker and draw a transcriptionally clear, previously unresolved boundary between CA1 and Sub for the first time (**Fig. 2f, g**; **Extended Data Fig. 2g, h**).

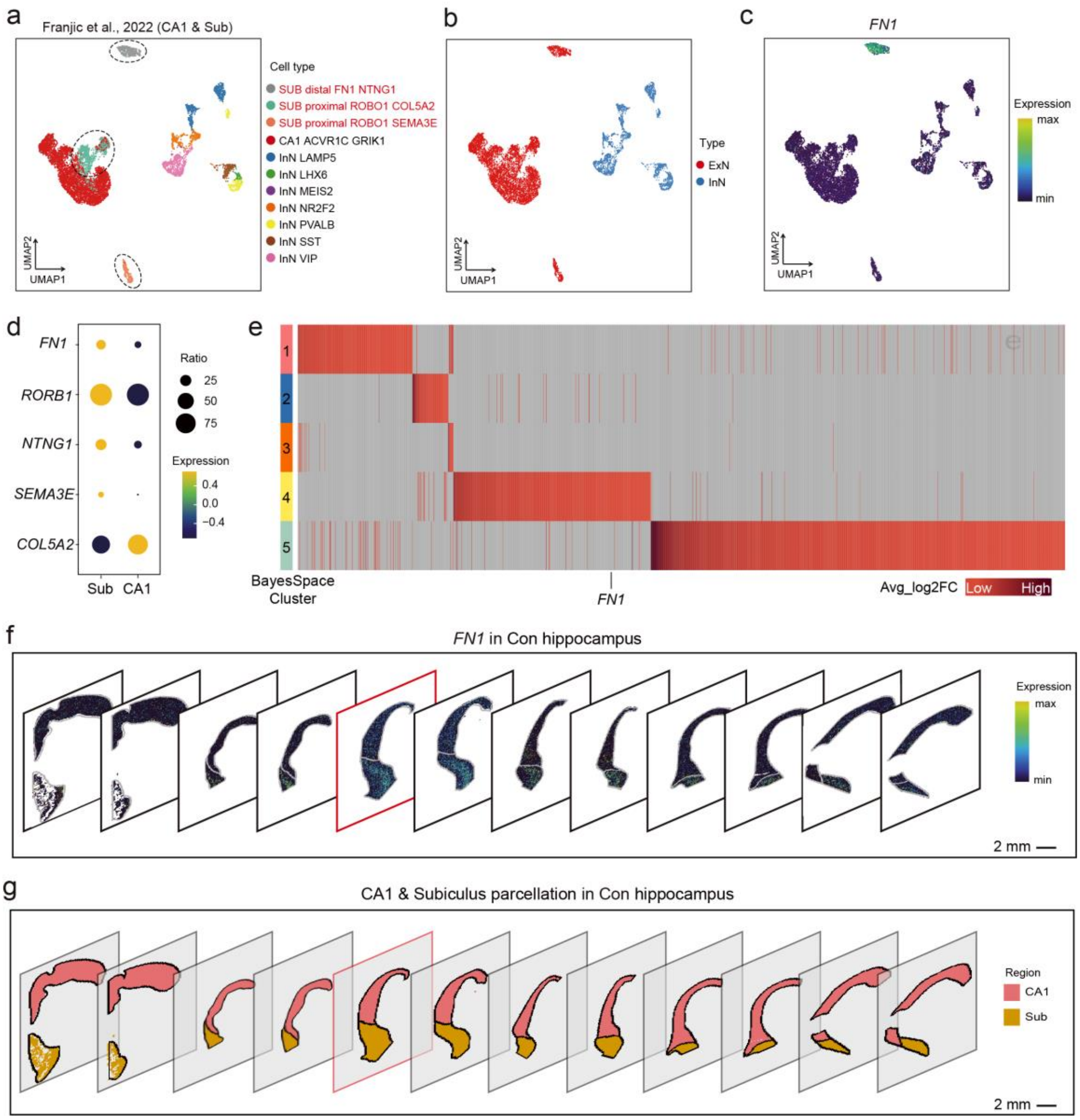

Fig. 2| Identification and screening of marker genes in Sub region using public data. (**a-b**) UMAP visualization of snRNA dataset from the human hippocampus colored by cell types. (**a**) and neuron types (**b**). The UMAP was obtained by extracting cells from the CA1 and Sub brain regions. (**c**) Expression pattern of *FN1* in the UMAP of CA1 and Sub regions. (**d**) Dot plot showing the expression of reported region-specific genes cross brain regions. (**e**) The heatmap showing the differential expression genes of each cluster in BayesSpace. (**f**) Spatial expression of *FN1* in the Con hippocampal regions. (**g**) Parcellation of the Sub and CA1 regions in the Con hippocampal regions. Scale bar: 2 mm.

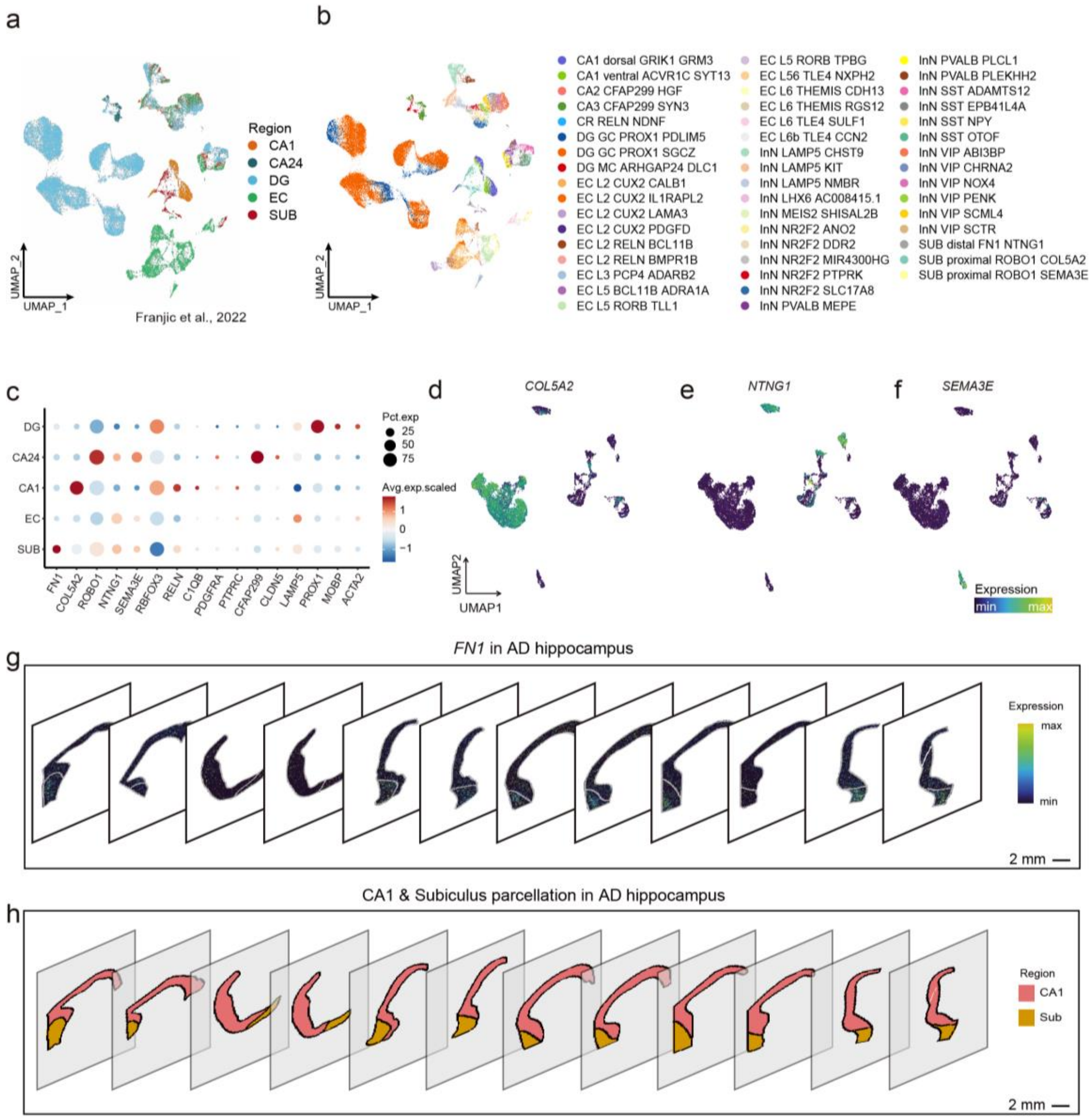

Extended Data Fig. 2| Expression of non-signature genes in public data and confirmation of hippocampal regions.

(**a-b**) UMAP visualization of snRNA dataset from the human hippocampus colored by regions (**a**) and cell types (**b**)[21]. (**c**) Dot plot showing the expression of reported region-specific genes cross brain regions. (**d-f**) Expression of *COL5A2* (**c**), *NTNG1* (**e**) and *SEMA3E* (**f**) genes in the UMAP of CA1 and Sub regions. (**g**) Spatial expression of *FN1* gene in the AD hippocampal regions. (**h**) Parcellation of the Sub and CA1 regions in the AD hippocampal regions.

Characterization and Cross-Platform Benchmarking of the Sub-Specific Molecular Signature

After establishing the spatial demarcation of the Sub, we sought to define the definitive molecular signature that distinguishes this architecture from the adjacent CA1. Differential expression analysis revealed a suite of genes with highly enriched expression in Sub versus CA1 (**Fig. 3a**;

Supplementary Table 4). Among these, we focused on three top markers: ETS variant transcription factor 1 (*ETV1*), proprotein convertase subtilisin/kexin type 1 (*PCSK1*), and calcium/calmodulin-dependent protein kinase II gamma (*CAMK2G*), which were robustly upregulated in the Sub. We verified their regional specificity in the public snRNA-seq dataset[21], confirming that each is predominantly expressed by Sub neurons (**Fig. 3b**). *ETV1*, a transcription factor linked to synaptic plasticity, showed a patchy distribution corresponding to subicular subdomains[23]; *PCSK1*, involved in neuropeptide processing[24], and *CAMK2G*, a kinase regulating calcium signaling[25], were likewise concentrated in Sub. We further quantified *FN1* expression in spatial data, which confirmed *FN1*'s preferential enrichment in Sub compared to CA1 (**Fig. 3c**). Similarly, spatial expression maps and spot-wise quantification for *ETV1*, *PCSK1*, and *CAMK2G* showed graded enrichment across the CA1–Sub transition zone and within Sub, reinforcing their specificity (**Fig. 3d**, **e**; **Extended Data Fig. 3a-e**).

To provide orthogonal validation, we performed RNAscope *in situ* hybridization for *FN1* and *ETV1* using in human hippocampal sections. Both transcripts showed enriched localization in the Sub region, consistent with the spatial patterns identified by Stereo-seq (**Fig. 3f, g**; **Extended Data Fig. 3f, g**). As an additional cross platform assessment, we analyzed a recently published 10X Genomics spatial transcriptomics dataset of human hippocampus[26]. Although the 10X chips had smaller capture areas and did not fully encompass the CA1–Sub continuum, we identified sections containing both regions (**Fig. 3h**). In these sections, *FN1* and *ETV1* showed preferential expression in the Sub, with limited expression in adjacent regions including the CA1 (**Fig. 3i** and **j**; **Extended Data Fig. 3h** and **i**), which providing cross-platform support for the regional enrichment patterns of these representative Sub markers. To further assess whether these regional expression were conserved across species, we examined publicly available *in situ* hybridization data from the Allen Mouse Brain Atlas[27] together with a recently published mouse brain spatial transcriptomic dataset[28]. In the Allen Mouse Brain Atlas, *Fn1* and *Etv1* showed prominent enrichment in the anatomically defined Sub region across multiple mouse brain sections and independent image series (**Extended Data Fig. 4a-b**). Consistently, analysis of the hippocampal regions extracted from 3 mouse spatial transcriptomic sections from the published dataset also revealed higher expression of both genes in the Sub (**Extended Data Fig. 4c-e**). The concordant patterns observed across two independent anatomical and spatial transcriptomic resources provide cross-species support for the preferential expression of *Fn1* and *Etv1* with the Sub region and further support the Sub-enriched expression patterns identified in the human spatial transcriptomic dataset.

Moreover, by digitally partitioning multiple control hippocampi into CA1 vs. Sub regions, we examined expression gradients of key genes across the boundary (**Fig. 3k**, **Extended Data Fig. 3j** and **l**). We found that Sub-specific genes, especially *FN1* and *ETV1*, showed low expression in CA1,

increased across the CA1–Sub transition zone, with levels increasing deeper into Sub. In contrast, *MPPED1*, a CA1-enriched but not CA1-exclusive marker, showed the opposite trend: high in proximal CA1 and tapering off across the boundary into Sub. Interestingly, *NTNG1* and *SEMA3E* also increased gradually along the Sub, suggesting they may serve as markers for proximal-distal subicular sublayers (**Fig. 3l**, **Extended Data Fig. 3k** and **m**). Finally, to explore functional distinctions, we performed disease-relevance enrichment using genes enriched in each operationally defined region (focusing on control samples to avoid AD-related bias). The CA1 signature showed overlap with pathways associated with intellectual disability, whereas the Sub signature was associated with synucleinopathies and dementia (**Fig. 3m**). These descriptive associations indicate that CA1 and the Sub possess regionally distinct molecular profiles. While further study is required to determine if these signatures relate to specific disease vulnerabilities, this underscores our framework's utility in enabling molecularly informed, region-resolved interrogation of brain disease.

Fig. 3

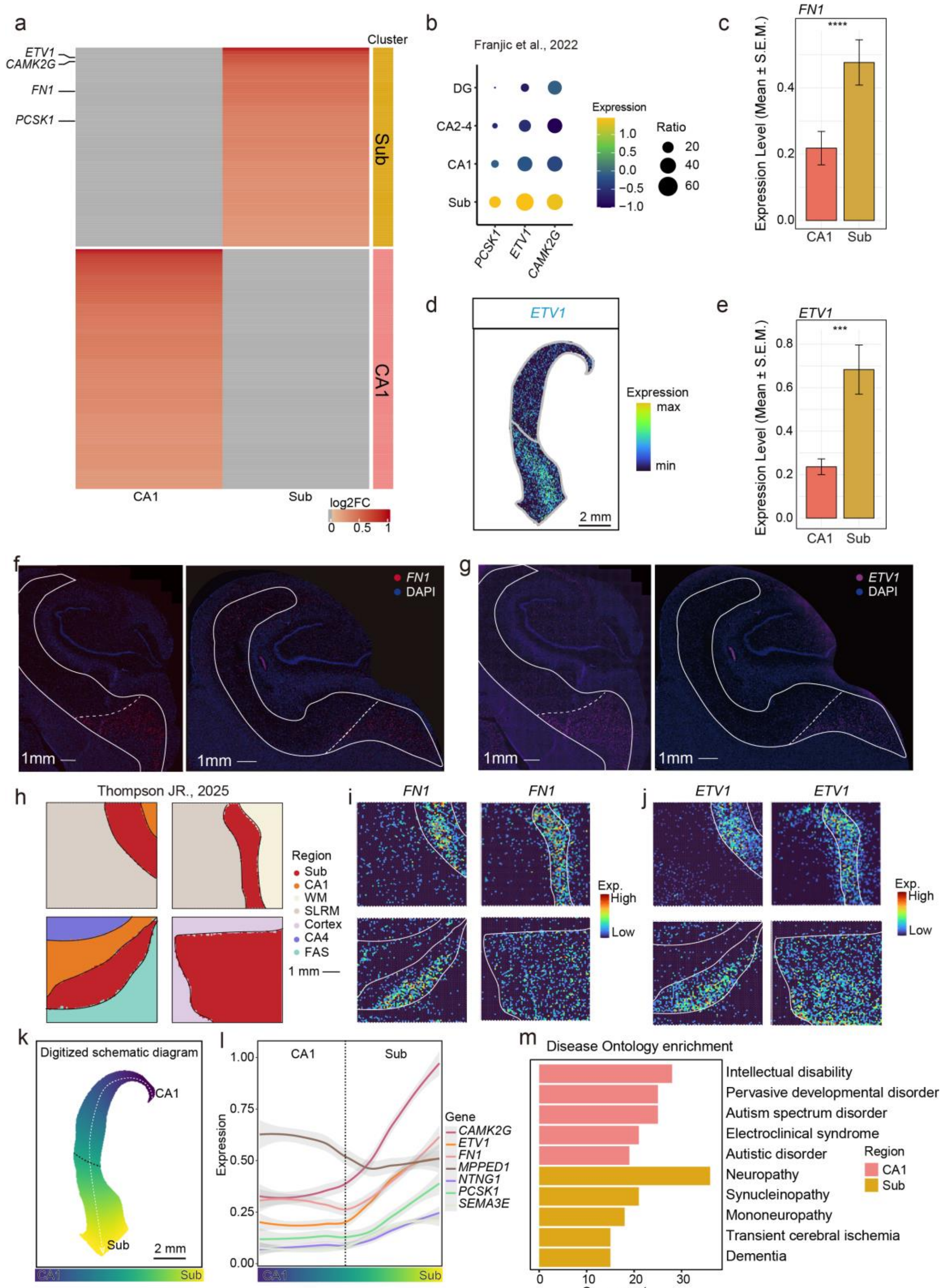

Fig. 3| Analysis of differences between Sub and CA1 regions.

(**a**) Heatmap showing average $\log_2$ (fold change (FC)) of region-specific expressing genes for each area. (**b**) Dot plot showing the marker genes of Sub region in public snRNA dataset. (**c**) Quantitative statistics of *FN1* gene in hippocampal regions. Difference between CA1 and Sub was examined by *t*-test with Holm's correction, *** $p < 0.001$. (**d**) Spatial expression of *ETV1* gene in hippocampal

regions of Con individual. (**e**) Quantitative statistics of *ETV1* gene in hippocampal regions of Con individuals. Difference between CA1 and Sub was examined by *t*-test with Holm's correction, *** $p < 0.001$. (**f**) Verification of *FN1* gene (red) and cells (blue) staining in Con (left) and AD (right) chips respectively by RNAscope. (**g**) Verification of *ETV1* gene (purple) and cells (blue) staining in Con (left) and AD (right) chips respectively by RNAscope. (**h**) Area annotation of public spatial data from Thompson JR et al. 2025[26]. (**i**) The expression of *FN1* in public spatial data. (**j**) The expression of *ETV1* in public spatial data. (**k**) The digitized schematic diagram of CA1 and Sub brain regions. (**l**) The expression of marker genes statistically analyzed after digital stratification. (**m**) Disease Ontology (DO) terms analysis was enriched by Sub/CA1-specific genes.

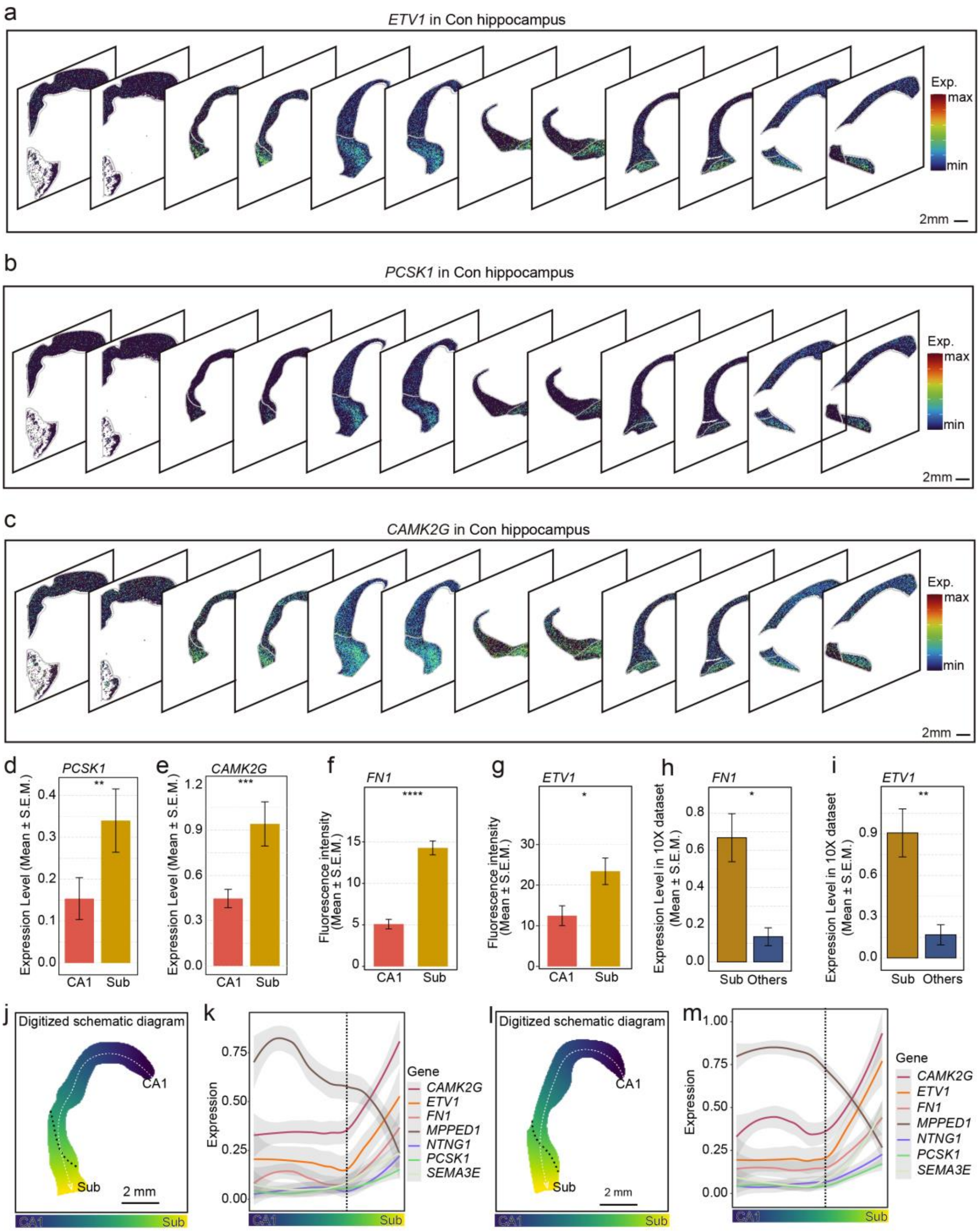

Extended Data Fig. 3| Spatial visualization of *ETV1, PCSK1* and *CAMK2G* genes in all samples

(**a-c**) Spatial expression of *ETV1* (**a**), *PCSK1* (**b**), and *CAMK2G* (**c**) genes in the Con hippocampal regions. Scale bar: 2 mm. (**d-e**) Quantitative statistics of *PCSK1* (**d**) and *CAMK2G* (**e**) in hippocampal regions of Con individuals. Difference between CA1 and Sub was examined by *t*-test with Holm's correction, **** $p < 0.0001$, ** $p < 0.01$. (**f-g**) Background-corrected RNAscope fluorescence intensity was quantified for *FN1* (**f**) and *ETV1* (**g**) in CA1 and Sub regions by randomly sampling 10 subareas per region in each section. (**h-i**) Quantitative statistics of *FN1* (**h**) and *ETV1* (**i**) expression across hippocampal regions in the 10X dataset[26]. (**j** and **l**) The digitized schematic

diagram of CA1 and Sub regions. (**k** and **m**) The expression of specific genes in the CA1 and Sub brain regions after digitalization (**j** and **l**).

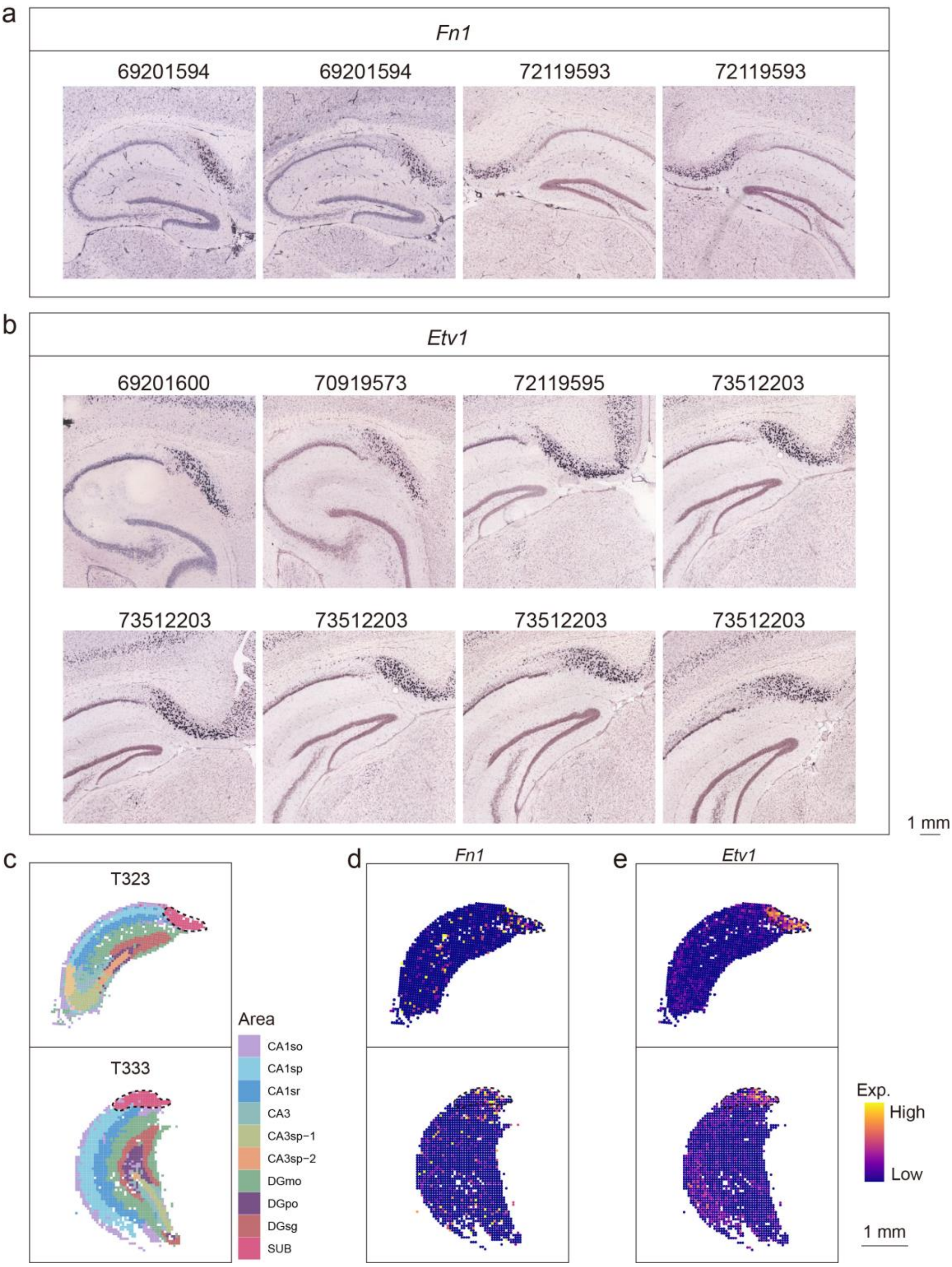

Extended Data Fig. 4| Sub-enriched *Fn1* and *Etv1* expression in the mouse brain

(**a**) *In situ* hybridization images from the Allen Mouse Brain Atlas showing *Fn1* expression in four mouse brain sections, including two sections from image series 69201594 and two from image series 72119593[27]. (**b**) *Etv1* expression in five mouse brain sections from image series 69201600,

70919573, 72119595, and 73512203. (**c**) Anatomical annotations of hippocampal regions in two publicly available mouse brain spatial transcriptomic sections generated by Han et al. (2025)[28]. (**d–e**) Spatial expression patterns of *Fn1* and *Etv1*, respectively, in the corresponding hippocampal sections. Both genes showed consistently higher expression in the Sub across the analyzed sections.

Application Case Study: High-Resolution Parcellation Reveals Region-Specific Transcriptional Remodeling in Alzheimer's Disease

To illustrate the utility of our molecularly anchored framework for resolving region-associated transcriptional changes (**Fig. 3**), we examined AD-associated expression patterns across the defined CA1–Sub interface. Applying the molecular parcellation framework, we identified 467 AD-associated differentially expressed genes (DEGs), comprising 278 upregulated and 189 downregulated genes (**Supplementary Table 5**). These DEGs showed distinct region-dependent transcriptional enrichment patterns, illustrating the spatial heterogeneity of AD-associated transcriptional changes across the CA1-Sub interface.

Among upregulated DEGs, 27 genes were shared between Sub and CA1 and were enriched for processes related to metal ion homeostasis and regulation of apoptosis. Region-biased signals were also evident: CA1-upregulated DEGs (n = 122) were enriched for hypoxia response and gliogenesis-related terms, compatible with glial- and stress-associated transcriptional changes. Sub-upregulated DEGs (n = 129) were enriched for gene sets annotated to ATP metabolic processes and electron transport-coupled pathways[29] (**Fig. 4a** and **b**; **Extended Data Fig. 5a** and **b**). For example, *NDUFA4*, a mitochondria-associated gene involved in respiratory chain function, showed altered expression in Sub in AD, suggesting an AD-associated transcriptional change in oxidative phosphorylation-related pathways in this region[30] (**Fig. 4a** and **b**). Because neuronal loss, cellular stress, and other AD-associated changes may influence region-level and cellular-level expression patterns, transcriptional enrichment in metabolic or stress-response pathways does not necessarily indicate corresponding changes in metabolic activity or cellular function.

Downregulated DEGs in both regions converged on proteostasis-related pathways, while Sub-biased downregulated DEGs showed additional enrichment for synapse organization (**Fig. 4c** and **d**). To assess whether AD preferentially perturbs regional identity programs, we intersected region-specific marker sets with AD-related DEGs. In Sub, 58 Sub markers were downregulated in AD, whereas only three Sub markers (*SNCG*, *TUBB2B*, *RPS27*) were upregulated (**Fig. 4e**; **Supplementary Table 6**); the downregulated Sub markers were enriched for synaptic functional terms (**Fig. 4f**). Notably, *NDUFA4* was not a Sub identity marker, suggesting that AD-related transcriptional remodeling in Sub involves not only attenuation of region-specific programs but also induction of additional stress-associated processes. In CA1, 43 CA1-specific markers were all downregulated in AD (**Extended Data Fig. 5c**; **Supplementary Table 7**), with enrichment pointing

to protein polymerization-related processes (**Extended Data Fig. 5d)**.

To capture coordinated network-level changes, we performed high-dimensional weighted gene co-expression network analysis (hdWGCNA) using AD-associated DEGs and identified 10 co-expression modules[31] (**Fig. 4g**; **Extended Data Fig. 5e**; **Supplementary Table 8**). Module eigengenes showed region-dependent expression patterns and functional annotations (**Fig. 4h**; **Extended Data Fig. 5f-i**). Sub-biased downregulated genes were enriched in modules associated with lipid handling/high-density lipoprotein particle clearance[32], neuronal support function and regulation of synaptic plasticity[18,33] (M2, M5, and M4; **Fig. 4g**; **Supplementary Table 9**). In contrast, Sub-biased upregulated genes were concentrated in an energy metabolism module (M8), enriched for ATP metabolic processes and electron transport-coupled pathways (**Fig. 4g-j**; **Supplementary Table 9**). Network topology further highlighted mitochondria-related hub genes, including *NDUFA4* and *CALM2*[30,34] (**Fig. 4k**).

Collectively, these analyses illustrate the utility of our spatial framework for resolving region-associated transcriptional heterogeneity in AD, providing a region-resolved view of AD-associated molecular heterogeneity across the CA1-Sub interface.

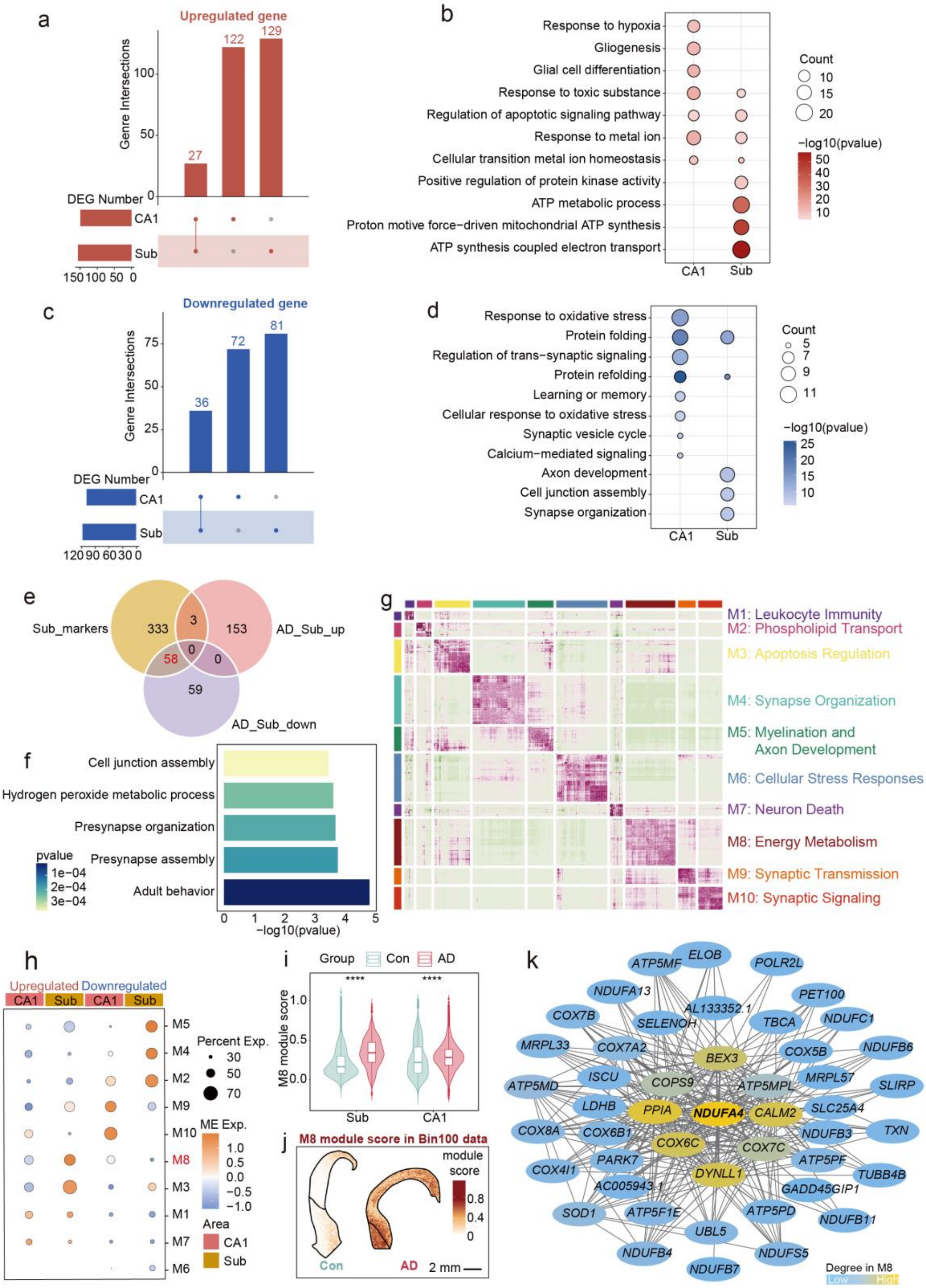

Fig. 4| Differential gene expression and co-expression network analysis in Sub and CA1 regions of AD using bin100 data

(**a** and **c**) The number of upregulated (**a**) and downregulated (**c**) DEGs across the Sub and CA1 regions in AD. (**b** and **d**) Dot plots showing the enriched GO terms for upregulated (**b**) and downregulated (**d**) DEGs in the Sub and CA1 regions, respectively. (**e**) The intersection of Sub-specific genes and the AD DEGs in the Sub region. (**f**) Pathway enrichment analysis Sub-specific

genes downregulated in AD. (**g**) Heatmap showing ten modules of DEGs classified by hdWGCNA, with each correlated to distinct biological processes. (**h**) Dot plot showing the Module eigengenes (ME) for DEGs in Sub and CA1 regions within AD and Con. (**i**) Volin plot illustrating the M8 module gene scores between AD and Con within Sub and CA1 regions in all chips. Difference between AD and Con was examined by *t*-test with Holm's correction, **** $p < 0.0001$. (**j**) Spatial expression of M8 module gene scores in hippocampal regions of AD (right) and Con (left). Scale bar: 2 mm. (**k**) Co-expression network of Intersection of M8 module genes and upregulated genes in AD Sub region visualized using Cytoscape software. Edges represent interactions, and node color indicates connectivity degree.

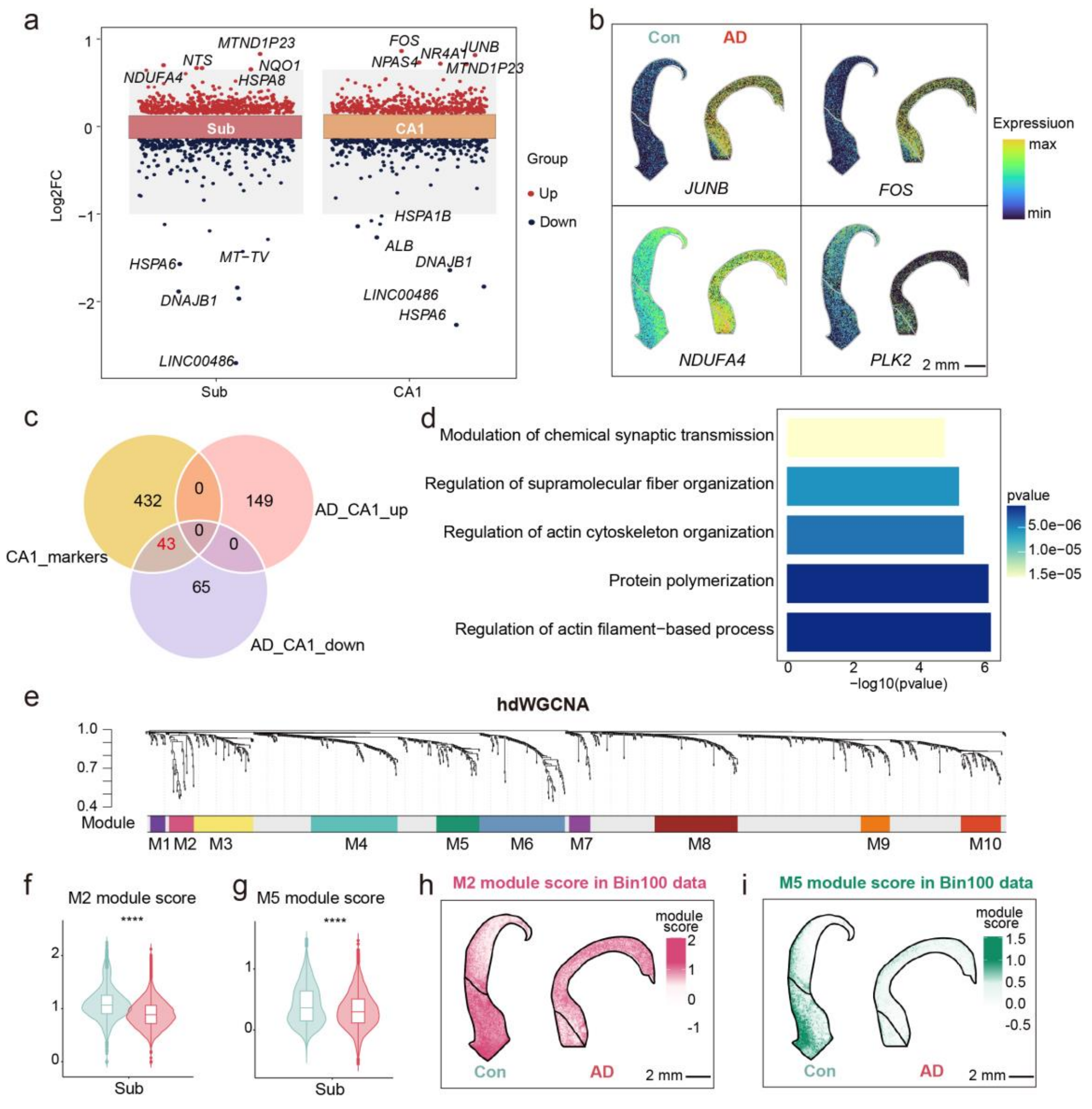

Extended Data Fig. 5| Spatial analysis of AD-linked DEGs and modules in Sub and CA1 regions

(**a**) The volcano plots showing the AD-associated DEGs in the Sub and CA1 regions. (**b**) Spatial expression of *JUNB*, *PLK2*, *NDUFA4*, *CALM2* genes between AD and Con groups. (**c**) The

intersection of Sub-specific genes and the AD DEGs in the Sub region. (**d**) Pathway enrichment analysis CA1-specific genes downregulated in AD. (**e**) Ten co-expression modules of DEGs clustered by hdWGCNA. (**f-g**) Volin plots illustrating the M2 and M5 module gene scores between AD and Con within Sub and CA1 regions. Difference between AD and Con was examined by *t*-test with Holm's correction, **** $p < 0.0001$. (**h-i**) Spatial distribution of M2 and M5 module gene scores of Con (left) and AD (right). Scale bar: 2 mm.

Multi-Scale Mapping Identifies Neuronal Drivers of Sub-specific Bioenergetic Programs

To dissect the cellular contributors to the regional metabolic signature observed in the Sub, we performed brain region-specific alignment by mapping bin100 matrices to cellbin coordinates, yielding a spatially resolved single-cell transcriptomic atlas (**Fig. 5a**; **Extended Data Fig. 6a**). The accuracy of our cell-type annotations, performed via the Spatial-ID method[35], was corroborated by high similarity with public snRNA-seq benchmarks (**Fig. 5b**) and further supported by canonical marker expression and rigorous chip-level quality control across both control and AD cohorts (**Extended Data Fig. 6**).

Quantitative density analysis revealed a significant reduction in neuronal populations, particularly excitatory neurons, alongside an increased microglial density in both the Sub and CA1 regions of AD patients (**Fig. 5c**). In addition, cell-type-specific DEGs analysis showed different expression patterns between Sub and CA1 regions. In the CA1 region, the DEGs across multiple cell types, such as Astro, Micro, OPC, and pericyte, were enriched for pathways related to cellular cation homeostasis and axonal development (**Fig. 5d**, **f**). In contrast, in the Sub region, DEGs were predominantly upregulated in specific neuronal populations, most notably EX_Sub and IN_PVALB neurons, and were enriched for mitochondrial energy metabolism pathways including ATP metabolic processes, aerobic respiration, and oxidative phosphorylation (**Fig. 5d**, **f**).

To connect these cell-type signatures to our region-level co-expression network, we compared cell-type-specific DEGs with the hdWGCNA modules using Jaccard similarity. This analysis highlighted a strong overlap between the mitochondrial/energy module M8 and DEGs from EX_Sub and IN_PVALB neurons (**Fig. 5g**). Consistently, gene set scoring showed significant elevation of M8-associated expression programs in EX_Sub and IN_PVALB neurons in the AD Sub (**Fig. 5h-l**), with increased M8 scores in AD versus controls for both neuronal populations (**Fig. 5i**, **k**) and spatial visualization recapitulating these shifts at the tissue level (**Fig. 5j**, **l**). Finally, analysis of M8 hub genes identified *NDUFA4* as one of the most strongly upregulated electron transport chain-associated genes in AD EX_Sub neurons (**Fig. 5m**, **n**).

Collectively, these results demonstrate that the Sub-specific metabolic signature in AD is not a global tissue response but is preferentially engaged within specialized neuronal populations, a

discovery enabled by our high-resolution parcellation framework.

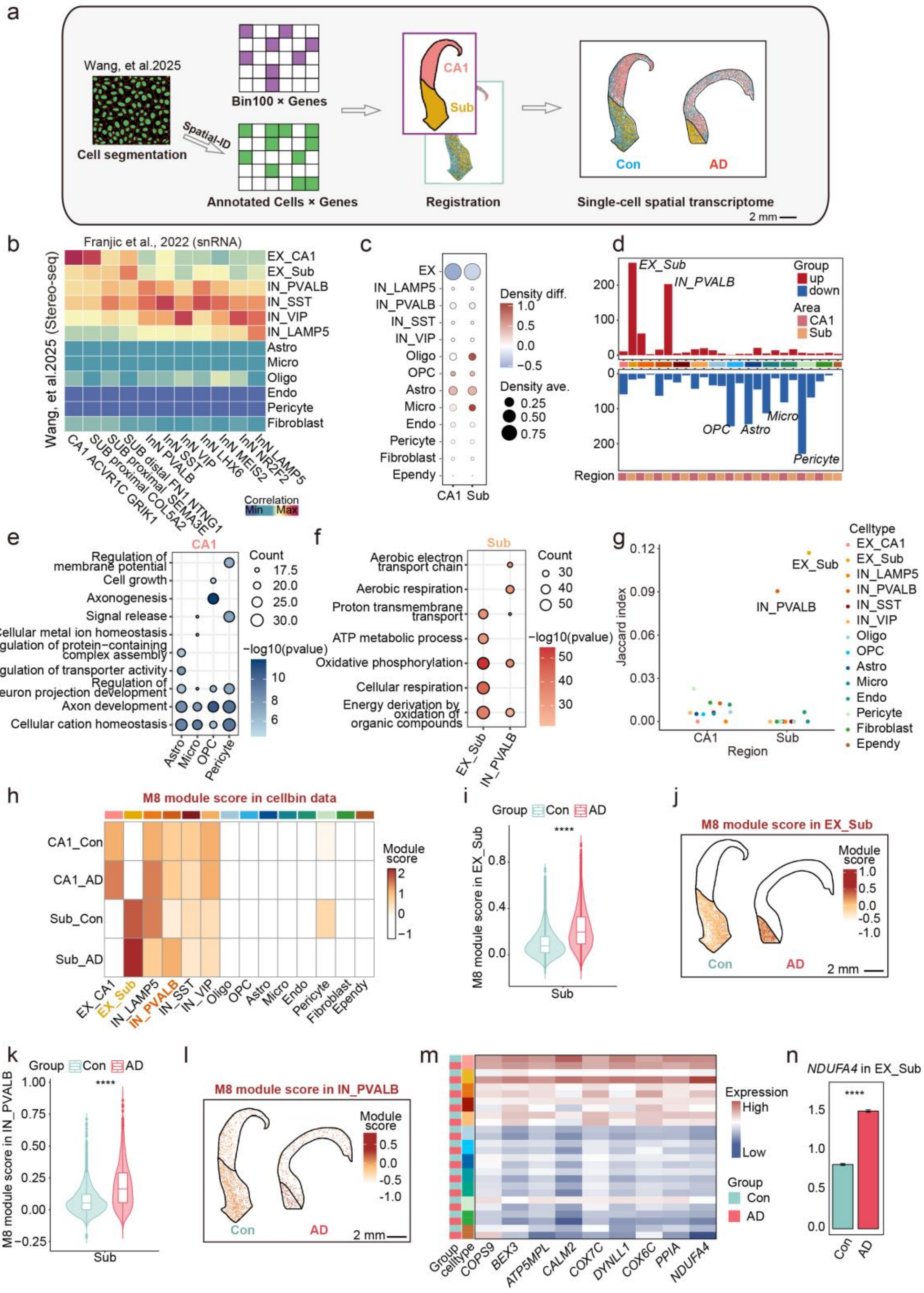

Fig. 5| Single-cell spatial transcriptome analysis revealed cell type-specific gene expression changes in the Sub and CA1 regions of AD

(**a**) The workflow illustrating the integration of bin100 and cellbin matrices to generate spatially resolved single-cell transcriptomic dataset with brain region annotations. (**b**) Pearson correlation of

cell types between snRNA dataset from Franjic et al. (2022)[21] and our stereo-cell data. (**c**) Dot plot depicting cell type densities in the Sub and CA1 regions. The spot size represented the average density of each cell type for AD and Con. The spot color represented an increase (red) or decrease (blue) of cell density in AD. Density diff., Density difference. (**d**) Bar plot quantifying upregulated and downregulated DEGs across cell types in the AD group. (**e**) Dot plot showing the enriched GO terms for downregulated DEGs of Astro, Micro, OPC and Pericyte in the CA1 region. (**f**) Dot plot showing the enriched GO terms for upregulated DEGs of EX_Sub and IN_PVALB neurons in the Sub region. (**g**) Dot plot showing the Jaccard similarity index of M8 module genes and DEGs of different cell types. (**h**) Heatmap of M8 module score across Sub and CA1 regions within the different cell types in AD and Con groups. (**i** and **k**) Violin plots showed the M8 module gene scores between AD and Con for EX_Sub (**i**) and IN_PVALB (**k**) cell types within the Sub region across all chips. Difference between AD and Con was examined by *t*-test with Holm's correction, **** $p < 0.0001$. (**j** and **l**) Spatial expression of M8 module gene scores in EX_Sub (**j**) and IN_PVALB neurons (**l**) of AD (right) and Con (left). Scale bar: 2 mm. (**m**) Heatmap of expression for hub genes (*COPS9*, *BEX3*, *ATP5MPL*, *CALM2*, *COX7C*, *DYNLL1*, *COX6C*, *PPIA*, *NDUFA4* genes) in the Sub region of different cell types. (**n**) Bar plot showing mean expression of *NDUFA4* in EX_Sub neurons of AD and Con groups. Difference between AD and Con was examined by *t*-test with Holm's correction, **** $p < 0.0001$.

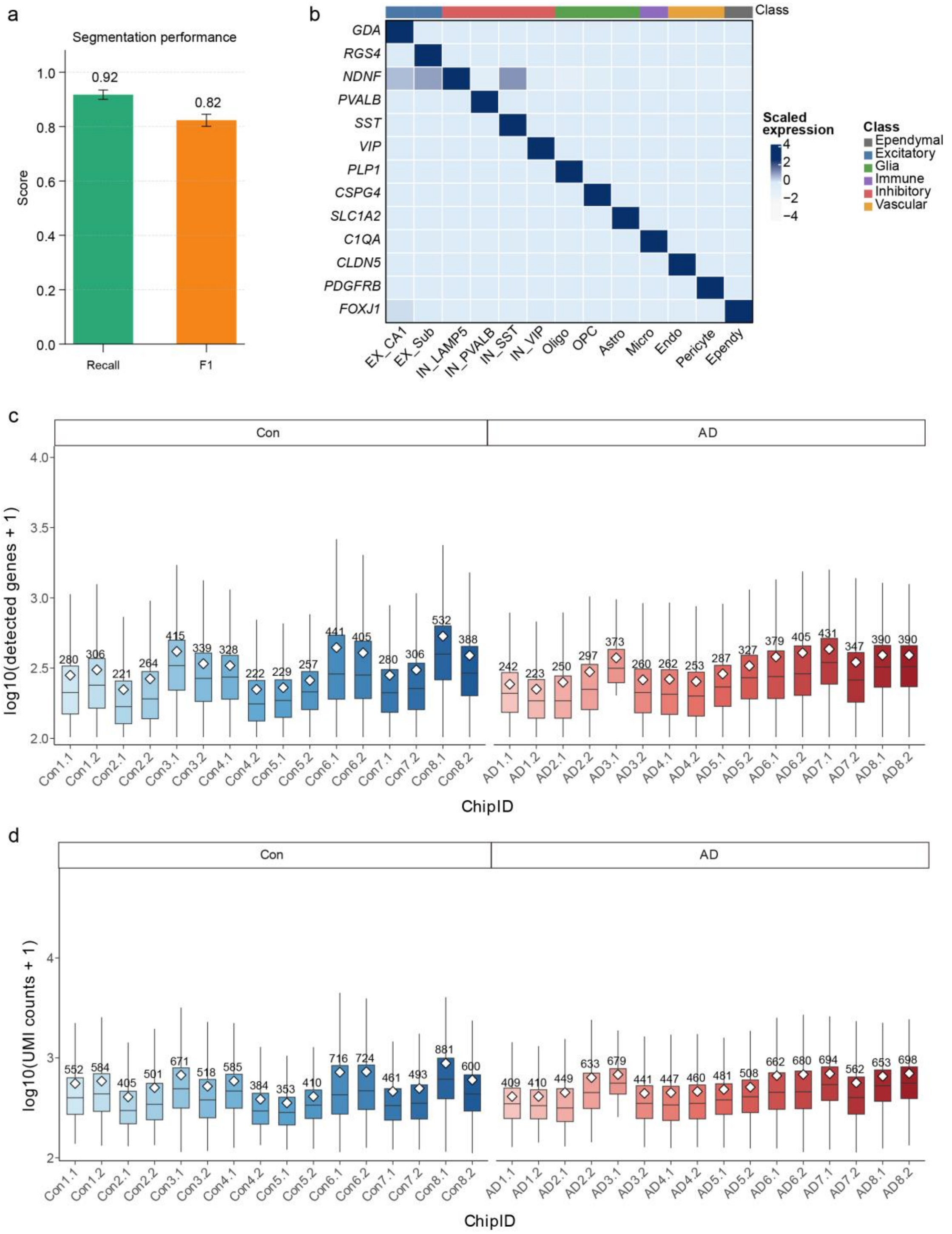

Extended Data Fig. 6| Technical quality control and validation of cell segmentation, cell-type annotation, and spatial transcriptomics.

(a) Benchmark of cell segmentation accuracy via manual inspection across three representative, high-magnification image fields. **(b)** Heatmap displaying the relative expression levels of canonical marker genes across the annotated major cell classes, confirming the specificity and high fidelity of cell-type assignments. **(c–d)** Chip-level quality-control assessments across control and AD post-mortem human hippocampal samples, showing the spatial distribution and density of detected gene

counts **(c)** and UMI counts **(d)** per spatial bin.

Discussion

Accurate identification of CA1–Sub boundary is essential for human brain studies using limited surgical or post-mortem tissue. Our study establishes spatially resolved molecular framework specifically designed to delineate the human CA1–Sub interface. By performing a secondary analysis of a previously published human hippocampal Stereo-seq dataset and integrating the spatial profiles with snRNA-seq reference data, we identified coordinated molecular signatures that consistently distinguished CA1 and Sub across the analyzed donors. *FN1* enrichment in a subset of EX_Sub, together with convergent patterns of *ETV1*, *PCSK1*, *CAMK2G*, and other regional markers, supported reproducible assignment of CA1 and Sub identities. The regional association of *FN1* and *ETV1* with the Sub was further supported by three independent brain resources[26-28]. To our knowledge, this represents the first molecularly anchored, cross-platform validated coordinate system for the human CA1–Sub interface, reframing a problem historically treated as a matter of anatomical judgment into one that can be resolved computationally and reproducibly from gene expression data.

It is important to clarify the nature of the CA1–Sub boundary identified in this study. Traditionally, the CA1–Sub interface has been delineated primarily according to cytoarchitectural features, including changes in pyramidal-cell density, layer thickness, cellular morphology, and laminar organization[36,37]. However, the precise placement of this boundary remains challenging because these features can vary gradually across the interface and may differ across individuals and along the rostrocaudal axis[38]. More recent single-cell and spatial transcriptomic studies have further shown that the Sub is molecularly heterogeneous, comprising spatially organized neuronal populations distributed along its proximal–distal, superficial–deep, and longitudinal axes rather than forming a molecularly uniform compartment[21,39,40].

Our findings reconcile these apparently discrete and continuous features of subicular organization. At the multigene level, Bayesian spatial inference identified a reproducible and visually distinct interface between these domains across tissue sections[20]. Thus, although the CA1–Sub boundary has traditionally been difficult to define consistently using cytoarchitectural criteria alone, the combined expression of multiple regional markers provided a clearer and more reproducible distinction[38]. In contrast, examination of individual genes revealed a more gradual biological transition. CA1-enriched *MPPED1* decreased toward the Sub, whereas *FN1*, *ETV1*, *PCSK1*, and *CAMK2G* increased across the CA1–Sub interface. *NTNG1* and *SEMA3E* showed additional progressive variation along the proximal–distal axis of the Sub. Even *FN1* and *ETV1*, despite their strong regional enrichment, did not exhibit an abrupt step-function change at a single anatomical line. We therefore interpret the CA1–Sub boundary not as an absolute discontinuity in the

expression of individual genes, but as a probabilistically defined transition between two distinguishable multivariate molecular states. This interpretation is consistent with previous transcriptomic studies demonstrating both discrete, spatially segregated EX_Sub subclasses and continuous molecular organization within the subicular complex. The principal advance of our framework is that it moves beyond subjective cytoarchitectural assessment or reliance on a single marker by integrating spatial information, multigene expression patterns, unsupervised clustering, and Bayesian boundary inference to produce a reproducible, biologically informed operational parcellation in the human hippocampus. In this framework, the boundary is evident at the level of the integrated multigene molecular pattern, even though individual marker genes may vary gradually across the interface. This approach therefore enables more consistent CA1 and Sub annotation and region-resolved comparison across donors and datasets, while preserving the underlying biological continuum of the CA1–Sub transition.

This advance has immediate practical consequences for the field. A long-standing limitation in human hippocampal subfield research is the "boundary-spanning" dilemma: because the CA1-Sub transition cannot be reliably assigned using conventional landmarks, tissue spanning this interface is frequently excluded from analysis to avoid misclassification, resulting in substantial underuse of scarce post-mortem specimens[41,42]. Our molecular reference resolves this dilemma by enabling principled, gene expression-based assignment of transition-zone tissue, thereby increasing the analyzable yield from each donor and improving cross-laboratory reproducibility[43]. More broadly, this framework offers a template that could, in principle, be extended to other architecturally ambiguous, continuum-like boundaries within the hippocampal formation and adjacent cortical regions, where gradual transitions, rather than sharp anatomical borders, are the rule rather than the exception.

Beyond this practical gain in tissue utilization, we believe the more fundamental impact of this work lies in a methodological shift it enables for the field. Region definition in human hippocampal subfield research has traditionally depended on expert-driven, qualitative interpretation of cytoarchitecture, a process that is inherently difficult to standardize across laboratories, donors, and even between sections from the same specimen. By demonstrating that a small set of molecular markers can reproducibly recover a biologically meaningful transition zone across independent platforms and cohorts, our framework moves CA1-Sub regional assignment from a matter of anatomical expertise toward a quantitative, data-driven, and externally verifiable procedure. This shift has direct downstream consequences: it allows region-resolved comparisons (such as the AD analyses presented here) to be benchmarked against an explicit molecular criterion rather than an implicit and potentially inconsistent anatomical judgment, thereby improving the comparability of

findings not only within a single study but, in principle, across future studies that adopt the same molecular reference.

To contextualize this contribution within the existing literature, prior attempts to characterize the CA1–Sub continuum in human tissue have largely relied on either single-marker immunohistochemistry or bulk-tissue dissection guided by visual anatomical estimation[41,44], both of which carry inherent risk of regional cross-contamination and limited reproducibility across laboratories and donors. Rodent studies have provided higher-resolution insights into Sub molecular identity, but cross-species differences in hippocampal organization limit the direct translation of these findings to human tissue. We therefore used the mouse data as independent anatomical support rather than as the basis for defining the human CA1–Sub boundary. Specifically, both *in situ* hybridization data from the Allen Mouse Brain Atlas and three independent mouse brain spatial transcriptomic sections from our previous study [28]showed preferential expression of *Fn1* and *Etv1* in the Sub, indicating that the regional enrichment of these markers is detectable across species and technical platforms. Nevertheless, this cross-species concordance does not imply that the precise cellular distribution or geometry of the CA1–Sub transition is identical between mouse and human. Our broader validation strategy, integrating human Stereo-seq, snRNA-seq, RNAscope, an independent 10X Genomics dataset, and complementary mouse *in situ* hybridization and spatial transcriptomic resources, establishes convergent evidence across independent cohorts, species, and experimental platforms. Together, these results increase confidence that the *FN1*- and *ETV1*-associated Sub-enriched patterns observed in the human data reflect reproducible anatomical organization rather than a platform-specific or donor-specific artifact.

Building on this molecular reference, we performed region-resolved comparisons in AD as a proof-of-concept application. These analyses revealed divergent transcriptional programs across the CA1-Sub interface, including a more prominent induction of mitochondrial energy metabolism-related signatures within the Sub and stress/glial-associated responses in CA1, with the Sub-specific bioenergetic signature further localized to specific neuronal populations (notably EX_Sub and IN_PVALB neurons) at single-cell resolution. We emphasize that these findings are intended to illustrate the discriminating power of our parcellation framework rather than to establish definitive disease mechanisms. Genes such as *NDUFA4*, which emerged as a hub gene within the Sub-enriched energy metabolism module, represent prioritized candidates for future mechanistic investigation into regional vulnerability in AD, rather than validated causal drivers[45]. Further functional studies will be needed to determine whether this bioenergetic remodeling reflects a compensatory response to metabolic stress or a maladaptive process contributing to regional vulnerability.

Several limitations inherent to human brain banking should be acknowledged[46]. First, the use of post-mortem tissue precludes longitudinal assessment of disease progression and may limit sensitivity to molecular changes that precede overt pathology[47]. Among donors with available metadata, we did not detect statistically significant differences between the AD and Con groups in post-mortem interval or RNA integrity number. However, given the cohort size, these results should not be interpreted as formal evidence of equivalence. Moreover, cause of death and agonal-state information was incomplete, and residual confounding related to terminal events and other donor level clinical or technical factors cannot be excluded. Such factors may influence post-mortem gene expression, particularly that of immediate-early and stress responsive genes, such as *FOS*, *JUNB*, *NR4A*, and *NPAS4*. Consequently, the observed metabolic and stress-response signatures cannot be fully disentangled from the potential effects of neuronal loss, terminal stress, and other donor-level clinical or technical factors and should therefore be interpreted as exploratory transcriptional associations rather than direct evidence of altered metabolic function. Second, although our cohort of 12 donors, representing all specimens available in the brain bank with matched key covariates, was carefully balanced for age and sex to maximize inter-group comparability, this sample size does not fully capture the broad spectrum of inter-individual variability across AD stages. Specifically, the AD cohort spanned Braak stages III to V, with most cases classified as Braak stage IV, while control cases showed absent to low Braak pathology (not detected to Braak stage II); this pathological heterogeneity within a relatively small AD cohort may contribute to inter-donor variability in region-resolved transcriptional signatures and should be considered when interpreting AD-associated findings. Other donor-level variables, including APOE genotype, may further influence individual variability but could not be fully assessed given the limited sample size. These constraints reflect the inherent unpredictability of donor availability and the strict tissue-integrity requirements of spatial transcriptomics[48], rather than a limitation of study design. Third, the co-expression modules and hub genes identified here, including the M8 mitochondrial module, provide valuable correlative insight but require independent experimental validation to establish causal relationships to AD pathology. Accordingly, the RNAscope and 10X analyses should be interpreted as orthogonal and supportive validation of selected representative markers, rather than as exhaustive validation of all candidate genes identified in this study. Future studies prospectively collecting larger, stage- and genotype-stratified cohorts will be important to validate the generalizability of this molecular reference and to clarify how AD stage, APOE genotype, and other donor-level variables influence CA1-Sub boundary delineation and region-resolved disease signatures.

In conclusion, this study provides a high-resolution molecular atlas and a reproducible, transferable parcellation pipeline for the operationally defined human CA1-Sub interface, addressing a transition that has eluded consistent definition since the earliest anatomical descriptions of the hippocampus.

By identifying definitive boundary markers and demonstrating their utility in unmasking region-specific bioenergetic remodeling in AD, our findings provide a foundational resource for reproducible hippocampal subfield research and for region-resolved investigations into neurodegenerative disease.

Methods

Dataset acquisition and preprocessing

The sequencing datasets analyzed in this study were obtained entirely from previously published studies. The high-resolution spatial transcriptomic datasets were obtained from the study by Wang et al. (2025)[18] and are deposited in the China National GeneBank DataBase (CNSA) under accession number CNP0005077. The present study represented a secondary, boundary-focused reanalysis of these Stereo-seq data, specifically addressing the molecular organization and delineation of the CA1-Sub interface. For this purpose, a broad region of interest encompassing CA1 and the Sub was extracted from the complete hippocampal sections with reference to the spatial expression pattern of *MPPED1*[18]. This region of interest was subsequently used for downstream analyses of spatial gene-expression changes and the CA1-Sub boundary. The complementary snRNA-seq datasets were obtained from Franjic et al. (2022)[21] and is publicly available through the Gene Expression Omnibus (GEO) under accession number GSE186538. This dataset contains transcriptomic profiles from multiple micro-dissected hippocampal and parahippocampal regions, including DG, CA2–4, CA1, Sub, and entorhinal cortex (EC), collected from clinically unremarkable adult human donors. This dataset was used to characterize the spatial specific marker genes related to CA1-Sub interface identification. An independent 10X spatial transcriptomic dataset generated by Thompson JR et al. (2025)[26], available under GEO accession number GSE264624, was used for external cross-platform validation. This previously published dataset was used to determine whether the candidate Sub-enriched markers identified through the Stereo-seq and snRNA-seq analyses showed reproducible spatial enrichment in the Sub on an independent spatial transcriptomic platform. For additional cross-species anatomical validation, publicly available *in situ* hybridization images were obtained from the Allen Mouse Brain Atlas and used to examine the hippocampal expression patterns of *Fn1* and *Etv1* in the adult mouse brain[27]. The analyzed images included independent experiment series for *Fn1* and *Etv1*, as specified in the corresponding figure legend. In addition, two publicly available mouse brain spatial transcriptomic sections generated by Han et al. (2025) [28] were analyzed to assess the spatial expression patterns of *Fn1* and *Etv1* across anatomically annotated hippocampal regions[28]. These mouse datasets were used as complementary cross-species evidence to evaluate whether the Sub-enriched expression patterns observed in the human datasets were conserved in the mouse hippocampal formation.

Spatial Transcriptomic Sample Selection

To ensure high data fidelity, the specimens utilized in this study were sourced from an initial pool of 16 hippocampal samples with RNA integrity number (RIN) > 6. From this initial pool, 12 specimens (6 AD and 6 Control) were prioritized for subsequent spatial transcriptomic analysis. Sample selection was based on histological evaluation of tissue architecture and anatomical

coverage. Specifically, samples were included if they showed well-preserved tissue morphology, a continuous CA1 region, and the presence of an identifiable and analyzable Sub region, which was required for CA1–Sub-specific downstream analyses. Specimens exhibiting significant tissue fragmentation, freezing artifacts, poor tissue integrity, or incomplete anatomical coverage of the CA1–Sub region were excluded from CA1-Sub-specific analyses. In particular, tissue sections that did not contain an identifiable and analyzable Sub region were not used for downstream analyses requiring CA1–Sub comparison or parcellation. This targeted selection ensured that the analyzed samples provided sufficient structural integrity and spatial coverage for molecularly informed CA1–Sub parcellation. In addition, chips showing marked global transcriptomic heterogeneity relative to the rest of the cohort, as assessed by PCA, were excluded to minimize chip-level outlier effects. This targeted selection ensured that the analyzed samples provided sufficient structural integrity, spatial coverage, and transcriptomic consistency for molecularly informed CA1–Sub parcellation. The inclusion status of each chip is summarized in **Supplementary Table 1**, where we indicate whether each chip was included in the CA1–Sub analysis and, for excluded chips, provide the corresponding exclusion reason, including absence of an analyzable Sub region or excessive transcriptomic heterogeneity. Detailed donor characteristics, including age, sex, post-mortem interval (PMI), APOE genotype, clinical diagnosis, and Braak stage, are summarized in **Supplementary Table 1**. Control donors exhibited absent to low levels of AD neuropathology, ranging from no detectable pathology to Braak stage II, whereas AD donors ranged from Braak stages III to V, with the majority classified as Braak stage IV.

Stereo-seq raw data processing

The fastq files generated from Stereo-seq were analyzed in accordance with the established pipeline, which can be found at GitHub [49]. To begin with, the initial reads containing coordinate identity (CID) sequences were aligned to the predefined coordinates of the Stereo-seq section, using the results obtained from the first sequencing round as a reference. During this alignment, a maximum of one base mismatch was permitted to accommodate potential sequencing errors. Subsequently, reads with molecular identifiers (MIDs) that had more than two bases with a quality score lower than 10 were filtered out to maintain data quality. For each read that passed these criteria, the corresponding CID and MID were integrated into the read header. The remaining reads were then aligned to the reference genome using STAR, and only those with a mapping quality score exceeding 10 were retained for gene annotation, thereby ensuring the precision of gene expression analysis. Unique molecular identifiers (UMIs) with identical CIDs were merged into a single UMI, allowing for a single mismatch to correct any sequencing or PCR errors. Ultimately, the expression profile matrix containing CIDs was generated.

Image-based single-cell segmentation of Stereo-seq data with StereoCell

AI-assisted cell segmentation was performed on the Stereo-seq data following a previously reported procedure[50]. Briefly, total UMI counts were computed for each DNA nanoball (DNB) coordinate and assembled into a spatial density matrix. The matrix was converted into a grayscale image in which each pixel represented a DNB position and pixel intensity reflected the summed UMI signal. The nucleic-acid staining image from the same tissue section was manually registered to the Stereo-chip coordinate space. Cell segmentation was then carried out using an ESPANet-based deep learning model. Predicted masks were refined using watershed-based post-processing to generate binary cell segmentation results. For each segmented cell, UMIs from all DNBs within the cell mask were aggregated at the gene level, yielding a cell-by-gene count matrix for downstream analyses.

Stereo-Seq data analysis and visualization

To comprehensively characterize the spatial transcriptomic landscape within the region, the bin size of 100 (100 × 100, 50 × 50 μm) was utilized as a fundamental unit (spot) for subsequent analysis. After generating the Bin100 data, we performed quality control on it, and finally retained Bin units with a mitochondrial gene ratio of less than 20% and the number of detected genes greater than 500. Ultimately, the average data quality of bin100 was 2,470 genes/bin100 and 5,573 UMIs/bin100. The expression data matrix derived from these bin100 units, inclusive of their spatial coordinates, was subsequently employed to construct a Seurat object utilizing the Seurat package in R (v4.2.2).

Spatial transcriptome hippocampal region clustering

Transcriptome data from the defined partitions (bin100) on each section were utilized to generate normalized SingleCellExperiment objects, which served as input for subsequent BayesSpace clustering[20]. The BayesSpace package (v1.8.0) was used to cluster the data, thereby annotating the brain regions. Brain regions were annotated and delineated using marker genes identified from clustering subgroups through the *FindAllMarkers* function. The 2D outlines of these brain regions were processed and recognized using the imager R package (v0.42.16). This approach enabled the precise description of hippocampal regions, facilitating the annotation of hippocampal regions and the exploration of heterogeneity within the CA1 region.

Quality control and cell clustering of snRNA-seq dataset

During data preprocessing, two filtering criteria were applied to remove low-quality cells: cells with a mitochondrial gene ratio exceeding 10%, and cells with fewer than 500 detected genes. The data was normalized via the *NormalizeData* function. The top 3000 highly variable genes were selected using the *FindVariableFeatures* function. Then, the expression data was scaled to eliminate technical variation using *ScaleData* function. For different samples, we used the Harmony package

(v0.1) to integrate the samples, thereby eliminating the impact of inter-sample differences. For downstream clustering and visualization, dimension reduction based on Principal Component Analysis (PCA) was first performed, and the first 30 Principal Components (PCs) were extracted for subsequent Louvain clustering to define cell clusters. Finally, the clustering results were visualized in a two-dimensional space using Uniform Manifold Approximation and Projection (UMAP), and the cell clusters were annotated. Cell type annotation was conducted based on the information labeled in the original data. After completing the integration and clustering of single-cell data using Seurat functions, we further extracted cells from the Sub and CA1 regions for secondary clustering based on the cell annotation information within the data, aiming to exclude potential interference from CA2–4 and DG cells. Finally, by comprehensively analyzing the "clustering results of cells from all neuronal brain regions" and the "independent clustering results of Sub and CA1 cells," we collectively verified the expression pattern of Sub region-specific genes.

Cell type annotation of segmented cells by Spatial-ID

Cell type annotation on segmented Stereo-seq data was performed using Spatial-ID[35], implemented with PyTorch (v1.13.1), PyTorch Geometric (v2.1.0), and Python (v3.9.10). The workflow consisted of two stages: First, a four-layer deep neural network was trained on snRNA-seq data using shared genes as anchors to generate initial cell type probabilities for Stereo-seq cells. Second, a graph convolutional network integrated spatial context encoded in an adjacency matrix (normalized Euclidean distances) with gene expression data. This GCN architecture comprised a deep autoencoder for gene expression, a variational graph autoencoder for spatial neighborhoods, and a classifier module. The autoencoders extracted latent representations via self-supervised reconstruction losses, while the classifier mapped these features to cell types through supervised learning aligned with initial probability distributions. Joint optimization of all components over 300 epochs refined cell type assignments. Final annotations were determined by maximum GCN-derived probabilities, effectively reducing batch effects across Stereo-seq sections.

Quality control of single-cell Stereo-seq data

The MID count matrix from Stereo-seq underwent quality control filtering with the Seurat R package across all 24 chips. Cells expressing fewer than 100 genes or containing mitochondrial transcripts above 20% were excluded to ensure data quality. This filtering strategy was uniformly applied to maintain consistency across sections. The average quality of cellbin data was 430 genes/cell and 767 UMIs/cell.

Correlation analysis of cell types between Stereo-seq and snRNA-seq data

Normalization of Stereo-seq and snRNA-seq data was conducted using Seurat's RNA assay

framework. Top variable genes common to both datasets were identified and subjected to Pearson correlation analysis to establish cell type correspondence between the two platforms.

Aligning Bin100 & Cellbin for Brain Region Annotation

For brain region annotation of cellbin data, we first propagated the brain region annotation information from bin100 data to bin1 units. Subsequently, based on the positional correspondence between each bin1 and cellbin, we assigned the brain region annotations from bin1 to the corresponding cellbin, thereby achieving brain region annotation of the cellbin data.

Differential expression analysis

For Sub-specific genes and AD-specific genes in different brain regions, FindMarkers function of Seurat was used to analyze the DEGs between the AD and Con samples ($log_2FC > 0.25$, $p < 0.05$, min.pct > 0.1). For subsequent visualization and statistical comparison of selected differentially expressed genes, expression values were first summarized within each donor for the corresponding anatomical region. Each donor was treated as an independent biological replicate. Group-level values are presented as the mean ± standard error of the mean (S.E.M.), calculated across donors, rather than across individual cells or spatial spots. Statistical comparisons between groups were likewise performed using donor-level summarized expression values.

Functional enrichment analysis

Disease Ontology (DO) analysis was performed using the EnrichDO function in the clusterProfiler R software package[51] with the following parameters: *p*.adjust = 0.05.

Gene ontology (GO) analysis was performed using the EnrichGO function in the clusterProfiler R software package with the following parameters: *p*.adjust = 0.05.

Identification of Clinically Significant Modules

Gene co-expression network analysis was performed using the hdWGCNA R package (v0.2.17)[31]. A co-expression network was constructed from the spatial transcriptomic dataset, and the resulting adjacency matrix was transformed into a topological overlap matrix (TOM). Genes were hierarchically clustered according to TOM-based dissimilarity and assigned to co-expression modules. Module eigengenes were then calculated and correlated with AD status and Sub identity to identify disease- and region-associated modules. Within modules showing significant associations, hub genes were defined according to their intramodular connectivity.

Protein-Protein Interaction (PPI) Network Construction and Hub Gene Identification

The PPI network was visualized by Cytoscape software (v3.10.0)[52]. The cytoHubba plug-in in

Cytoscape was employed to identify hub genes in PPI network based on degree method[53]. The components in the central node could be considered as the core proteins and significant hub genes with essential physiological functions.

RNAscope validation of marker genes

RNAscope™ Multiplex Fluorescent v2 assay for fixed frozen samples was performed using the RNAscope™ Multiplex Fluorescent Kit (Cat. No. 323100) according to the manufacturer's instructions (sbio Technology, Inc.). Briefly, to achieve close spatial and anatomical correspondence with the spatial transcriptomic data, RNAscope validation experiments using 2 × 2 cm capture areas were performed on cryopreserved, nearby region-matched tissue sections collected from the same hippocampal tissue blocks used for Stereo-seq profiling[18]. These sections were anatomically matched to the Stereo-seq sections but were not necessarily serially adjacent sections. Each section was used for orthogonal validation of candidate CA1-Sub marker genes. In total, two sections were used for this validation assay, one from the cognitively control group and one from a donor with AD. Tissues were fixed in fresh 4 % PFA (1 × PBS) at 4 °C for 24 h, embedded in OCT post-sucrose gradient treatment, frozen with dry ice/liquid nitrogen, stored at -80°C, sectioned into 7-15 μm slices on SuperFrost® Plus slides (Epredia, 4951PLUS-001E), dried at -20°C for 60-120min, washed with 1 × PBS, baked at 60°C for 30min, and post-fixed in pre-cooled 10 % NBF/4 % PFA at 4 °C for 15 min before dehydration with gradient ethanol and air-drying. The following fluorescence channels were used for RNAscope probes: *FN1* (C1) and *ETV1* (C2). Sections were counterstained with DAPI (RT, 1-2 min), mounted with ProLong Gold (Thermo, P36930).

Quantification of RNAscope Fluorescence Intensity in CA1 and Sub Regions

RNAscope fluorescence quantification was performed on annotated fluorescence images for *FN1* and *ETV1*. The CA1 and Sub regions were defined according to the white annotated boundaries in each image. For each chip, 10 square subareas were randomly sampled within the CA1 region and 10 square subareas were randomly sampled within the Sub region. Subareas were retained only when they fell within the corresponding annotated region. Target fluorescence was quantified from the magenta signal channel. Background intensity was estimated from dark background pixels in the same image and subtracted from the raw fluorescence intensity. Negative background-corrected values were set to zero. For each sampled subarea, the mean background-corrected fluorescence intensity was calculated and used as one technical measurement. For each gene and condition, fluorescence intensity was summarized as mean ± standard error of the mean (S.E.M.). CA1 and Sub fluorescence intensities were compared using a two-sided Welch's *t*-test. Statistical significance was indicated as ns, $p \geq 0.05$; * $p < 0.05$; ** $p < 0.01$; *** $p < 0.001$; and **** $p < 0.0001$. These measurements represent image-level technical quantification of regional fluorescence enrichment.

Data Availability

The datasets supporting the findings of this study are available from public repositories. The high-resolution spatial transcriptomic (Stereo-seq) data have been deposited in the CNGB Nucleotide Sequence Archive (CNSA) of the China National GeneBank DataBase (CNGBdb) under accession code CNP0005077. The publicly available mouse brain spatial transcriptomic data used for cross-species validation were obtained from Han et al. (2025) [28] and are available from CNSA under accession code CNP0003837. The RNAscope images evaluated in this study, together with the *in situ* hybridization images showing *Fn1* and *Etv1* expression in the mouse brain obtained from the Allen Mouse Brain Atlas, are also publicly available through the same GitHub repository (ST-Human-Hippocampus-Boundary/tree/main/Validation_Images). The single-nucleus RNA sequencing (snRNA-seq) data utilized in this study were obtained from the Gene Expression Omnibus (GEO) under accession number GSE186538, which includes transcriptomic profiles microdissected from multiple human hippocampal and parahippocampal regions, including the dentate gyrus (DG), CA2–4, CA1, Sub, and entorhinal cortex (EC).

Availability of source code and requirements

Project name: ST-Human-Hippocampus-Boundary

Project homepage: https://github.com/L-Yuyang/2025_ST-Human-Hippocampus-Boundary

License: MIT license

Operating system: Linux, macOS, Windows

Programming language: R and Python

Package management: CRAN, Bioconductor and Conda

Hardware requirements: Recommended at least 8-core CPU and 64 GB RAM for processing high-resolution Stereo-seq dataset

Acknowledgments

This work was supported by National Key R&D Program of China (2021YFA0805100), Key Program of the National Natural Science Foundation of China (32530027), the National Science and Technology Innovation 2030 Major Program (2021ZD0204400). We thank BGI for hosting various datasets from this study, DCS Cloud (https://cloud.stomics.tech/) for providing the computational resources and software support necessary for this study.

Author contributions

L.W., J.Z., and L.H. designed the research; Y.L. and Y.H. analyzed the sequencing data and wrote the manuscript. L.W. and Y.L. edited the manuscript. J.Z. and P.W. provided the tissue sections for RNAscope used in this study. S.L., Y.W., P.W., C.H., C.H., Q.T., L.Z., X.X. and L.L. contributed

to unpublished reagents/analytic tools/advice. L.W., S.L., J.Z., and L.H. acquired funding and reviewed the manuscript. All authors read and approved the manuscript for submission.

Competing interests

The authors are affiliated with BGI, the institutional sponsor of GigaScience. All editorial handling and peer review for this manuscript were conducted independently by the journal's editorial team, in accordance with the journal's standard editorial policies and procedures, to ensure transparency, objectivity, and impartiality.

References

[1] Iglesias, J. E. *et al.* A computational atlas of the hippocampal formation using ex vivo, ultra-high resolution MRI: Application to adaptive segmentation of in vivo MRI. *NeuroImage* **115**, 117–137 (2015). https://doi.org/10.1016/j.neuroimage.2015.04.042

[2] Tosches, M. A. *et al.* Evolution of pallium, hippocampus, and cortical cell types revealed by single-cell transcriptomics in reptiles. *Science* **360**, 881–888 (2018). https://doi.org/10.1126/science.aar4237

[3] Lisman, J. *et al.* Viewpoints: how the hippocampus contributes to memory, navigation and cognition. *Nature neuroscience* **20**, 1434–1447 (2017). https://doi.org/10.1038/nn.4661

[4] Donato, F. *et al.* The Ontogeny of Hippocampus-Dependent Memories. *The Journal of neuroscience : the official journal of the Society for Neuroscience* **41**, 920–926 (2021). https://doi.org/10.1523/jneurosci.1651-20.2020

[5] Berdugo-Vega, G., Dhingra, S. & Calegari, F. Sharpening the blades of the dentate gyrus: how adult-born neurons differentially modulate diverse aspects of hippocampal learning and memory. *The EMBO journal* **42**, e113524 (2023). https://doi.org/10.15252/embj.2023113524

[6] O'Mara, S. M., Commins, S., Anderson, M. & Gigg, J. The subiculum: a review of form, physiology and function. *Progress in neurobiology* **64**, 129–155 (2001). https://doi.org/10.1016/s0301-0082(00)00054-x

[7] Sun, Y. *et al.* CA1-projecting subiculum neurons facilitate object-place learning. *Nature neuroscience* **22**, 1857–1870 (2019). https://doi.org/10.1038/s41593-019-0496-y

[8] Mizuseki, K. & Kitanishi, T. J. C. O. i. N. Oscillation-coordinated, noise-resistant information distribution via the subiculum. *Current Opinion in Neurobiology* **75**, 102556 (2022). https://doi.org/10.1016/j.conb.2022.102556

[9] Huxter, J. R., Senior, T. J., Allen, K. & Csicsvari, J. J. N. n. Theta phase–specific codes for two-dimensional position, trajectory and heading in the hippocampus. *Nature neuroscience* **11**, 587–594 (2008). https://doi.org/10.1038/nn.2106

[10] Kitanishi, T., Umaba, R. & Mizuseki, K. Robust information routing by dorsal subiculum neurons. *Sci Adv* **7** (2021). https://doi.org/10.1126/sciadv.abf1913

[11] Romani, A. *et al.* Community-based reconstruction and simulation of a full-scale model of the rat hippocampus CA1 region. *PLoS biology* **22**, e3002861 (2024). https://doi.org/10.1371/journal.pbio.3002861

[12] Liu, X. *et al.* Multi-omics and high-spatial-resolution omics: deciphering complexity in neurological disorders. *GigaScience* **14**, giaf137 (2025). https://doi.org/10.1093/gigascience/giaf137

[13] Cao, L. *et al.* Deciphering spatial domains from spatially resolved transcriptomics with Siamese graph autoencoder. *GigaScience* **13**, giae003 (2024). https://doi.org/10.1093/gigascience/giae003

[14] Zhou, L. *et al.* Unveiling patterns in spatial transcriptomics data: a novel approach utilizing graph attention autoencoder and multiscale deep subspace clustering network. *GigaScience* **14**, giae103 (2025). https://doi.org/10.1093/gigascience/giae103

[15] Kang, J. *et al.* Exploring the cellular and molecular basis of murine cardiac development through spatiotemporal transcriptome sequencing. *GigaScience* **14**, giaf012 (2025). https://doi.org/10.1093/gigascience/giaf012

[16] Rosenblum, E. W., Williams, E. M., Champion, S. N., Frosch, M. P. & Augustinack, J. C. The prosubiculum in the human hippocampus: A rostrocaudal, feature-driven, and systematic approach. *The Journal of comparative neurology* **532**, e25604 (2024). https://doi.org/10.1002/cne.25604

[17] Adler, D. H. *et al.* Histology-derived volumetric annotation of the human hippocampal subfields in postmortem MRI. *NeuroImage* **84**, 505–523 (2014). https://doi.org/10.1016/j.neuroimage.2013.08.067

[18] Wang, P. *et al.* Molecular pathways and diagnosis in spatially resolved Alzheimer's hippocampal atlas. *Neuron* **113**, 2123–2140.e2129 (2025). https://doi.org/10.1016/j.neuron.2025.03.002

[19] El Hayek, L. *et al.* Disruption of the autism gene and chromatin regulator KDM5A alters hippocampal cell identity. *Sci Adv* **9**, eadi0074 (2023). https://doi.org/10.1126/sciadv.adi0074

[20] Zhao, E. *et al.* Spatial transcriptomics at subspot resolution with BayesSpace. *Nature biotechnology* **39**, 1375–1384 (2021). https://doi.org/10.1038/s41587-021-00935-2

[21] Franjic, D. *et al.* Transcriptomic taxonomy and neurogenic trajectories of adult human, macaque, and pig hippocampal and entorhinal cells. *Neuron* **110**, 452–469.e414 (2022). https://doi.org/10.1016/j.neuron.2021.10.036

[22] Fei, F. *et al.* Fibronectin1 - Expressing Subicular Circuits Selectively Govern the Retrieval of Novel Object Recognition. *Adv Sci (Weinh)* **13**, e16399 (2026). https://doi.org/10.1002/advs.202516399

[23] Tao, Y. *et al.* Erbin regulates NRG1 signaling and myelination. *Proceedings of the National Academy of Sciences of the United States of America* **106**, 9477–9482 (2009). https://doi.org/10.1073/pnas.0901844106

[24] Podvin, S., Wojnicz, A. & Hook, V. Human brain gene expression profiles of the cathepsin V and cathepsin L cysteine proteases, with the PC1/3 and PC2 serine proteases, involved in neuropeptide production. *Heliyon* **4**, e00673 (2018).

https://doi.org/10.1016/j.heliyon.2018.e00673

[25] Rigter, P. M. F. *et al.* Role of CAMK2D in neurodevelopment and associated conditions. *American journal of human genetics* **111**, 364–382 (2024). https://doi.org/10.1016/j.ajhg.2023.12.016

[26] Thompson, J. R. *et al.* An integrated single-nucleus and spatial transcriptomics atlas reveals the molecular landscape of the human hippocampus. *Nature neuroscience* **28**, 1990–2004 (2025). https://doi.org/10.1038/s41593-025-02022-0

[27] Lein, E. S. *et al.* Genome-wide atlas of gene expression in the adult mouse brain. *Nature* **445**, 168–168 (2007). https://doi.org/10.1038/nature05453

[28] Han, L. *et al.* Single-cell spatial transcriptomic atlas of the whole mouse brain. *Neuron* **113**, 2141–2160. e2149 (2025). https://doi.org/10.1016/j.neuron.2025.02.015

[29] Cadonic, C., Sabbir, M. G. & Albensi, B. C. Mechanisms of Mitochondrial Dysfunction in Alzheimer's Disease. *Molecular neurobiology* **53**, 6078–6090 (2016). https://doi.org/10.1007/s12035-015-9515-5

[30] Adav, S. S., Park, J. E. & Sze, S. K. Quantitative profiling brain proteomes revealed mitochondrial dysfunction in Alzheimer's disease. *Molecular brain* **12**, 8 (2019). https://doi.org/10.1186/s13041-019-0430-y

[31] Morabito, S., Reese, F., Rahimzadeh, N., Miyoshi, E. & Swarup, V. hdWGCNA identifies co-expression networks in high-dimensional transcriptomics data. *Cell reports methods* **3**, 100498 (2023). https://doi.org/10.1016/j.crmeth.2023.100498

[32] Hong, B. V. *et al.* High-Density Lipoprotein Changes in Alzheimer's Disease Are APOE Genotype-Specific. *Biomedicines* **10** (2022). https://doi.org/10.3390/biomedicines10071495

[33] Fleeman, R. M., Kuhn, M. K., Chan, D. C. & Proctor, E. A. Apolipoprotein E ε4 modulates astrocyte neuronal support functions in the presence of amyloid-β. *Journal of neurochemistry* **165**, 536–549 (2023). https://doi.org/10.1111/jnc.15781

[34] Azarnia Tehran, D. *et al.* Selective endocytosis of Ca(2+)-permeable AMPARs by the Alzheimer's disease risk factor CALM bidirectionally controls synaptic plasticity. *Sci Adv* **8**, eabl5032 (2022). https://doi.org/10.1126/sciadv.abl5032

[35] Shen, R. *et al.* Spatial-ID: a cell typing method for spatially resolved transcriptomics via transfer learning and spatial embedding. *Nature communications* **13**, 7640 (2022). https://doi.org/10.1038/s41467-022-35288-0

[36] Ding, S. L. Comparative anatomy of the prosubiculum, subiculum, presubiculum, postsubiculum, and parasubiculum in human, monkey, and rodent. *The Journal of comparative neurology* **521**, 4145–4162 (2013). https://doi.org/10.1002/cne.23416

[37] Ishizuka, N. J. J. o. C. N. Laminar organization of the pyramidal cell layer of the

subiculum in the rat. *The Journal of comparative neurology* **435**, 89–110 (2001). https://doi.org/10.1002/cne.1195

[38] Yushkevich, P. A. *et al.* Quantitative comparison of 21 protocols for labeling hippocampal subfields and parahippocampal subregions in in vivo MRI: towards a harmonized segmentation protocol. *NeuroImage* **111**, 526–541 (2015). https://doi.org/10.1016/j.neuroimage.2015.01.004

[39] Cembrowski, M. S. *et al.* The subiculum is a patchwork of discrete subregions. *eLife* **7**, e37701 (2018). https://doi.org/10.7554/eLife.37701

[40] Ding, S.-L. *et al.* Distinct transcriptomic cell types and neural circuits of the subiculum and prosubiculum along the dorsal-ventral axis. *Cell reports* **31**, 107648 (2020). https://doi.org/10.1016/j.celrep.2020.107648

[41] González-Arnay, E. *et al.* Immunohistochemical field parcellation of the human hippocampus along its antero-posterior axis. *Brain structure & function* **229**, 359–385 (2024). https://doi.org/10.1007/s00429-023-02725-9

[42] Maynard, K. R. *et al.* Transcriptome-scale spatial gene expression in the human dorsolateral prefrontal cortex. *Nature neuroscience* **24**, 425–436 (2021). https://doi.org/10.1038/s41593-020-00787-0

[43] Lv, T. *et al.* EAGS: efficient and adaptive Gaussian smoothing applied to high-resolved spatial transcriptomics. *GigaScience* **13**, giad097 (2024). https://doi.org/10.1093/gigascience/giad097

[44] Ding, S. L. & Van Hoesen, G. W. J. J. o. C. N. Organization and detailed parcellation of human hippocampal head and body regions based on a combined analysis of cyto- and chemoarchitecture. *The Journal of comparative neurology* **523**, 2233–2253 (2015). https://doi.org/10.1002/cne.23786

[45] Pitceathly, R. D. *et al.* NDUFA4 mutations underlie dysfunction of a cytochrome c oxidase subunit linked to human neurological disease. *Cell reports* **3**, 1795–1805 (2013). https://doi.org/10.1016/j.celrep.2013.05.005

[46] Williams, C. G., Lee, H. J., Asatsuma, T., Vento-Tormo, R. & Haque, A. J. G. m. An introduction to spatial transcriptomics for biomedical research. *Genome medicine* **14**, 68 (2022). https://doi.org/10.1186/s13073-022-01075-1

[47] Hyman, B. T. *et al.* National Institute on Aging–Alzheimer's Association guidelines for the neuropathologic assessment of Alzheimer's disease. *Alzheimer's & Dementia* **8**, 1–13 (2012). https://doi.org/10.1016/j.jalz.2011.10.007

[48] Rao, A., Barkley, D., França, G. S. & Yanai, I. J. N. Exploring tissue architecture using spatial transcriptomics. *Nature* **596**, 211–220 (2021). https://doi.org/10.1038/s41586-021-03634-9

[49] Gong, C. *et al.* SAW: an efficient and accurate data analysis workflow for Stereo-seq spatial transcriptomics. *GigaByte* **2024**, 1–12 (2024). https://doi.org/10.46471/gigabyte.111

[50] Zhang, B. *et al.* Generating single-cell gene expression profiles for high-resolution spatial transcriptomics based on cell boundary images. *GigaByte* **2024**, 1–13 (2024). https://doi.org/10.46471/gigabyte.110

[51] Yu, G., Wang, L. G., Han, Y. & He, Q. Y. clusterProfiler: an R package for comparing biological themes among gene clusters. *Omics : a journal of integrative biology* **16**, 284–287 (2012). https://doi.org/10.1089/omi.2011.0118

[52] Shannon, P. *et al.* Cytoscape: a software environment for integrated models of biomolecular interaction networks. *Genome research* **13**, 2498–2504 (2003). https://doi.org/10.1101/gr.1239303

[53] Chin, C. H. *et al.* cytoHubba: identifying hub objects and sub-networks from complex interactome. *BMC systems biology* **8** **Suppl** **4**, S11 (2014). https://doi.org/10.1186/1752-0509-8-s4-s11

Click here to access/download
Supplementary Material
Response_to_Reviewers.docx

Click here to access/download
Supplementary Material
05_SupplementaryTables_260718-submit.xlsx

CoverLetter

Click here to access/download;Personal Cover;CoverLetter.docx

Dear Dr. Zhang and esteemed reviewers,

Thank you for the opportunity to revise our manuscript entitled "*A Molecularly Anchored Spatial Transcriptomic Framework for Precise CA1–Subiculum Parcellation and Region-Resolved Analysis in Alzheimer's Disease*" (Manuscript ID: GIGA-D-26-00145).

We sincerely appreciate the time and effort that you and the reviewers have dedicated to evaluating our work. The constructive and insightful comments have helped us substantially improve the clarity, rigor, reproducibility, and overall presentation of the study.

In the revised manuscript, we have addressed all reviewer comments point by point and have made substantial revisions throughout the manuscript. In particular, we have clarified the origin and relationship of the Stereo-seq, snRNA-seq, 10X Genomics, and RNAscope datasets; expanded the Methods and Supplementary Tables to provide more transparent tissue-selection criteria, sample metadata, and quality-control information; added technical validation of cell segmentation, cell-type annotation, and chip-level sequencing quality; and included donor-level and pseudobulk analyses to support the bin-level spatial findings. We have also strengthened the orthogonal validation of representative Sub-enriched markers through quantitative RNAscope analysis and independent spatial and anatomical reference datasets, while more clearly defining the scope and limitations of these validation analyses. In addition, we have refined our description of the CA1–subiculum interface as a gradual molecular transition zone and substantially revised the Discussion to improve the biological interpretation, acknowledge study limitations, and clarify the relevance of molecularly informed CA1–subiculum parcellation in Alzheimer's disease research.

For ease of reference, the reviewers' comments are shown in regular, our responses are provided in *blue italics*, and the corresponding revisions in the manuscript are highlighted in blue.

We hope that the revised manuscript is now suitable for consideration for publication in *GigaScience*, and we thank you again for your time and attention.

Yours sincerely,

Lifang Wang on behalf of all co-authors

Key Laboratory of Brain Cell Mapping of Zhejiang Province, BGI Research, Hangzhou, Zhejiang 310030, China
E-mail: wanglifang@genomics.cn
