# Supplementary material for: A molecularly anchored spatial transcriptomic framework for precise CA1–subiculum parcellation and region-resolved analysis in Alzheimer’s disease
Source: Gigascience. 2026 Sep 17;15:giag094. doi: 10.1093/gigascience/giag094 (PMC13674357; doi:10.1093/gigascience/giag094)
Supplement: Supplementary file 4 [file giag094_reviewer_1_report_original_submission.pdf]

Reviewer Report

Title: A Molecularly Anchored Spatial Transcriptomic Framework for Precise CA1â€"Subiculum Parcellation and Region-Resolved Analysis in Alzheimer's Disease

Version: Original Submission **Date:** 6/2/2026

Reviewer name: Reviewer 1

Reviewer Comments to Author:

Manuscript #GIGA-D-26-00145 "A Molecularly Anchored Spatial Transcriptomic Framework for Precise CA1-Subiculum Parcellation and Region-Resolved Analysis in Alzheimer's Disease" by Y. Liu et al., leverages publicly available data obtained by spatial transcriptomics (Stereo-seq) and snRNA-seq from Wang et al., 2025 and Franjic et al., 2022 to establish boundaries of key hippocampal regions, namely the CA1 sector and the subiculum. Discerning the anatomic boundaries between the CA1 sector and subiculum are notoriously difficult, especially within the postmortem human brain. The authors utilize several advanced bioinformatics techniques and AI supported programs to identify specific genetic markers (e.g., FN1 with support from ETV1, PCSK1, and CAMK2G) to delineate CA1-subiculum boundaries, and present a viable panel of potential markers for demarcation. This mapping heuristic may be useful in the context of normal subjects as well as inform neurodegenerative disorders including Alzheimer's disease (AD). There is a lot of information to unpack in this study! The authors are urged to reconsider the organization of the manuscript for readership. Below are major and minor considerations to improve an interesting and potentially impactful manuscript.

Major

1. Manuscript stewardship. The entire manuscript would benefit from a hard copyedit from a native English speaking collaborator who can help shape the narrative. Reliance on online/AI grammar is not recommended as the issues of organization and higher order integration require collaborative assistance.
2. Tissue sample access. Stereo-seq data was obtained from Wang et al., 2025. RNAscope validation appears to have been conducted on a subset of tissues. It is unclear if the authors had direct access to the tissues or if they are from another cohort. This needs to be specified for the assays. Moreover, the exact relationship with Wang et al., 2025 needs to be stated in the Methods and whether (or not) this study is a reanalysis of previously acquired data or a follow-up of shared tissues.
3. Tissue handling. The authors need to state specifically how tissues were handled and prepared including tissue fixation procedures, size and number of sections used for snRNA-seq and RNAscope.
4. Tissue selection criteria. An explanation of how tissues were included (and excluded) is warranted. The Reviewer suggests a Supplementary image panel of what is acceptable or not acceptable tissue quality for postmortem hippocampal and subiculum sections.
5. AI assistance verification. Was manual validation of AI-generated segmentation performed? Greater details regarding QA/QC is strongly recommended to indicate validity of the AI techniques used.
6. RNAscope. The RNAscope approach requires greater detail (e.g., case demographics, technical strategy) along with the method of comparison to spatially derived data from Wang et al., 2025.

7. Discussion. Despite the large amount of data and supportive validation, the Discussion is skeletal. The authors are urged to rewrite the Discussion to reflect the impact of the current boundary delineation findings as well as reframe postmortem human tissue based studies attempting to define the CA1-subiculum continuum in the context of existing literature.

Minor

1. Please restructure the manuscript for conventional readership by presenting approaches in the Methods, findings in the Results, and interpretations in the Discussion. The original submission has these sections dispersed throughout the text.
2 . Relocate Source code from the Methods to the Data Availability section.
3. Are images of the sections that the authors evaluated publicly available? Please upload them to a repository such as www.synapse.org.
4. There is an enormous amount of spatial and sequence data. Please curate genelists for ease of readership and consider making them available as Supplementary material.
5. Little information is given to the AD cohort. Since AD hallmark pathology is present in the CA1 and subiculum, how is this expected to impact the ability to delineate these boundaries?

Methods

Are the methods appropriate to the aims of the study, are they well described, and are necessary controls included? Choose an item.

Conclusions

Are the conclusions adequately supported by the data shown? Choose an item.

Reporting Standards

Does the manuscript adhere to the journal's guidelines on [minimum standards of reporting?]() Choose an item.

Choose an item.

Statistics

Are you able to assess all statistics in the manuscript, including the appropriateness of statistical tests used? Choose an item.

Quality of Written English

Please indicate the quality of language in the manuscript: Choose an item.

Declaration of Competing Interests

Please complete a declaration of competing interests, considering the following questions:

- Have you in the past five years received reimbursements, fees, funding, or salary from an organisation that may in any way gain or lose financially from the publication of this manuscript, either now or in the future?
- Do you hold any stocks or shares in an organisation that may in any way gain or lose financially from the publication of this manuscript, either now or in the future?
- Do you hold or are you currently applying for any patents relating to the content of the manuscript?
- Have you received reimbursements, fees, funding, or salary from an organization that holds or has applied for patents relating to the content of the manuscript?
- Do you have any other financial competing interests?
- Do you have any non-financial competing interests in relation to this paper?

If you can answer no to all of the above, write 'I declare that I have no competing interests' below. If your reply is yes to any, please give details below.

I declare that I have no competing interests.

I agree to the open peer review policy of the journal. I understand that my name will be included on my report to the authors and, if the manuscript is accepted for publication, my named report including any attachments I upload will be posted on the website along with the authors' responses. I agree for my report to be made available under an Open Access Creative Commons CC-BY license (http://creativecommons.org/licenses/by/4.0/). I understand that any comments which I do not wish to be included in my named report can be included as confidential comments to the editors, which will not be published.

I agree to the open peer review policy of the journal

To further support our reviewers, we have joined with Publons, where you can gain additional credit to further highlight your hard work (see: https://publons.com/journal/530/gigascience). On publication of this paper, your review will be automatically added to Publons, you can then choose whether or not to claim your Publons credit. I understand this statement.

Yes Choose an item.
