# Supplementary material for: A molecularly anchored spatial transcriptomic framework for precise CA1–subiculum parcellation and region-resolved analysis in Alzheimer’s disease
Source: Gigascience. 2026 Sep 17;15:giag094. doi: 10.1093/gigascience/giag094 (PMC13674357; doi:10.1093/gigascience/giag094)
Supplement: Supplementary file 6 [file giag094_reviewer_2_report_original_submission.pdf]

Reviewer Report

Title: A Molecularly Anchored Spatial Transcriptomic Framework for Precise CA1â€“Subiculum Parcellation and Region-Resolved Analysis in Alzheimer's Disease

Version: Original Submission **Date:** 6/10/2026

Reviewer name: Reviewer 2

Reviewer Comments to Author:

Summary
This study addresses the persistent challenge of reliably defining the CA1-subiculum boundary in hippocampal research. To resolve inconsistencies, the authors employ complementary methods Stereo-seq spatial transcriptomics and single-nucleus RNA-seq on 12 human donors (6 with Alzheimer's disease and 6 controls). They identify FN1 as a molecular marker of the subiculum, with support from ETV1, PCSK1, and CAMK2G, and validate these findings using RNAscope and an independent dataset. Applying this molecular boundary to Alzheimer's tissue reveals region-specific changes, most notably increased mitochondrial and energy-metabolism gene expression in the subiculum. The study is interesting and potentially valuable, though several issues remain to be addressed.

Major

1. Clarify the origin of the data. The paper reads as if a new 12-donor dataset was generated, but the Stereo-seq, snRNA-seq, and 10X data all come from earlier studies (Wang 2025; Franjic 2022; Thompson 2025); only the RNAscope is new. State this plainly and explain how the study relates to Wang 2025.
2. Strengthen the statistical analysis. Testing a large number of bins or cells does not compensate for the small cohort of 6 Alzheimer's disease donors and 6 control donors, as the donor is the appropriate unit of analysis. Therefore, the main results should be recomputed at the donor level using suitable models, such as pseudobulk or mixed-effects approaches.
3. Provide direct evidence that FN1 marks neurons. Although FN1 is primarily expressed in the extracellular matrix and vasculature bordering the subiculum, the observed elevated FN1 signal may reflect non-neuronal contamination. Therefore, the authors should provide direct evidence that FN1 marks subicular neurons.
4.No evidence is provided that the Alzheimer's and control groups were matched for post-mortem interval, RNA quality, or cause of death. Several leading Alzheimer's disease genes (FOS, JUNB, NR4A, NPAS4) are stress- and death-response genes, so bin-level results may confound cell loss with real expression changes. These factors weaken the metabolic findings.
5. Resolve the conflicting boundary description. While graded markers and proximal/distal neuron subtypes suggest a gradual transition, the text describes a sharp boundary. This contradiction should be addressed, and strong claims about boundary sharpness should be tempered accordingly.

Minor

6. Validation is limited: RNAscope was performed for only two genes, assessed visually with minimal sampling, and 10X validation is incomplete. The legend of Extended Data Fig. 1 reads "Con," while the figure is titled "AD"; this requires correction.

7. Donor information is incomplete: APOE genotype, sex, age, and post-mortem interval are not reported, and the Alzheimer's disease cases span a broad pathological range (Braak III-V) within a small cohort.
8. Inaccurate method description. The manuscript describes hdWGCNA as characterizing gene correlation patterns in microarray samples. This describes the original WGCNA; hdWGCNA was specifically designed for high-dimensional single-cell and spatial transcriptomic data, which is the data type used here. Please clarify

Methods

Are the methods appropriate to the aims of the study, are they well described, and are necessary controls included? Choose an item.

Conclusions

Are the conclusions adequately supported by the data shown? Choose an item.

Reporting Standards

Does the manuscript adhere to the journal's guidelines on [minimum standards of reporting?]() Choose an item.

Choose an item.

Statistics

Are you able to assess all statistics in the manuscript, including the appropriateness of statistical tests used? Choose an item.

Quality of Written English

Please indicate the quality of language in the manuscript: Choose an item.

Declaration of Competing Interests

Please complete a declaration of competing interests, considering the following questions:

- Have you in the past five years received reimbursements, fees, funding, or salary from an organisation that may in any way gain or lose financially from the publication of this manuscript, either now or in the future?
- Do you hold any stocks or shares in an organisation that may in any way gain or lose financially from the publication of this manuscript, either now or in the future?
- Do you hold or are you currently applying for any patents relating to the content of the manuscript?

- Have you received reimbursements, fees, funding, or salary from an organization that holds or has applied for patents relating to the content of the manuscript?
- Do you have any other financial competing interests?
- Do you have any non-financial competing interests in relation to this paper?

If you can answer no to all of the above, write 'I declare that I have no competing interests' below. If your reply is yes to any, please give details below.

I declare that I have no competing interests

I agree to the open peer review policy of the journal. I understand that my name will be included on my report to the authors and, if the manuscript is accepted for publication, my named report including any attachments I upload will be posted on the website along with the authors' responses. I agree for my report to be made available under an Open Access Creative Commons CC-BY license (http://creativecommons.org/licenses/by/4.0/). I understand that any comments which I do not wish to be included in my named report can be included as confidential comments to the editors, which will not be published.

I agree to the open peer review policy of the journal

To further support our reviewers, we have joined with Publons, where you can gain additional credit to further highlight your hard work (see: https://publons.com/journal/530/gigascience). On publication of this paper, your review will be automatically added to Publons, you can then choose whether or not to claim your Publons credit. I understand this statement.

Yes Choose an item.
